# Modulating nonequilibrium electron*–*phonon interactions and energy relaxation in MXenes by surface-anchored Mo_3_S_7_ nanoclusters

**DOI:** 10.1038/s41467-026-75614-4

**Published:** 2026-07-16

**Authors:** Jiaxu Zhang, Rafael Muñoz-Mármol, Zijie Xiao, Dongqi Li, Shuai Fu, Xiaodong Li, Juliane Scheiter, Andrea Iudica, Valentino Romano, Eva A. A. Pogna, Pengfei Cao, Jingwei Du, Xingyuan Chu, Francesco Scotognella, Nicolás Pérez, Kornelius Nielsch, Giuseppe Maria Paternò, Lei Gao, Mischa Bonn, Minghao Yu, Xinliang Feng

**Affiliations:** 1 Faculty of Chemistry and Food Chemistry & Center for Advancing Electronics Dresden (cfaed), Technische Universität Dresden, Dresden, Germany; 2 Department of Physics, Politecnico di Milano, Milan, Italy; 3 Instituto Universitario de Materiales, University of Alicante, San Vicente del Raspeig, Spain; 4 Max Planck Institute for Polymer Research, Mainz, Germany; 5 Max Planck Institute of Microstructure Physics, Halle (Saale), Germany; 6 Leibniz Institute for Solid State and Materials Research Dresden, Dresden, Germany; 7 CNR, Istituto di Fotonica e Nanotecnologie, Milan, Italy; 8 Ernst Ruska-Centre for Microscopy and Spectroscopy with Electrons, Forschungszentrum Jülich, Jülich, Germany; 9 Department of Applied Science and Technology, Politecnico di Torino, Torino, Italy; 10 Institute of Materials Science, Institute of Applied Physics, Technische Universität Dresden, Dresden, Germany; 11 Center for Nanoscience and Technology, Istituto Italiano di Tecnologia, Milano, Italy

**Keywords:** Two-dimensional materials, Excited states, Electronic properties and materials

## Abstract

Electron–phonon (e–ph) interactions govern photoinduced nonequilibrium dynamics of MXenes, determining hot-carrier relaxation and parasitic heat accumulation. However, strategies to deliberately modulate these interactions through chemical control, together with mechanistic understanding, remain underexplored. Here, we demonstrate the effective modulation of nonequilibrium e–ph interactions in Ti_3_C_2_T_*x*_ MXene via surface-anchored Mo_3_S_7_ nanoclusters, which introduce a rapid energy harvesting pathway competing with intrinsic e–ph relaxation. Using mild ligand substitution, Mo_3_S_7_ nanoclusters are densely and homogenously anchored onto Ti_3_C_2_T_*x*_ via coordination bonding between Mo centers and O-terminations. Femtosecond transient absorption and optical-pump terahertz-probe spectroscopy reveal an ultrafast, sub-100 fs nonthermal electron and/or energy extraction, with efficiency increasing from ~28.6 % at 1.55 eV to ~38.2 % at 3.88 eV. This excitation-energy-dependent enhancement is enabled by improved energetic alignment between hot electrons in Ti_3_C_2_T_*x*_ and the conduction-band manifold of Mo_3_S_7_. The competitive depletion of nonthermal electrons suppresses coherent A_1g_ phonon excitation, reducing effective e–ph interactions. Our study offers a viable strategy for modulating e–ph interactions in MXenes, advancing hot carrier relaxation and thermal management in next-generation optoelectronic devices.

## Abstract (web summary)

Using ultrafast spectroscopy, the authors reveal that surface-anchored Mo_3_S_7_ nanoclusters redirect hot carrier relaxation in MXenes, suppressing coherent phonons and electron–phonon couplings to reduce energy loss and improve thermal management.

## Introduction

Light–matter interactions in two-dimensional (2D) materials give rise to distinctive optical responses and intriguing quantum phenomena, establishing them as ideal platforms for exploring many-body physics and advancing optoelectronic technologies^1,2^. Among these, 2D transition metal carbides and/or nitrides, collectively known as MXenes, stand out due to their high free-electron densities and broadband surface plasmon (SP) excitations^3,4^. These features position MXenes as compelling materials for a broad range of applications^5,6^, including plasmon-enhanced photodetectors^7^, additives in solar harvesting layers^8^, and active components in phototransistors^9^.

Progress in device engineering, however, hinges on a fundamental understanding of how photo-excited energy is redistributed and dissipated, an essential step toward elucidating thermal management and energy-loss pathways^10^. In this context, time-resolved spectroscopic studies have increasingly focused on unveiling nonequilibrium carrier dynamics in MXenes^11–15^. Recent studies have identified a mode-selective, excitation-dependent e–ph coupling mechanism in far-from-equilibrium Ti_3_C_2_T_*x*_^11^, characterized by ultrafast energy relaxation and a remarkably strong coupling constant that exceeds those in conventional metals and semimetals^16,17^. Specifically, under SP excitation, nearly half of the nonthermal electrons bypass thermalization and couple directly to coherent phonons within ~60 fs. Of these, the majority funnel into the out-of-plane A_1g_ phonon mode, yielding a pronounced dimensionless coupling constant of *λ* = 1.62 ± 0.33. In contrast, coupling to the in-plane E_g_ mode is markedly slower (1–2 ps) and weaker (*λ* = 0.054 ± 0.018). Meanwhile, interband transition (IBT) excitation couples exclusively to the A_1g_ mode. These observations highlight the critical role of e–ph interactions in determining the energy relaxation dynamics of MXenes, processes that significantly impact the performance of optoelectronic devices.

Clearly, the pronounced e–ph coupling in the nonequilibrium regime poses challenges for MXene applications, as it suppresses the efficient utilization of nonthermal electrons and dissipates substantial energy into the lattice as heat. Given the intrinsically low out-of-plane thermal conductivity of MXenes^18^, this parasitic heat accumulation can compromise both device efficiency and long-term operational stability. Strategies that actively modulate e–ph interactions under nonequilibrium conditions are therefore highly desirable for enhancing hot electron utilization and improving thermal management. Recent studies have revealed that photoexcitation of Ti_3_C_2_T_*x*_ transiently populates Ti *d*z^2^ orbitals^11,19^, redistributing electron density along the z-axis. This redistribution promotes coupling with the A_1g_ phonon mode, enabling direct interaction between nonthermal electrons and out-of-plane vibrations. It has been hypothesized that introducing competitive pathways to rapidly extract nonthermal electrons from the Ti *d*z^2^ orbitals, on timescales that compete with those of phonon interaction, could reshape the excited-state distribution and attenuate the coupling to the A_1g_ mode. Despite this conceptual framework, direct experimental demonstration of this modulation strategy remains scarce.

**Fig. 1:**
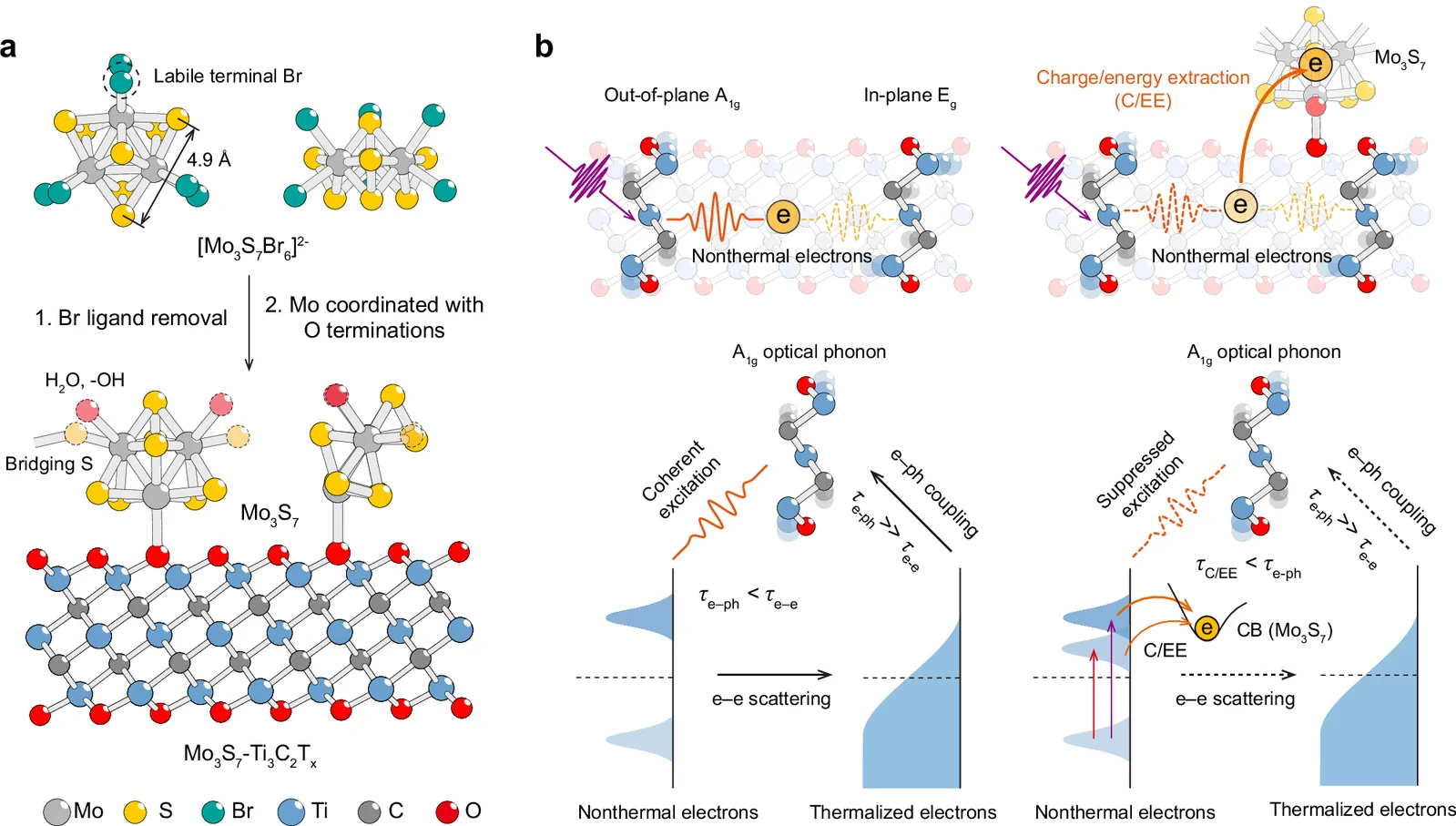
Synthesis route and modulation mechanism of nonequilibrium e–ph interaction in Mo_3_S_7_-Ti_3_C_2_T_*x*_. **a** Schematic of the ligand substitution strategy used to synthesize Mo_3_S_7_-Ti_3_C_2_T_*x*_, where Mo_3_S_7_ nanoclusters are anchored onto Ti_3_C_2_T_*x*_ via coordination with surface O-containing terminations. **b** Schematic of photoinduced energy relaxation pathways. In Ti_3_C_2_T_*x*_, photo-excited nonthermal electrons can bypass thermalization and directly couple to the A_1g_ coherent phonons (with only minor coupling to E_g_ under SP excitation). Following this, thermalized electrons undergo conventional e–ph coupling. Surface-anchored Mo_3_S_7_ nanoclusters introduce an ultrafast energy extraction channel, rapidly depleting nonthermal electrons that would otherwise couple to the A_1g_ phonon mode. By redirecting electron populations, this suppresses energy retention in the electronic system. Higher pump photon energies facilitate more efficient energy harvesting from Ti_3_C_2_T_*x*_ to Mo_3_S_7_.

Herein, we demonstrate that nonequilibrium e–ph interactions in Ti_3_C_2_T_*x*_ can be effectively modulated by anchoring Mo_3_S_7_ nanoclusters onto its surface. These nanoclusters offer a rapid, competitive pathway for capturing hot electrons, thereby disrupting the intrinsic e–ph relaxation pathways. Using a mild ligand substitution approach (Fig. 1a), Mo_3_S_7_ nanoclusters are densely and homogeneously anchored onto Ti_3_C_2_T_*x*_ through coordination bonds between Mo and O-containing terminations (denoted Mo_3_S_7_-Ti_3_C_2_T_*x*_). Femtosecond transient absorption (TA) spectroscopy combined with coherent phonon analysis reveals a π-phase inversion of the A_1g_ coherent phonon in Mo_3_S_7_-Ti_3_C_2_T_*x*_. This inversion, accompanied by a node in the oscillation amplitude at 580 nm, is attributed to coherent phonon-induced modulation of electronic states that differentially affect IBT and SP absorption. Moreover, Mo_3_S_7_-Ti_3_C_2_T_*x*_ exhibits probe-energy-dependent suppression of e–ph coupling, with sub-100 fs depletion of nonthermal electrons leading to up to ~60% reduction in the A_1g_ coherent phonon amplitude at a 400 nm probe. Complementary optical-pump terahertz (THz)-probe (OPTP) measurements corroborate this ultrafast interfacial charge/energy pathway and reveal its dependence on pump photon energy. Higher-energy excitation facilitates more efficient extraction, likely due to improved energetic alignment between the nonthermal electron distribution in Ti_3_C_2_T_*x*_ and accessible accepting states in the Mo_3_S_7_ nanoclusters (Fig. 1b). Beyond modulating electronic dynamics, Mo_3_S_7_ nanoclusters are shown to enhance interfacial thermal conductance, aiding heat dissipation between MXene layers. Collectively, these findings provide direct evidence that nonequilibrium e–ph interactions in Ti_3_C_2_T_*x*_ can be efficiently tuned through the rational design of interfacial charge/energy harvesting pathways. This strategy offers a powerful route for enhancing hot carrier utilization and optimizing thermal management in MXene-based optoelectronic devices.

## Results

### Anchoring Mo_3_S_7_ nanoclusters onto Ti_3_C_2_T_*x*_

**Fig. 2:**
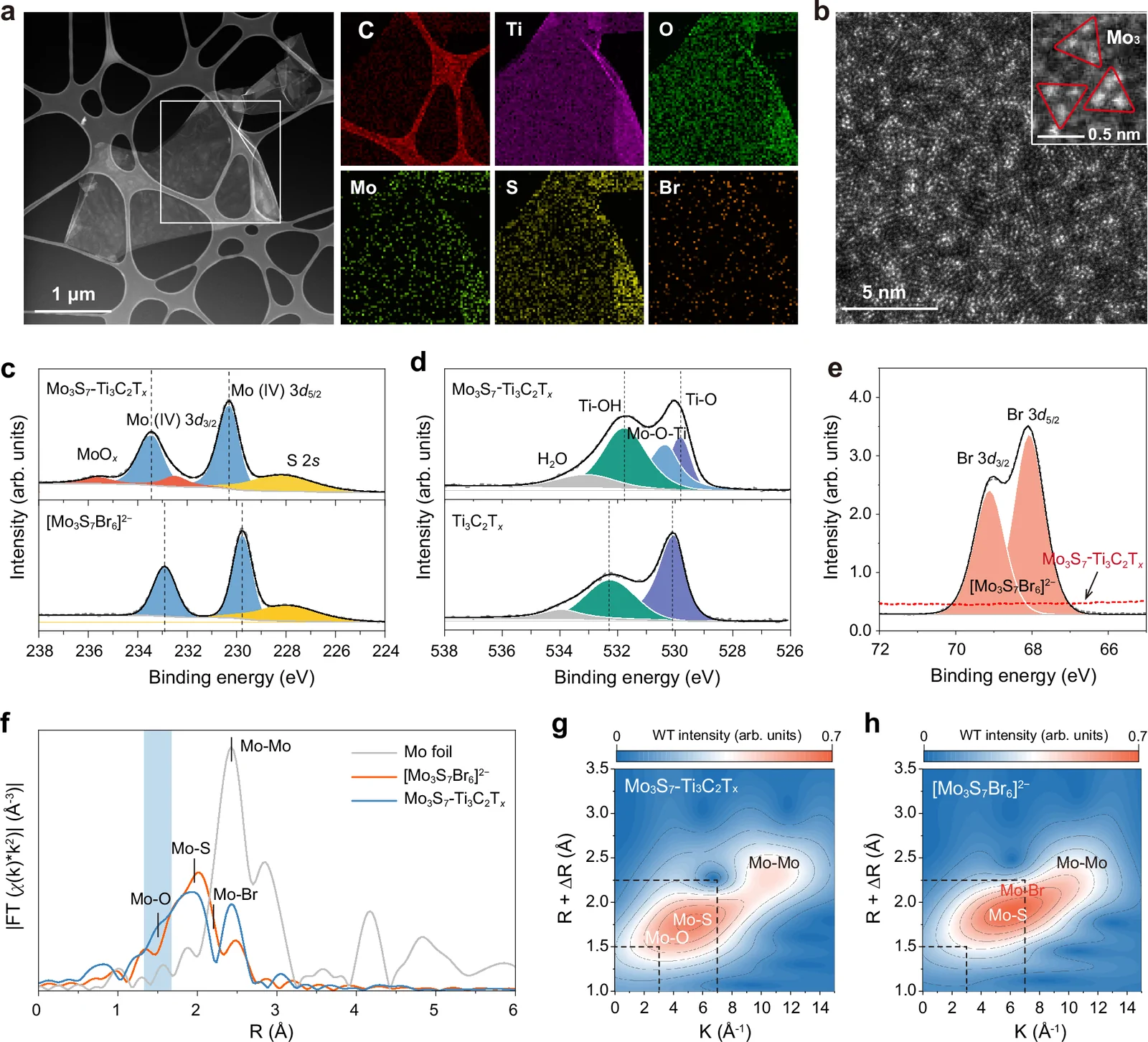
Structural characterizations of Mo_3_S_7_-Ti_3_C_2_T_*x*_. **a** HAADF–STEM image and corresponding elemental mapping of Mo_3_S_7_-Ti_3_C_2_T_*x*_. **b** Atomic-resolution HAADF-STEM image of Mo_3_S_7_-Ti_3_C_2_T_*x*_. The HAADF–STEM imaging experiments shown in **a** and **b** were independently repeated twice with similar results. **c** Mo 3 *d* XPS spectra of Mo_3_S_7_-Ti_3_C_2_T_*x*_ and [Mo_3_S_7_Br_6_]^2−^. **d** O 1 *s* XPS spectra of Mo_3_S_7_-Ti_3_C_2_T_*x*_ and Ti_3_C_2_T_*x*_. **e** Br 3 *d* XPS spectra of Mo_3_S_7_-Ti_3_C_2_T_*x*_ and [Mo_3_S_7_Br_6_]^2−^. **f** FT-EXAFS spectra of Mo foil, [Mo_3_S_7_Br_6_]^2−^, and Mo_3_S_7_-Ti_3_C_2_T_*x*_. Wavelet transform contour plots of **g** Mo_3_S_7_-Ti_3_C_2_T_*x*_ and **h** [Mo_3_S_7_Br_6_]^2−^. Source data are provided as a Source Data file.

The [Mo_3_S_7_Br_6_]^2−^ nanocluster, comprising a compact Mo_3_S_7_ core (~4.9 Å), is coordinated by six labile terminal Br ligands that can readily undergo nucleophilic substitution with strong Lewis-base ligands^20,21^. Leveraging this reactivity, we anchored Mo_3_S_7_ nanoclusters onto Ti_3_C_2_T_*x*_ surfaces via a light-assisted ligand exchange reaction (Fig. 1a). Under ambient illumination, photolytic cleavage of Mo–Br bonds exposes cationic Mo centers, which subsequently coordinate with O-terminated, Lewis-basic sites on Ti_3_C_2_T_*x*_ (Supplementary Fig. 1)^22^. High-resolution transmission electron microscopy images, along with the corresponding selected area electron diffraction pattern, confirm that the Mo_3_S_7_-Ti_3_C_2_T_*x*_ hybrid preserves the 2D morphology and crystallinity of the parent MXene (Supplementary Fig. 2). This structural integrity is further supported by high-angle annular dark-field scanning transmission electron microscopy (HAADF–STEM), which reveals a smooth surface, particulate-free surface, in contrast to degradation-prone MXene (Fig. 2a). Elemental mapping via energy-dispersive X-ray spectroscopy (EDS) uncovers homogeneous distribution of Mo and S across the entire flake, while the absence of Br signals confirms its effective removal during the substitution. Quantitative EDS analysis indicates an estimated Mo_3_S_7_ loading of approximately 8 wt% in the as-prepared Mo_3_S_7_-Ti_3_C_2_T_*x*_ hybrid (Supplementary Fig. 3a). Furthermore, atomic-resolution HAADF–STEM images identify distinct Z-contrast signatures of Mo atoms evenly dispersed on the Ti_3_C_2_T_*x*_ surface, indicating uniform distribution of Mo_3_S_7_ nanoclusters without notable aggregation (Fig. 2b).

To elucidate the chemical states of the constituent elements, we conducted X-ray photoelectron spectroscopy (XPS) analysis. As shown in Fig. 2c, the Mo 3*d* spectrum of [Mo_3_S_7_Br_6_]^2−^ exhibits characteristic Mo (IV) doublets at 229.8 eV (Mo 3*d*_5/2_) and 232.9 eV (Mo 3*d*_3/2_)^23^. Upon immobilization onto Ti_3_C_2_T_*x*_, both peaks shift toward higher binding energies, centered at 230.3 and 233.5 eV, indicating a reduction in electron density on Mo atoms. This shift is attributed to the substitution of Br ligands with more electronegative O ligands from the Ti_3_C_2_T_*x*_ surface. Additionally, the weak Mo 3 *d* peaks at 232.5 and 235.6 eV, corresponding to Mo (VI), suggest minor oxidation of the Mo_3_S_7_ nanoclusters. The S 2*p* peaks of Mo_3_S_7_-Ti_3_C_2_T_*x*_ exhibit a similar upward shift (Supplementary Fig. 4), implying electron depletion on S atoms following coordination. In contrast, the O 1 *s* peak of Mo_3_S_7_-Ti_3_C_2_T_*x*_ shifts to a lower binding energy relative to pristine Ti_3_C_2_T_*x*_, accompanied by the emergence of a new component at 530.5 eV, which is assigned to Mo–O–Ti bonding^24^. This feature reflects electron accumulation on surface O atoms due to coordination with Mo (Fig. 2d). Furthermore, the absence of Br is verified by comparing the Br 3 *d* XPS spectra of [Mo_3_S_7_Br_6_]^2−^ and Mo_3_S_7_-Ti_3_C_2_T_*x*_ (Fig. 2e). Collectively, these spectral results provide compelling evidence for directional electron transfer from Mo_3_S_7_ nanoclusters to Ti_3_C_2_T_*x*_, indicative of strong interfacial electronic coupling and partial charge redistribution.

X-ray absorption spectroscopy (XAS) was next employed to assess the local bonding environment at the Mo_3_S_7_-Ti_3_C_2_T_*x*_ interface. Mo K-edge X-ray absorption near-edge structure (XANES, Supplementary Fig. 5) analysis reveals a positive shift in the absorption edge for Mo_3_S_7_-Ti_3_C_2_T_*x*_ (20,007.8 eV) compared to [Mo_3_S_7_Br_6_]^2−^ (20,007.1 eV), indicating an increased Mo valence state in agreement with the Mo 3 d XPS results. Further structural insights were obtained by Fourier-transform extended X-ray absorption fine structure (FT-EXAFS) analysis (Fig. 2f). Both [Mo_3_S_7_Br_6_]^2−^ and Mo_3_S_7_-Ti_3_C_2_T_*x*_ display a broad fusion peak spanning 1.30–2.24 Å and a characteristic Mo–Mo bond peak at 2.48 Å. FEFF simulations based on the standard [Mo_3_S_7_Br_6_]^2−^ structural model identified five dominant single-scattering paths: three Mo–S, one Mo–Br, and one Mo–Mo (Supplementary Fig. 6)^25^. The broad fusion peak is thus ascribed to the superposition of Mo–S and Mo–Br contributions. In Mo_3_S_7_-Ti_3_C_2_T_*x*_, a distinct shoulder emerges at around 1.5 Å, corresponding to newly formed Mo–O bonds between the Mo_3_S_7_ nanoclusters and the O-rich terminations of Ti_3_C_2_T_*x*_. The authenticity of this feature is validated by its persistence across different *k-*ranges (Supplementary Fig. 7), ruling out the possibility of noise or transformation artifacts. Complementary wavelet transform contour plots further corroborate the structural transformation: in Mo_3_S_7_-Ti_3_C_2_T_*x*_ (Fig. 2g), pronounced signal extensions toward lower *k* (~3 Å^−1^) and *R* (~1.5 Å) values reflect bonding with lighter atoms (*i.e*., O), while attenuation of intensity at *k* ≈ 7 Å^−1^ and *R* ≈ 2.25 Å indicates cleavage of Mo–Br bonds compared to [Mo_3_S_7_Br_6_]^2−^ (Fig. 2h).

### Modulation of nonequilibrium energy relaxation pathways

**Fig. 3:**
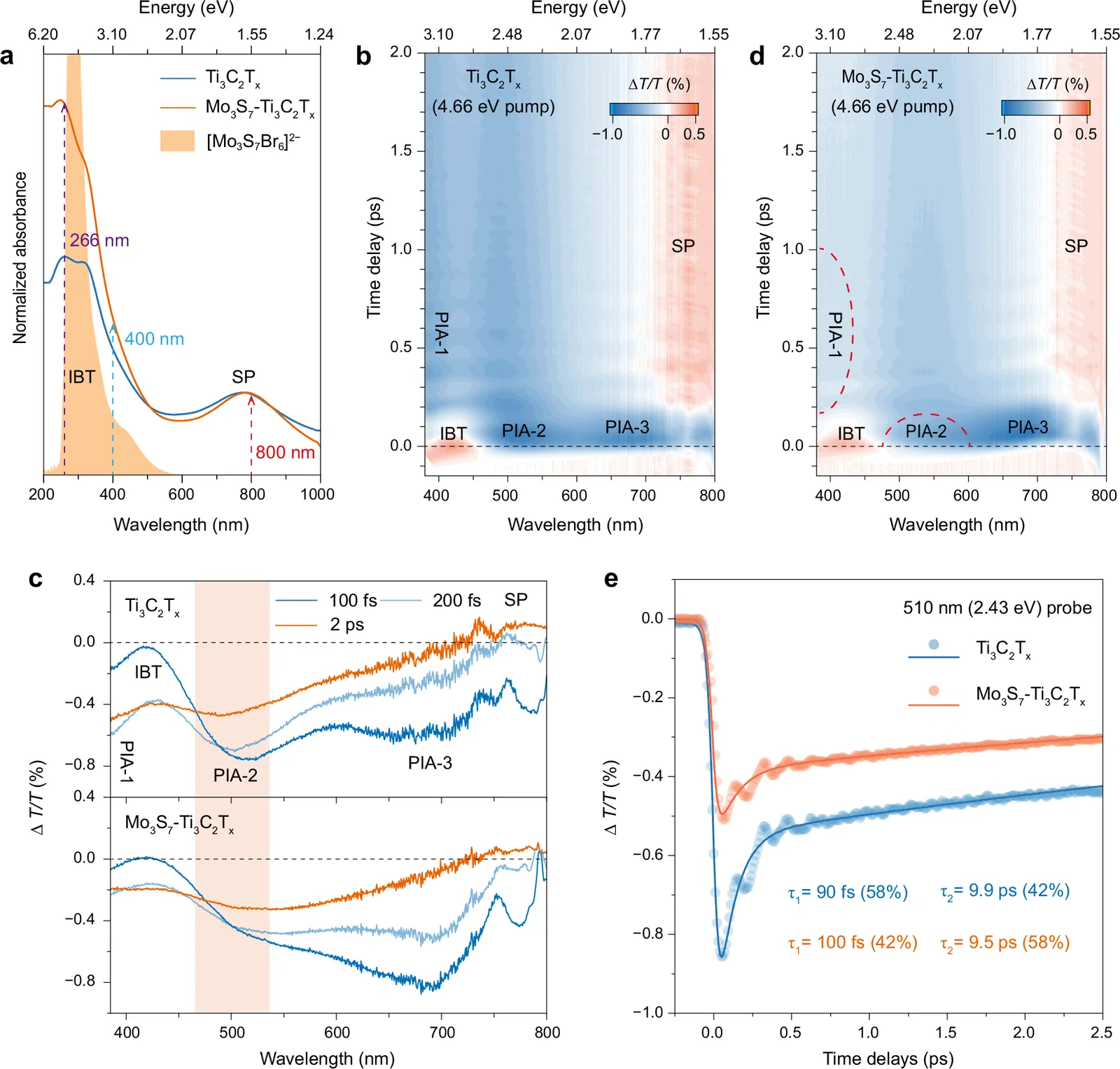
Optical spectra of Ti_3_C_2_T_*x*_ and Mo_3_S_7_-Ti_3_C_2_T_*x*_. **a** Steady-state absorption spectra of Ti_3_C_2_T_*x*_ and Mo_3_S_7_-Ti_3_C_2_T_*x*_ on quartz, and [Mo_3_S_7_Br_6_]^2−^ dispersed in dimethylformamide (0.5 mg/mL). Time-resolved differential transmission (Δ*T/T*) maps of **b** Ti_3_C_2_T_*x*_ and **d** Mo_3_S_7_-Ti_3_C_2_T_*x*_ under 4.66 eV excitation (50 ~ 70 fs-width pulses, excitation fluence 118 µJ/cm^2^ (1.6 × 10^14^ photons cm^−2^)), showing IBT- and SP-related photobleaching features (orange) and PIA signatures (blue). **c** Δ*T/T* spectra of Ti_3_C_2_T_*x*_ and Mo_3_S_7_-Ti_3_C_2_T_*x*_ at selected time delays of 100 fs, 200 fs, and 2 ps, extracted from (**b**) and (**d**), respectively. **e** Kinetic traces of Ti_3_C_2_T_*x*_ and Mo_3_S_7_-Ti_3_C_2_T_*x*_, probed at 510 nm (2.43 eV) under 4.66 eV excitation. Source data are provided as a Source Data file.

The effect of Mo_3_S_7_ nanocluster anchoring on light absorption was first evaluated by comparing the steady-state absorption spectra of Ti_3_C_2_T_*x*_ and Mo_3_S_7_-Ti_3_C_2_T_*x*_, normalized to the surface plasmon resonance (SPR) peak of Ti_3_C_2_T_*x*_ at 780 nm (Fig. 3a). In addition to the prominent SPR feature, Ti_3_C_2_T_*x*_ exhibits IBT-dominated absorption in the UV-Vis region (200–580 nm)^26,27^. Upon incorporation of Mo_3_S_7_ nanoclusters, Mo_3_S_7_-Ti_3_C_2_T_*x*_ presents enhanced absorption starting from ~500 nm and intensifying toward the UV region, closely resembling the characteristic absorption profile of [Mo_3_S_7_Br_6_]^2−^. The absorption contribution of the incorporated Mo_3_S_7_ nanoclusters was approximately evaluated using an optical differential approach, focusing on the spectral region above ~512 nm (2.42 eV) (Supplementary Fig. 8).

To investigate nonequilibrium carrier dynamics, ultrafast TA spectroscopy was employed. The differential transmission (Δ*T/T*) was quantified as Δ*T/T* = (*T*_pump on_ − *T*_pump off_)/*T*_pump off_. Across three different excitation photon energies, namely 1.55 eV (800 nm), 3.10 eV (400 nm), and 4.66 eV (266 nm), as indicated by the dashed arrows in Fig. 3a, Ti_3_C_2_T_*x*_ exhibits consistent Δ*T/T* spectral features (Fig. 3b and Supplementary Fig. 8). Representative results under 4.66 eV (266 nm) excitation reveal a differential transmission map characterized by two distinct photobleaching signals (Δ*T/T* > 0) at ~424 nm and ~780 nm, corresponding to IBT and SPR absorptions, respectively. Besides, three negative photoinduced absorption (PIA, Δ*T/T* < 0) bands were identified at ~400 nm (PIA-1), ~510 nm (PIA-2), and ~700 nm (PIA-3). To examine their temporal evolution, transient spectra were extracted at selected time delays, i.e., 100 fs, 200 fs, and 2 ps (Fig. 3c). At 100 fs, intense IBT-dominated photobleaching coexists with the three PIA bands. Over the next few hundred fs, PIA-1 and PIA-2 broaden and gradually converge into continuous PIA bands centered near <400 nm and ~500 nm, respectively. In contrast, PIA-3 decays rapidly within ~200 fs, concomitant with the progressive intensification of the SPR-related photobleaching near 780 nm. These spectral dynamics align well with previously reported energy relaxation pathways in Ti_3_C_2_T_*x*_^11–15,28^. Generally, PIA-1 and PIA-2 originate from transient renormalization of intrinsic interband transitions. Their long-lived character reflects efficient coupling between photoexcited carriers and the lattice, indicating that these transitions are strongly influenced by e–ph interactions rather than short-lived electronic scattering alone^27^. The subsequent ns-scale relaxation is governed by heat dissipation across the thermal boundary between Ti_3_C_2_T_*x*_ and its surrounding interface and environment^29,30^. PIA-3 reflects an ultrafast perturbation of the plasmon resonance, which is effectively decoupled from the lattice, arising from enhanced electron***–***electron scattering^27^. As electronic thermalization proceeds, the initial nonthermal broadening relaxes and evolves into a quasi-thermal hot-electron state with reduced oscillator strength, manifested as a bleaching-like band around 780 nm.

The incorporation of Mo_3_S_7_ nanoclusters does not profoundly alter the transient spectral characteristics under excitations at 1.55 eV (800 nm) and 3.10 eV (400 nm) (Supplementary Figs. 10 and 11). In sharp contrast, pronounced spectral changes were observed under 4.66 eV (266 nm) excitation (Fig. 3d). Notably, the PIA-1 (~400 nm) and PIA-2 (~510 nm) bands exhibited pronounced attenuation in Mo_3_S_7_-Ti_3_C_2_T_*x*_, indicative of suppressed pump-induced band renormalization. Similar phenomena, where minimal surface functionalization substantially influences the electronic and/or lattice dynamics of host materials, have also been reported in related systems, such as Mo_3_S_13_ cluster-functionalized TiO_2_ and Pt-loaded Ti_3_C_2_T_*x*_^13,31^. The dynamics further reveal that this attenuation occurs within the instrument’s temporal resolution (~100 fs, Fig. 3c), suggesting that interfacial coupling in Mo_3_S_7_-Ti_3_C_2_T_*x*_ modifies the early-time carrier relaxation dynamics and may introduce an additional ultrafast dissipation pathway that partially competes with hot carrier thermalization (~125 fs) in Ti_3_C_2_T_*x*_^14^. By limiting the buildup of a hot, broadened electronic distribution, this interfacial interaction weakens the band renormalization. Notably, the Mo_3_S_7_ nanoclusters exhibit long-term stability under 266 nm excitation (Supplementary Fig. 12).

Kinetic analysis of the PIA-2 (510 nm probe) decay reveals a pronounced attenuation of Δ*T/T* amplitude (Fig. 3e). To gain quantitative insights into the interfacial interactions and relaxation dynamics, we performed multi-exponential fitting of the kinetic trace probed at 510 nm using a phenomenological model described by equation (1), where S_e_(*t*) represents the time-resolved electron signal, IRF is the instrument response function, *A* and *B* are pre-exponential amplitudes, and *τ*_1_ and *τ*_2_ correspond to the fast and slow decay time constants, respectively. Ti_3_C_2_T_*x*_ and Mo_3_S_7_-Ti_3_C_2_T_*x*_ exhibit nearly identical time-constants within the experimental error, with *τ*_1_ = 90 fs and 100 fs, and *τ*_2_ = 9.9 ps and 9.5 ps, respectively. We tentatively assign *τ*_1_ and *τ*_2_ to two distinct hot carrier cooling pathways, governed by scattering with optical phonons and acoustic phonons, respectively. The assignment of the faster decay component is further supported by the observation of coherent phonon oscillations, as discussed in detail later. The key distinction between Ti_3_C_2_T_*x*_ and Mo_3_S_7_-Ti_3_C_2_T_*x*_ lies in the relative contributions of these decay components. In Ti_3_C_2_T_*x*_, the fast component accounts for 58% of the total initial signal, whereas this fraction drops to 42% in Mo_3_S_7_-Ti_3_C_2_T_*x*_, accompanied by a corresponding increase in the slower component. The diminished initial Δ*T/T* amplitude in PIA-1 and PIA-2, along with the redistribution of decay amplitudes, further corroborates the existence of an ultrafast relaxation pathway at the Mo_3_S_7_-Ti_3_C_2_T_*x*_ interface. This interfacial process likely competes with the intrinsic hot carrier thermalization in Ti_3_C_2_T_*x*_, allowing for the rapid extraction of energy from the nonthermal electronic system before it dissipates into the phonon bath. As a result, a substantial fraction of photogenerated high-energy carriers is funneled into a potentially longer-lived reservoir, an attribute highly desirable for potential applications in photodetection, photovoltaic, and photocatalysis^32,33^.

1 $${{{{\rm{S}}}}}_{{{{\rm{e}}}}}(t)={{{\rm{IRF}}}}\times [{{{{\rm{Ae}}}}}^{-t/{\tau }_{1}}+{{{{\rm{Be}}}}}^{-t/{\tau }_{2}}]$$

**Fig. 4:**
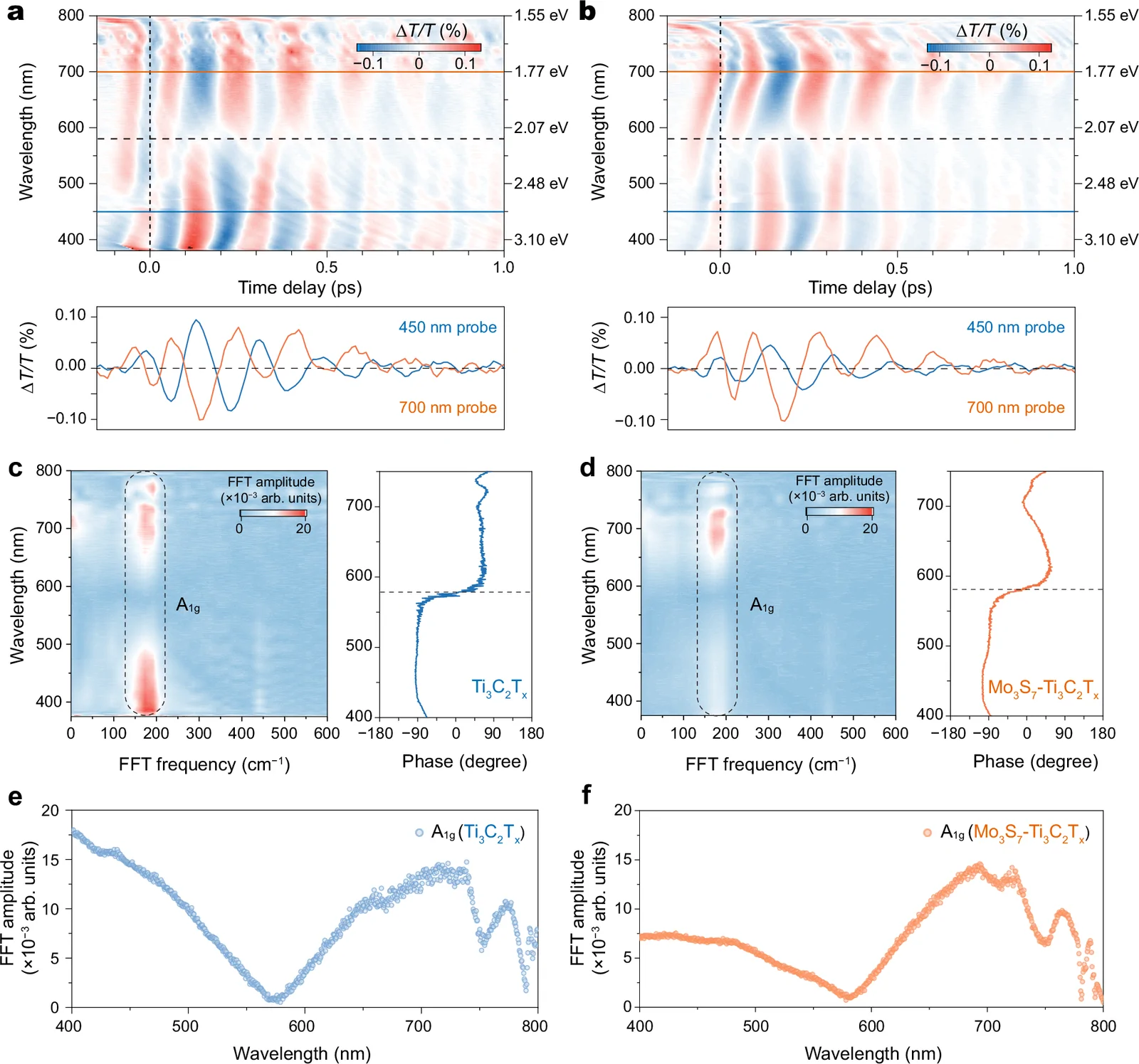
Coherent phonon dynamics in Ti_3_C_2_T_*x*_ and Mo_3_S_7_-Ti_3_C_2_T_*x*_. Oscillatory Δ*T/T* components for **a** Ti_3_C_2_T_*x*_ and **b** Mo_3_S_7_-Ti_3_C_2_T_*x*_ as a function of pump-probe time delay and probe wavelength after incoherent background subtraction. The bottom panels show representative time traces probed at 450 and 700 nm. FFT amplitude maps plotted as a function of oscillation frequency and probe wavelength for **c** Ti_3_C_2_T_*x*_ and **d** Mo_3_S_7_-Ti_3_C_2_T_*x*_. The right panels show the corresponding phase evolution of the coherent A_1g_ phonon mode (selected at 173 cm^−1^). FFT amplitude spectra of **e** Ti_3_C_2_T_*x*_ and **f** Mo_3_S_7_-Ti_3_C_2_T_*x*_ as a function of the probe wavelength at 173 cm^−1^. Source data are provided as a Source Data file.

To gain insights into how ultrafast interfacial energy extraction via covalent anchoring modulates nonequilibrium e–ph interactions in Ti_3_C_2_T_*x*_, coherent vibrational dynamics were extracted by subtracting the incoherent background from the Δ*T*/*T* signal. The resulting maps highlight characteristic oscillations arising from coherent phonon excitations. Notably, a phase flip was observed in both Ti_3_C_2_T_*x*_ (Fig. 4a) and Mo_3_S_7_-Ti_3_C_2_T_*x*_ (Fig. 4b), with an approximately π-phase inversion in the selected oscillatory spectra probed at 450 and 700 nm. Fast Fourier transform (FFT) analysis of the wavelength-dependent coherent modes in Ti_3_C_2_T_*x*_ (Fig. 4c) and Mo_3_S_7_-Ti_3_C_2_T_*x*_ (Fig. 4d) reveals a dominant mode in the 150–200 cm^−1^ range, accompanied by a distinct amplitude dip near 580 nm. This mode corresponds to the previously reported A_1g_ phonon mode of Ti_3_C_2_T_*x*_, which can be coherently excited by the interaction between hot electrons and phonons^11^. An abrupt π-phase shift at selected frequency of 173 cm^−1^ was observed near 580 nm for both Ti_3_C_2_T_*x*_ and Mo_3_S_7_-Ti_3_C_2_T_*x*_, which we attribute to opposing energy-level modulations from two distinct optical transitions: IBT-mediated processes dominate below 580 nm, whereas SPR-related transitions prevail at longer wavelengths (Supplementary Fig. 13). This interplay results in destructive interference between the IBT and SPR excitation pathways in the spectral overlap region, manifested as both a dip in the FFT amplitude and a phase inversion at the crossover wavelength^34,35^.

Notable differences appear on the IBT-dominated side (probe wavelengths <580 nm) when comparing Ti_3_C_2_T_*x*_ and Mo_3_S_7_-Ti_3_C_2_T_*x*_. Specifically, compared to Ti_3_C_2_T_*x*_ (Fig. 4e), the FFT amplitudes of vibrational mode (selected at 173 cm^−1^) extracted from the 2D amplitude maps show pronounced suppression within the 400–580 nm range for Mo_3_S_7_-Ti_3_C_2_T_*x*_ (Fig. 4f and Supplementary Fig. 14). We ascribe this attenuation to efficient interfacial hot carrier harvesting from Ti_3_C_2_T_*x*_ to Mo_3_S_7_. The resulting depletion of high-energy electrons in Ti_3_C_2_T_*x*_ curtails subsequent energy relaxation into the phonon bath, thereby reducing the efficiency of coherent phonon generation. The IBT-dominated region exhibits more pronounced changes due to its higher sensitivity to lattice displacement, whereas the SP-dominated region, governed primarily by collective free-carrier oscillations, is comparatively less affected (Supplementary Fig. 13).

### Photon energy-dependent charge/energy extraction and transport properties

**Fig. 5:**
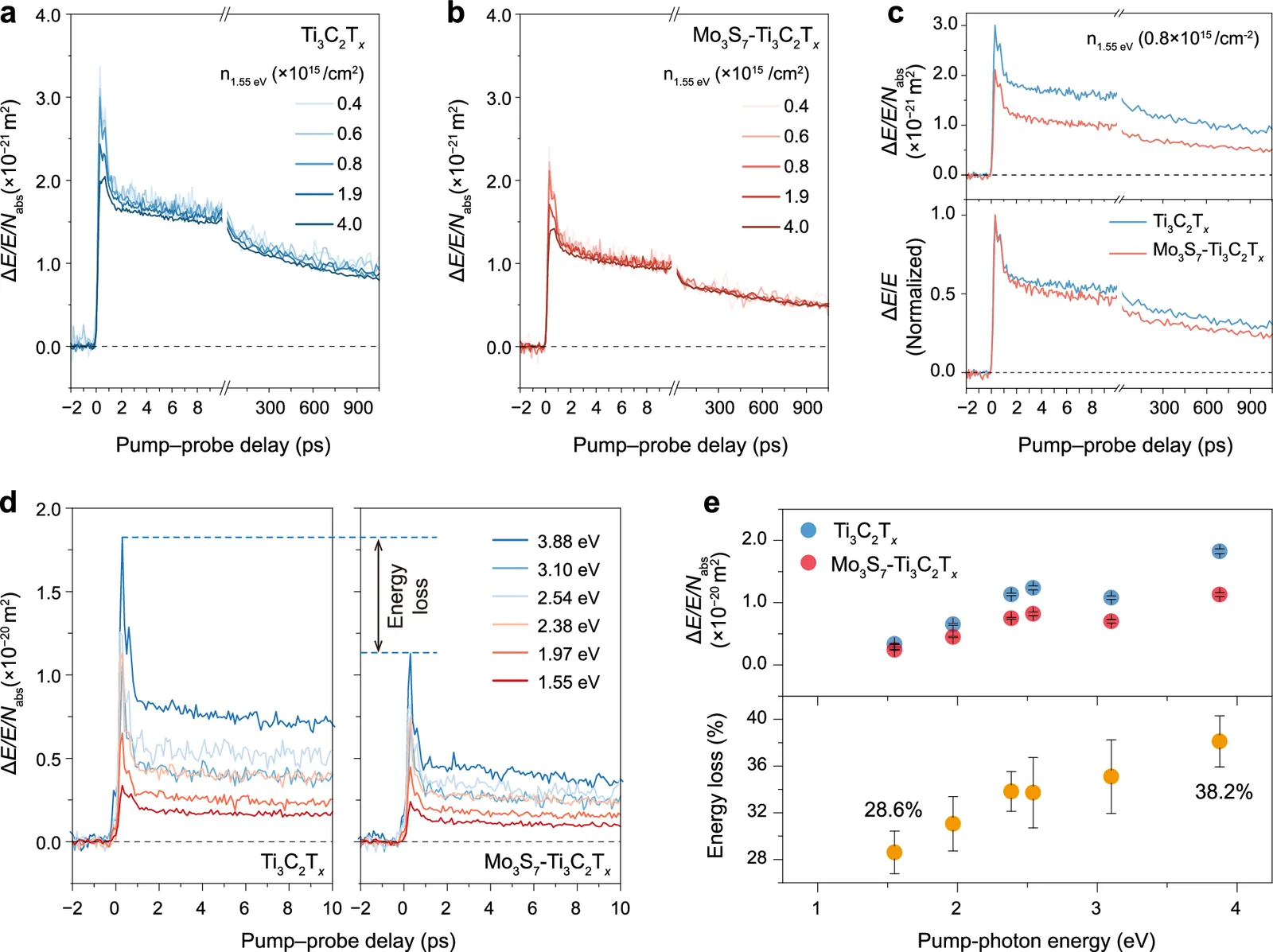
THz photoconductivity dynamics of Ti_3_C_2_T_*x*_ and Mo_3_S_7_-Ti_3_C_2_T_*x*_. Pump fluence-dependent THz photoconductivity dynamics for **a** Ti_3_C_2_T_*x*_ and **b** Mo_3_S_7_-Ti_3_C_2_T_*x*_ under 800 nm (1.55 eV) excitation. **c** Comparison of THz photoconductivity dynamics of Ti_3_C_2_T_*x*_ and Mo_3_S_7_-Ti_3_C_2_T_*x*_ without (top panel) and with (bottom panel) normalization (incident photon density: 0.8 × 10^15 ^cm^−2^). **d** Pump-photon energy-dependent THz photoconductivity dynamics of Ti_3_C_2_T_*x*_ and Mo_3_S_7_-Ti_3_C_2_T_*x*_ (incident photon density: 0.8 × 10^15 ^cm^−2^). **e** Pump photon energy-dependent THz photoconductivity peak values (at 0.3 ps) for Ti_3_C_2_T_*x*_ and Mo_3_S_7_-Ti_3_C_2_T_*x*_ (top panel) and energy loss quantified by considering the relative amplitude reduction (bottom panel). Peak values in the top panel are presented as mean values ± s.e.m. from repeated technical OPTP scans of the same sample. The number of technical scans was *n* = 21, 19, 20, 20, 25 and 21 for Ti_3_C_2_T_*x*_ at 3.88, 3.10, 2.54, 2.38, 1.97 and 1.55 eV, respectively, and *n* = 24, 11, 20, 20, 25 and 23 for Mo_3_S_7_-Ti_3_C_2_T_*x*_. Bottom-panel error bars were obtained by propagation of the corresponding s.e.m. values. Source data are provided as a Source Data file.

The energy relaxation and charge transfer dynamics were further quantified using optical-pump THz-probe (OPTP) spectroscopy, offering a complementary view to the excited-state landscape revealed by TA spectroscopy. In these measurements, a single-cycle THz pulse spanning 0.2 ~ 2.0 THz propagates through the sample, probing the photoconductivity (Δ*σ*_opt_) dynamics by tracking the pump-induced attenuation of the transmitted THz electric field (Δ*E*/*E*~ Δ*σ*_opt_) as a function of the pump–probe delay^36^. To ensure a direct comparison, both Ti_3_C_2_T_*x*_ and Mo_3_S_7_-Ti_3_C_2_T_*x*_ samples were fabricated with comparable thicknesses of ~8.5 Ti_3_C_2_T_*x*_ layers (Supplementary Fig. 15), and their photoconductivity dynamics were normalized to the absorbed-photon density (*N*_abs_). As shown in Fig. 5a, Ti_3_C_2_T_*x*_ exhibits negative THz photoconductivity, i.e., Δ*E*/*E/N*_abs_ > 0, under 1.55 eV (800 nm) excitation across all studied pump fluences (Supplementary Fig. 16a), a hallmark of photoinduced charge carrier heating commonly observed in metallic systems, including MXenes^29,37^. The Δ*E*/*E*/*N*_abs_ dynamics, which reflect the temporal evaluation of carrier temperature, can be broadly divided into three regimes: 1) an ultrafast decay within ~2 ps, whose relative weight diminishes with increasing pump fluence, 2) a slower decay component in the ~10 ps range, and 3) a long-lived component extending into the nanosecond regime. The suppression of the fast component at higher fluences suggests reduced carrier heating efficiency under elevated excitation densities, likely due to absorption saturation (Pauli blocking)^38,39^ or a transition toward phonon-dominated energy relaxation^40^. Another notable feature is the broadening and softening of the photoconductivity peak at high fluences. This phenomenon has also been reported in graphene under elevated pump fluence or reduced Fermi energy, where diminished carrier heating efficiency arises from phase space restrictions or more efficient energy dissipation to phonons^40^.

Incorporating Mo_3_S_7_ nanoclusters preserves the overall dynamical profile but introduces two distinguishable changes (Supplementary Fig. 16b and Fig. 5b). First, Mo_3_S_7_-Ti_3_C_2_T_*x*_ exhibits a reduced Δ*E*/*E*/*N*_abs_ amplitude compared to pristine Ti_3_C_2_T_*x*_ (Fig. 5c). Since the initial amplitude reflects the transient residual electronic heat within the material, this attenuation supports the existence of a rapid (~100 fs, within the instrument resolution limit) nonthermal charge/energy extraction pathway occurring prior to complete electronic thermalization (~125 fs)^14^, consistent with the TA findings. Furthermore, the pump-fluence-dependent THz photoconductivity peak amplitude increases linearly with incident photon density for both samples, consistent with a monotonic rise in electronic temperature (Supplementary Fig. 17). The smaller slope observed for Mo_3_S_7_-Ti_3_C_2_T_*x*_ indicates that the peak electronic temperature (*T*_e_,_peak_) is less sensitive to excitation fluence in the hybrid system, consistent with an interfacial energy or carrier extraction mechanism analogous to that reported in Au/p-GaN heterostructures^41^. Second, the decay of the slower (~10 ps) component is notably accelerated (Fig. 5c). This observation suggests that the presence of coordinatively bonded Mo_3_S_7_ nanoclusters shortens the time required for the electron–phonon system to reach quasi-equilibrium. It is likely due to 1) softening or hybridization of optical and acoustic phonon branches^42^, and 2) the introduction of additional acoustic phonon conduction pathways^43^, which together enhance interfacial thermal conductance and facilitate faster heat dissipation from the Ti_3_C_2_T_*x*_ layers. The nanosecond-scale signal that follows is not affected and can be attributed to heat dissipation across Ti_3_C_2_T_*x*_-Ti_3_C_2_T_*x*_ interfaces or into the substrate/ambient environment^30,44^. The Mo_3_S_7_ loading was varied (Supplementary Fig. 3b), and the corresponding loading-dependent photoconductivity dynamics were investigated. As the Mo_3_S_7_ coverage increases, the Δ*E*/*E*/*N*_abs_ amplitude is further reduced and the heat dissipation dynamics become progressively faster (Supplementary Fig. 18). These trends are consistent with an interaction-driven mechanism, confirming that the observed modulation originates from Mo_3_S_7_-induced interfacial coupling.

As indicated by the TA results, the pump-photon energy plays a critical role in determining the interfacial energy relaxation dynamics in Mo_3_S_7_-Ti_3_C_2_T_*x*_. To further investigate this dependence, we varied the pump-photon energy in the OPTP measurements while maintaining a moderate incident photon density. The initial THz photoconductivity peak amplitude for both Ti_3_C_2_T_*x*_ and Mo_3_S_7_-Ti_3_C_2_T_*x*_ (Fig. 5d), corrected for wavelength-dependent absorption, increases monotonically with pump-photon energy, reflecting more efficient carrier heating at higher pump-photon energies^45^. Meanwhile, the slower (~10 ps) and long-lived (nanosecond-scale) decay components of both Ti_3_C_2_T_*x*_ and Mo_3_S_7_-Ti_3_C_2_T_*x*_ remain nearly invariant across the studied pump-photon energy range (Supplementary Fig. 19). Notably, Mo_3_S_7_-Ti_3_C_2_T_*x*_ constantly exhibits smaller initial Δ*E*/*E*/*N*_abs_ amplitudes than pristine Ti_3_C_2_T_*x*_ across all pump-photon energies, further corroborating the presence of an ultrafast pathway that efficiently extracts energy from hot carriers. To quantify this effect, we extracted the maximum Δ*E*/*E*/*N*_abs_ at ~0.3 ps and plotted it as a function of pump-photon energy (Fig. 5e). While both samples exhibit a similar increasing trend, their amplitude difference becomes more pronounced at higher pump-photon energies. We attribute the amplitude suppression to energy loss mediated by the ultrafast relaxation pathway enabled by Mo_3_S_7_ nanoclusters, following equation (2). We note that the energy loss increases from ~30% at 1.55 eV to ~40% at 3.88 eV. This behavior mirrors the pump-photon energy-dependent attenuation of e–ph interactions observed in the TA measurements. Collectively, these findings demonstrate that Mo_3_S_7_ nanoclusters can efficiently extract energy from hot carriers within sub-100 fs, and that this extraction becomes increasingly effective at higher excitation energies, underscoring their critical role in shaping energy relaxation processes in Ti_3_C_2_T_*x*_.

2 $${{{\rm{Energy}}}} \, {{{\rm{loss}}}}(100\%)=\frac{\varDelta E/E/{N}_{{{{\rm{abs}}}}}({{{{\rm{Ti}}}}}_{3}{{{{\rm{C}}}}}_{2}{{{{\rm{T}}}}}_{x})-\varDelta E/E/{N}_{{{{\rm{abs}}}}}({{{{\rm{Mo}}}}}_{3}{{{{\rm{S}}}}}_{7}-{{{{\rm{Ti}}}}}_{3}{{{{\rm{C}}}}}_{2}{{{{\rm{T}}}}}_{x})}{\varDelta E/E/{N}_{{{{\rm{abs}}}}}({{{{\rm{Ti}}}}}_{3}{{{{\rm{C}}}}}_{2}{{{{\rm{T}}}}}_{x})}\times 100\%$$

**Fig. 6:**
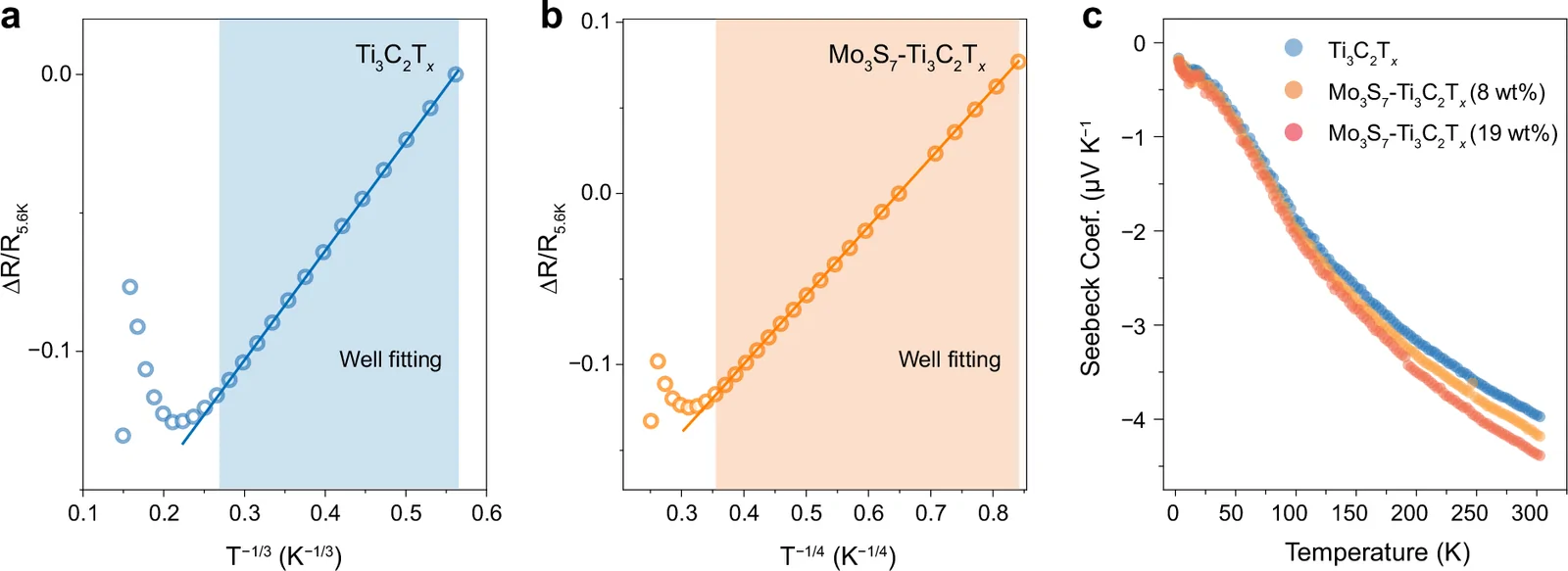
Electric and thermoelectric transport properties of Mo_3_S_7_-Ti_3_C_2_T_*x*_. **a** Normalized resistance vs. T^−1/3^ for Ti_3_C_2_T_*x*_, showing linear behavior below ~100 K characteristic of two-dimensional variable-range hopping. The fitted equation is *y* = −0.22 + 0.39*x*, with R^2^ = 0.9999. **b** Normalized resistance vs. T^−1/4^ for Mo_3_S_7_-Ti_3_C_2_T_*x*_, indicating three-dimensional variable-range hopping. The fitted equation is *y* = −0.26 + 0.40*x*, with R^2^ = 0.9999. **c** Seebeck coefficient of Ti_3_C_2_T_*x*_ and Mo_3_S_7_-Ti_3_C_2_T_*x*_ with different cluster loadings, showing the incremental influence of Mo_3_S_7_ on thermoelectric effect. Source data are provided as a Source Data file.

Steady-state transport measurements were performed to further understand the electronic coupling introduced by Mo_3_S_7_ anchoring (Supplementary Fig. 20). Analysis of the low-temperature transport behavior using variable-range hopping (VRH) models reveals distinct conduction regimes for the two systems. Pristine Ti_3_C_2_T_*x*_ follows a linear dependence in the T^−1/3^ representation (Fig. 6a), characteristic of two-dimensional VRH transport, whereas Mo_3_S_7_-Ti_3_C_2_T_*x*_ exhibits a linear dependence in the T^−1/4^ representation (Fig. 6b), indicating three-dimensional VRH conduction^46^. This dimensional crossover suggests that Mo_3_S_7_ nanoclusters introduce effective interlayer electronic coupling between adjacent Ti_3_C_2_T_*x*_ layers. Hall measurements further show that Mo_3_S_7_ incorporation increases both the carrier concentration and the Hall mobility (Supplementary Fig. 20b). In addition, the Seebeck coefficient remains negative over the entire temperature range, confirming the n-type character of Mo_3_S_7_-Ti_3_C_2_T_*x*_, while the magnitude increases obviously with Mo_3_S_7_ loading (Fig. 6c). This trend suggests a modification of the electronic structure near the Fermi level induced by Mo_3_S_7_ anchoring.

### Computational insights

**Fig. 7:**
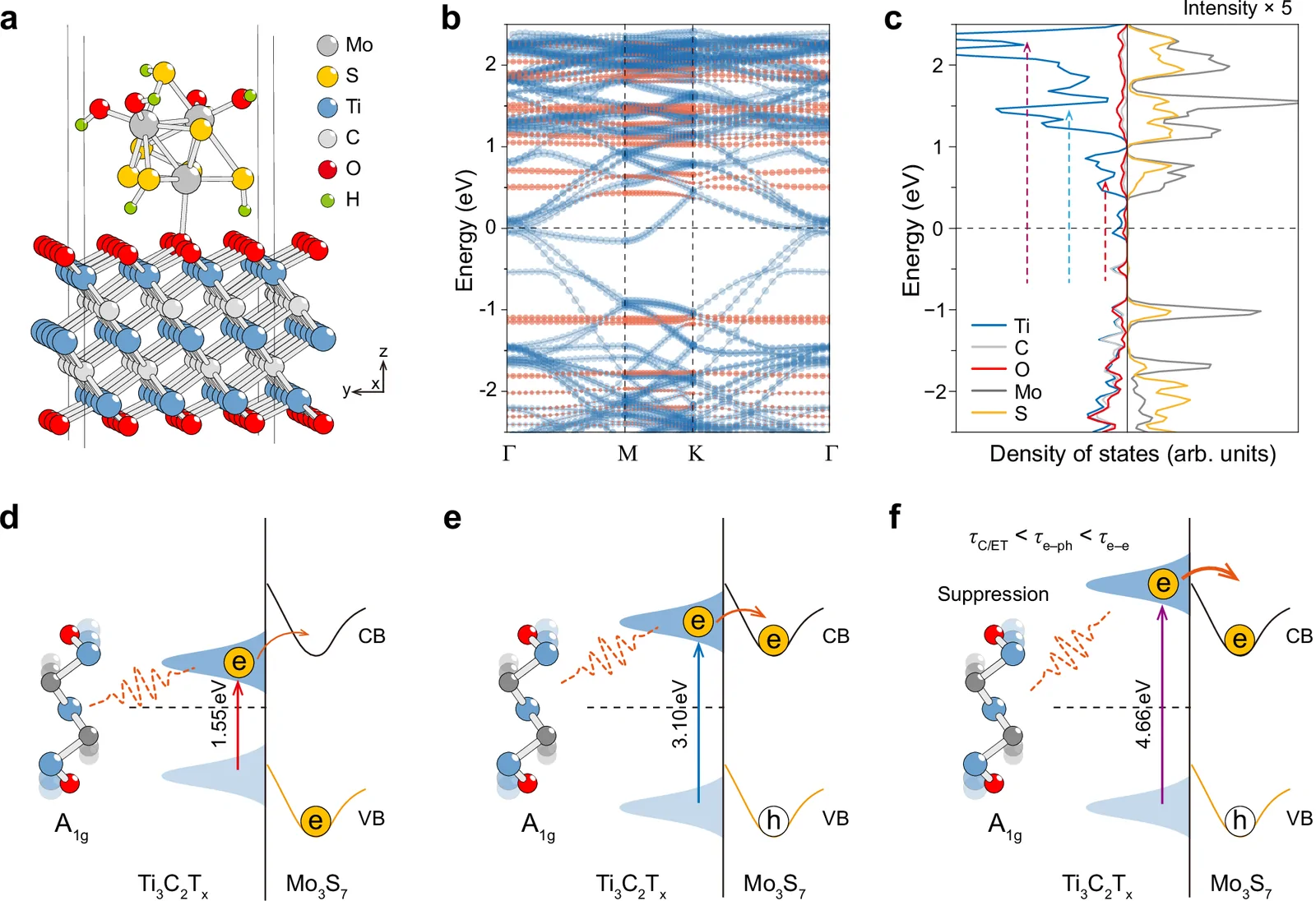
Computational modeling and excitation-dependent charge/energy extraction mechanism. **a** Optimized atomic structure of Mo_3_S_7_-Ti_3_C_2_T_*x*_ based on a 3×3 Ti_3_C_2_O_2_ supercell. **b** Projected electronic band structure of Mo_3_S_7_-Ti_3_C_2_T_*x*_, where orange and blue dots represent the contributions of Mo_3_S_7_ and Ti_3_C_2_T_*x*_, respectively. **c** Projected density of states (DOS) of Mo_3_S_7_-Ti_3_C_2_T_*x*_. Schematic illustration of excitation-dependent charge/energy extraction mechanism in Mo_3_S_7_-Ti_3_C_2_T_*x*_ under **d** 1.55 eV excitation, **e** 3.10 eV excitation, and **f** 4.66 eV excitation. Source data are provided as a Source Data file.

The observed energy dependence of charge/energy extraction and the corresponding modulation of e–ph interaction suggest the presence of low-energy nonthermal electrons that lack sufficient energy to overcome the interfacial extraction barrier. To support this hypothesis, density functional theory (DFT) calculations were performed to gain insights into the electronic structure of Mo_3_S_7_-Ti_3_C_2_T_*x*_. Given the complex surface terminations of Ti_3_C_2_T_*x*_ (Supplementary Table 1), a fully oxygen-terminated Ti_3_C_2_ (Ti_3_C_2_O_2_) was selected as a computationally tractable model, based on prior findings that oxygen terminations are the predominant surface species^11,14,22^. A 3 × 3 supercell of Ti_3_C_2_O_2_ was built to accommodate Mo_3_S_7_ nanoclusters (Fig. 7a). The calculated electronic structure shows that Ti_3_C_2_O_2_ retains metallic character, whereas Mo_3_S_7_ nanoclusters exhibit semiconducting behavior (Fig. 7b). The projected density of states (DOS) for Ti_3_C_2_O_2_ and Mo_3_S_7_ is shown in Fig. 7c, indicating that the unoccupied states above the Fermi level are primarily dominated by Ti 3D orbitals. In metallic systems such as Ti_3_C_2_O_2_, photoexcitation typically involves electronic transitions originating from states near the Fermi level. Meanwhile, higher-energy photons can promote electrons from deeper-lying occupied states to higher-lying unoccupied states.

Based on the calculated electronic structure and our experimental findings, we proposed an energy-dependent modulation mechanism that governs the charge/energy extraction process. Under 1.55 eV excitation, the generated low-energy nonthermal electrons do not possess sufficient excess energy to overcome the offset to the conduction band of Mo_3_S_7_, thereby limiting the efficiency of charge/energy extraction (Fig. 7d). At 3.10 eV excitation, Ti_3_C_2_T_*x*_ and Mo_3_S_7_ are photoexcited simultaneously. In this scenario, the conduction band states of Mo_3_S_7_ become partially occupied by its own photoexcited electrons, resulting in Pauli blocking^47,48^ and/or state-filling effects^49^ that inhibit further charge/energy extraction from Ti_3_C_2_T_*x*_ (Fig. 7e). When the pump photon energy increases to 4.66 eV, two key factors act to alleviate these limitations (Fig. 7f): 1) photoexcited electrons in Mo_3_S_7_ are distributed into higher-energy conduction states, leaving lower-energy states less populated than under 3.10 eV excitation; and 2) electrons in Ti_3_C_2_T_*x*_ are excited to high-energy states, gaining sufficient energy to overcome the interfacial extraction barrier while reducing the influence of state-filling or screening by electrons already present in Mo_3_S_7_. Given the ultrafast charge/energy extraction timescale in Ti_3_C_2_T_*x*_ (~50 fs)^14^, this interfacial extraction likely occurs before Mo_3_S_7_ electrons relax into these lower-energy states.

## Discussion

In summary, we have anchored Mo_3_S_7_ nanoclusters onto Ti_3_C_2_T_*x*_, demonstrating it as an effective strategy to redirect nonthermal electrons and thereby modulate the nonequilibrium e–ph interaction in Ti_3_C_2_T_*x*_. The nanocluster anchoring was achieved via ligand substitution, where cleavage of Mo–Br bonds in [Mo_3_S_7_Br_6_]^2−^ triggered the formation of Mo–O coordination with O-containing terminations of Ti_3_C_2_T_*x*_. TA measurements disclosed a π-phase inversion of the A_1g_ coherent phonon in pristine Ti_3_C_2_T_*x*_, accompanied by destructive interference near 580 nm. This behavior is attributed to phonon-induced modulation of energy levels that oppositely affects IBT and SP absorption. Upon anchoring Mo_3_S_7_ nanocluster, we identified an ultrafast (<100 fs) charge/energy extraction process, accompanied by a probe energy-dependent attenuation of the e–ph interaction, appearing under photoexcitation at 4.66 eV. This extraction led to a suppression of the A_1g_ coherent phonon amplitude by up to ~60 % relative to the pristine Ti_3_C_2_T_*x*_. OPTP analysis further corroborated this ultrafast extraction process and further elucidated the dependence on pump photon energy: higher-energy excitation facilitated more efficient carrier extraction, consistent with improved energetic alignment between hot electron distributions in Ti_3_C_2_T_*x*_ and the Mo_3_S_7_ conduction states. In contrast, lower-energy electrons experienced a potential barrier, reducing their extraction efficiency. These time-resolved spectroscopic measurements collectively demonstrate a strong correlation between carrier extraction efficiency and the suppression of e–ph interactions. Additionally, they reveal how Mo_3_S_7_ nanoclusters could accelerate faster lattice cooling, likely by promoting optical–acoustic phonon mixing and enhancing cross-plane thermal transport. Overall, our findings demonstrate a versatile approach for manipulating ultrafast energy flow in 2D conductors, such as MXenes, by redirecting nonthermal carriers before they thermalize. This femtosecond-scale modulation of nonequilibrium e–ph interaction offers a promising platform for adaptive photonic, thermoelectric, and optoelectronic devices that require dynamic control over carrier–phonon dynamics.

## Methods

### Chemicals

Ti_3_AlC_2_ (Carbon Ukraine), (NH_4_)_6_Mo_7_O_24_ (81–83% as MoO_3_, Alfa Aesar), (NH_4_)_2_S (40–48 wt% in water, Sigma-Aldrich), S powders (99.5%, Thermo Scientific), hydrobromic acid (HBr, 48% in water, Sigma-Aldrich), NEt_4_Br (98%, Thermo Scientific), LiF (≥ 99%, Thermo Scientific), hydrochloric acid (HCl, ≥ 37%, Honeywell Fluka), DMF (98.5%, Fisher Scientific), deionized water (0.055 μS/cm), quartz wafer (thickness = 700 ± 25 µm, MicroChemicals), fused silica (thickness = 1 mm, 10 × 10 mm, PI-KEM).

### Synthesis of (NEt_4_)_2_Mo_3_S_7_Br_6_

The synthesis was conducted using (NH_4_)_2_Mo_3_S_13_ as a precursor following the reported method with slight modifications^20^. 8.4 g of S powder was carefully added to 40 mL of (NH_4_)_2_S solution, forming a vivid orange polysulfide solution. This polysulfide solution was added dropwise into a 10 mL hot aqueous solution containing 2 g of (NH_4_)_6_Mo_7_O_24_. The mixture was stirred for 10 min and then maintained at a temperature of 90 °C for 20 h. Following the completion of the reaction, red needle-like crystals were formed. These crystals were then isolated through filtration and subjected to successive washing steps using water, ethanol, carbon disulfide, and diethyl ether to ensure purity and remove any residual impurities. Subsequently, 3 g of resultant (NH_4_)_2_Mo_3_S_13_ was introduced to 25 mL of HBr solution and subjected to reflux for 3 h. A red mixture was obtained as the reflux process concluded, which was filtered while maintaining its elevated temperature. The filtered solution was then introduced to 3 g of NEt_4_Br. To ensure complete reaction and crystal formation, the resulting mixture was allowed to cool and held at 4 °C for 12 h. The resultant orange crystals were then washed with water serval times.

### Preparation of Ti_3_C_2_T_*x*_

To prepare Ti_3_C_2_T_*x*_, 1 g of Ti_3_AlC_2_ was slowly added to a stirred solution containing 1.6 g of LiF in 20 mL of 9 M HCl. After allowing it to react at 38 °C for 48 h, the mixture was repeatedly washed with deionized water by centrifugation at 1420 g for 5 min until the pH value of the upper liquid was around 6–7. Additionally, the mixture of DMF (80% in volume) and deionized water (20% in volume) was introduced, followed by the sonication for 30 min to accelerate the delamination. The solution was centrifuged at 1680 g for 30 min (repeated for 3 times) to remove the unetched MAX phase and multiple layers of MXene. Finally, the resulting solution was degassed with Ar for 30 min and stored in the refrigerator at 4 °C. Monolayer Ti_3_C_2_T_*x*_ films were prepared through a self-assembly process, as described in our previous work^15^.

### Preparation of Mo_3_S_7_-Ti_3_C_2_T_*x*_

40 mg (NEt_4_)_2_ Mo_3_S_7_Br_6_ was dissolved in 5 mL DMF, which was then introduced dropwise to 10 mL Ti_3_C_2_T_*x*_ solution with stirring. Aggregation of Ti_3_C_2_T_*x*_ was observed after 30 min. The mixture was allowed to react under ambient light for 6 h. The product was washed with DMF and ethanol by centrifugation at 1420 g for 5 min. The resulting dispersion was dripped onto the water surface to form a film, which is subsequently transferred onto appropriate substrates for further characterization. Free-standing thick films were prepared by vacuum-assisted filtration for Seebeck measurements. Notably, thermal treatment of the Ti_3_C_2_T_*x*_ solution (~50 °C) increases the abundance of oxygen terminations, which likely promotes the formation of Mo–O bonds, thereby enhancing the loading of Mo_3_S_7_.

### Characterizations

The morphology of the samples was characterized using transmission electron microscopy, TEM (JEOL Jem F-200C). SEM images and EDS data were acquired using a Zeiss Gemini S4 500 scanning electron microscope equipped with an Oxford XMaxN 150 (150 mm^2^) EDX detector system. PXRD patterns were obtained on an X-ray diffractometer (Aeris Benchtop XRD System) using Cu-K*α* radiation (*λ* = 1.5418 Å) at 40 kV and 15 mA. XPS spectra were collected with Thermo Scientific ESCALAB Xi^+^ X-ray Photoelectron Spectrometer (XPS) Microprobe. Steady-state absorption spectra were measured with a SHIMADZU UV-3600i Plus UV−Vis−NIR spectrophotometer. XAS spectra were collected in transmission geometry with the energy scanning from 19.88 to 21.02 keV at beamline P65 of the Deutsches Elektronen-Synchrotron (DESY). Mo foil was measured simultaneously with the sample for energy calibration. XAS data were processed and analyzed using the Demeter software package^25^. The wavelet-transformed Mo K-edge EXAFS was analyzed using the HAMA code developed by H. Funke and M. Chukalina^50^.

### Transient absorption spectroscopy

Ultrafast TA experiments were performed with two different setups based on Ti: Sapphire lasers (Coherent, Libra 1 and Libra 2) emitting pulses centered at 800 nm, with 1 and 2 kHz repetition rate, 100 fs pulse duration, and a pulse energy of 2 mJ. The 1 kHz laser is used for the pump-probe experiments pumping in the UV and the 2 kHz laser for the experiments pumping at 800 and 400 nm. In both the setup, the laser output was split into two beams to generate the pump and probe pulses. The laser fundamental is used to pump at 800 nm. Pump pulses at 400 nm were generated by frequency doubling the laser fundamental with a BBO plate that provides 90-fs duration pulses. UV pump pulses at 266 nm with sub-50 fs time duration were generated through a two-step process. First, a noncollinear optical parametric amplifier (NOPA), paired with a chirped mirror compressor, produces broadband (80–100 nm FWHM), 10 fs pulses in the visible range, tunable between 500 and 600 nm. These pulses are then frequency-doubled using a thin (0.05 mm) BBO crystal cut at 42°, yielding tunable UV pulses in the 250–300 nm range. The resulting UV beam is filtered with dichroic mirrors and recompressed using a prism pair compressor. On the other hand, the broadband probe beam was generated by white-light-continuum generation focusing the 800-nm beam into a sapphire plate that provides supercontinuum pulses ranging from 380 to 800 nm. Probe’s polarization is adjusted with a *λ*/2 waveplate placed before the supercontinuum generation to form a 90° angle with respect to the pump’s linear polarization. Then, the pump and probe beams were overlapped on the sample. The pump diameter in the focus was 250 μm, and the pump and probe fluences were adjusted with neutral density filters keeping them low to avoid damaging the sample. The pump-probe delay was controlled by means of a mechanical delay stage and pump was modulated with a mechanical chopper at half of the laser repetition rate. Finally, the transient transmission spectra Δ*T*/*T* were collected with an optical multichannel spectrometer after filtering out the pump scattered light with a polarizer parallel to the probe polarization. The transient dynamics were fitted using a Gaussian IRF with a full width at half maximum (FWHM) of 50 fs.

### Optical-pump THz-probe spectroscopy

The OPTP spectrometer was driven by a commercial regenerative amplifier seeded by a mode-locked Ti:sapphire laser (800 nm center wavelength, ≈ 100 fs pulse duration, 1 kHz repetition rate). Pump photons of tunable wavelength were generated via either second-harmonic generation in a BiB_3_O_6_ (BiBO) crystal or a commercial optical parametric amplifier (Light Conversion). After a precisely controlled pump–probe delay, a single-cycle THz probe pulse, produced from the 800 nm beam via optical rectification in a 1 mm-thick ZnTe crystal, was directed collinearly with the pump and transmitted through the sample. The transmitted THz waveform was subsequently recorded by electro-optic sampling, using another 1 mm-thick ZnTe crystal.

### Transport measurements

Electrical and thermoelectric measurements were performed using a Dynacool cryostat (Quantumd Design). Resistance and Hall effect were measured on extended films deposited onto pre-patterned substrates using the electrical transport option (Quantum Design) in the van der Pauw configuration. The Seebeck coefficient was measured on free-standing thick films using the thermal transport option (Quantum Design) with a custom sample holder.

### Computational methods

The first-principles calculations were carried out with the Vienna ab initio simulation package (VASP 5.4.4)^51,52^. The interaction between ions and valence electrons was described using projector augmented wave (PAW) potentials, and the exchange-correlation between electrons was treated using the generalized gradient approximation (GGA) in the Perdew-Burke-Ernzerhof (PBE) form^53^. DFT-D3 method was employed to calculate the van der Waals (vdW) interaction^54^. The parameters of dipole correction were applied for the calculation of slab models. Electronic energies were computed with a tolerance of 1 × 10^−4^ eV and total force of 0.01 eV/Å. A kinetic cutoff energy of 450 eV was adopted. The crystal lattice parameters of MXene bulk are as follows: a = b = 3.03951 Å, c = 19.45795 Å (*α* = *β* = 90^◦^, *γ* = 120^◦^). For the Mo_3_S_7_-Ti_3_C_2_T_*x*_ model, Mo_3_S_7_ nanocluster was loaded on the MXene monolayers along (001) direction to simulate the structure. The theoretical approach was based on the GGA with on-site Coulomb interaction parameter (GGA + U method), in which an effective U-J parameter of 4.2 and 3.5 eV was applied to improve the description of the 3D states of Ti and Mo, respectively^55,56^. Given the structural complexity of the surface Mo_3_S_7_ clusters under realistic conditions, our model places one Mo atom in coordination with Ti_3_C_2_O_2_, while the remaining two Mo atoms are each terminated by two hydroxyl (–OH) groups. To maintain charge neutrality, three additional protons are included in the system. To achieve accurate simulations, a Monkhorst-Pack k-point grid of 7 × 7 × 2 and 3 × 3 × 1 was used for bulk and slab models. Atomic coordinates are provided in Supplementary Data 1.

### Reporting summary

Further information on research design is available in the Nature Portfolio Reporting Summary linked to this article.

## Supplementary information

Supplementary Information

Description of Additional Supplementary File

Supplementary Data 1

Reporting Summary

Transparent Peer Review file

## Source data

Source Data Main Text

Source Data Supplementary

## Data Availability

The data that support the findings of this study are available within the Article and its Supplementary Information. Source data are provided with this paper.
