# Supplementary material for: Modulating nonequilibrium electron–phonon interactions and energy relaxation in MXenes by surface-anchored Mo3S7 nanoclusters
Source: Nat Commun. 2026 Jul 16;17:8829. doi: 10.1038/s41467-026-75614-4 (PMC13500858; doi:10.1038/s41467-026-75614-4)
Supplement: Supplementary file 1 [file 41467_2026_75614_MOESM1_ESM.pdf]

Supplementary Information

Modulating Nonequilibrium Electron–Phonon Interactions and Energy Relaxation in MXenes by Surface-Anchored Mo_3S_7 Nanoclusters

Jiaxu Zhang,[1,†] Rafael Muñoz-Mármol,[2,3,†] Zijie Xiao,[4,†] Dongqi Li,[1†] Shuai Fu,[1,4] Xiaodong Li,[1,5] Juliane Scheiter,[6] Andrea Iudica,[2] Valentino Romano,[2,7] Eva A. A. Pogna,[7] Pengfei Cao,[8] Jingwei Du,[1] Xingyuan Chu,[1] Francesco Scottognella,[2,9] Nicolás Pérez,[6] Kornelius Nielsch,[6,10] Giuseppe Maria Paternò,[2,11,*] Lei Gao,[4,*] Mischa Bonn,[4] Minghao Yu,[1,5,*] Xinliang Feng[1,5,*]

[1] Faculty of Chemistry and Food Chemistry & Center for Advancing Electronics Dresden (cfaed), Technische Universität Dresden, 01062 Dresden, Germany

[2] Department of Physics, Politecnico di Milano, 20133 Milan, Italy

[3] Instituto Universitario de Materiales, University of Alicante, 03690 San Vicente del Raspeig, Spain

[4] Max Planck Institute for Polymer Research, 55128 Mainz, Germany

[5] Max Planck Institute of Microstructure Physics, 06120 Halle (Saale), Germany

[6] Leibniz Institute for Solid State and Materials Research Dresden, Helmholtzstrasse 20, 01069 Dresden, Germany

[7] CNR, Istituto di Fotonica e Nanotecnologie, Piazza Leonardo da Vinci 32, 20133 Milan, Italy

[8] Ernst Ruska-Centre for Microscopy and Spectroscopy with Electrons, Forschungszentrum Jülich, Jülich, Germany

[9] Department of Applied Science and Technology, Politecnico di Torino, Corso Duca degli Abruzzi 24, 10129 Torino, Italy

[10] Institute of Materials Science, Institute of Applied Physics, Technische Universität Dresden, Dresden, Germany.

[11] Center for Nanoscience and Technology, Istituto Italiano di Tecnologia, 20134 Milano, Italy

[†] J. Zhang, R. Muñoz-Mármol, Z. Xiao, & D. Li contributed equally to this work

[*] Corresponding authors: giuseppemaria.paterno@polimi.it, gaol@mpip-mainz.mpg.de, minghao.yu@tu-dresden.de, xinliang.feng@mpi-halle.mpg.de

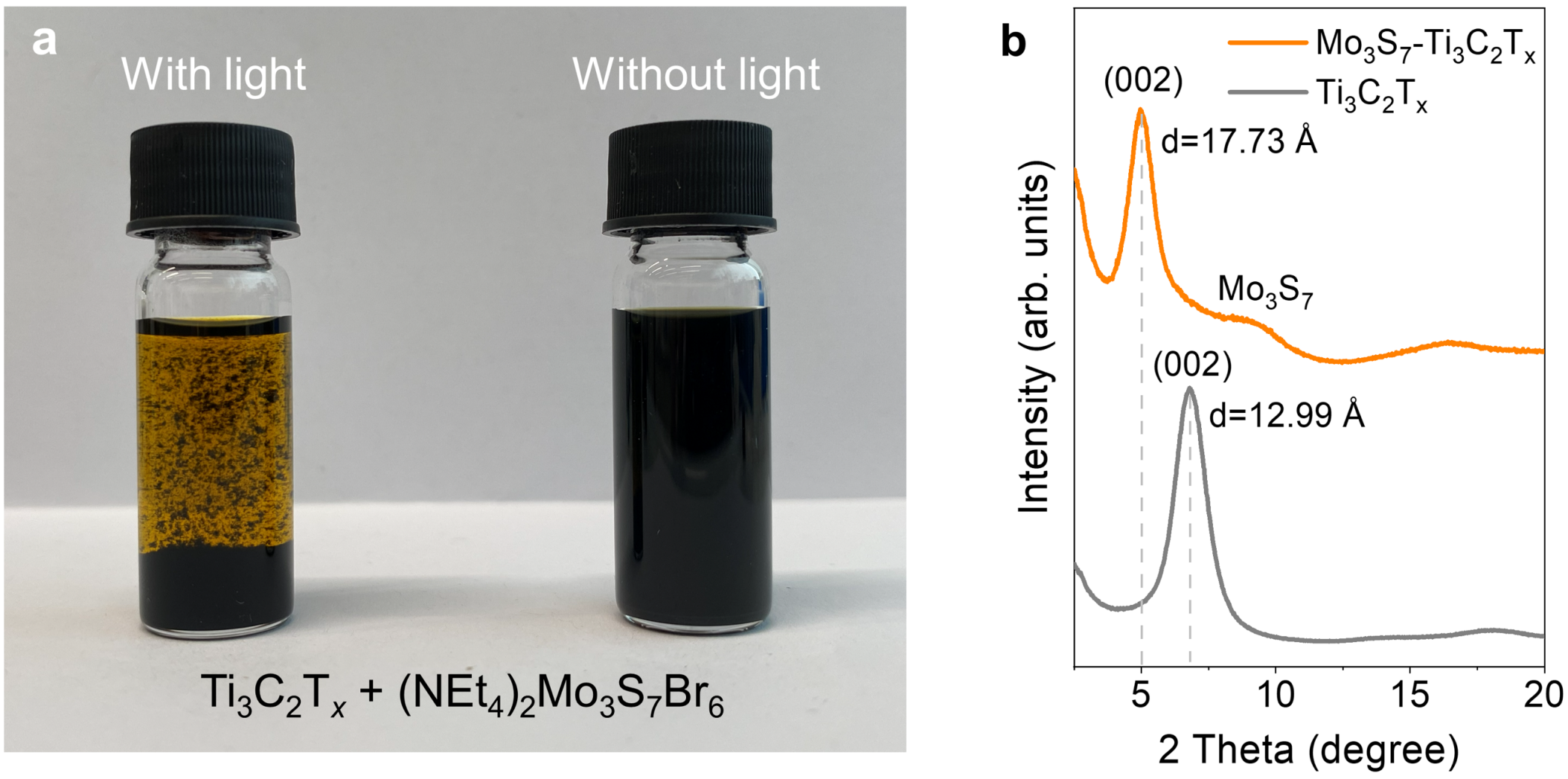

Supplementary Fig. 1 Preparation of Mo_3S_7-$Ti_3C_2T_x$. a Optical images of the mixture of $Ti_3C_2T_x$ and $(NEt_4)_2Mo_3S_7Br_6$ in DMF with and without ambient light exposure. **b** PXRD patterns comparing the pristine $Ti_3C_2T_x$ and Mo_3S_7-$Ti_3C_2T_x$. Source data are provided as a Source Data file.

After nanocluster anchoring, the (002) peak in the X-ray diffraction (XRD) pattern shifted from 2θ = 7° to 5°, signifying a substantial increase in interlayer spacing from approximately 12.99 Å to 17.73 Å. This expansion can be attributed to the existence of Mo_3S_7 nanoclusters between $Ti_3C_2T_x$ layers, which increases the van der Waals gap between the layers.

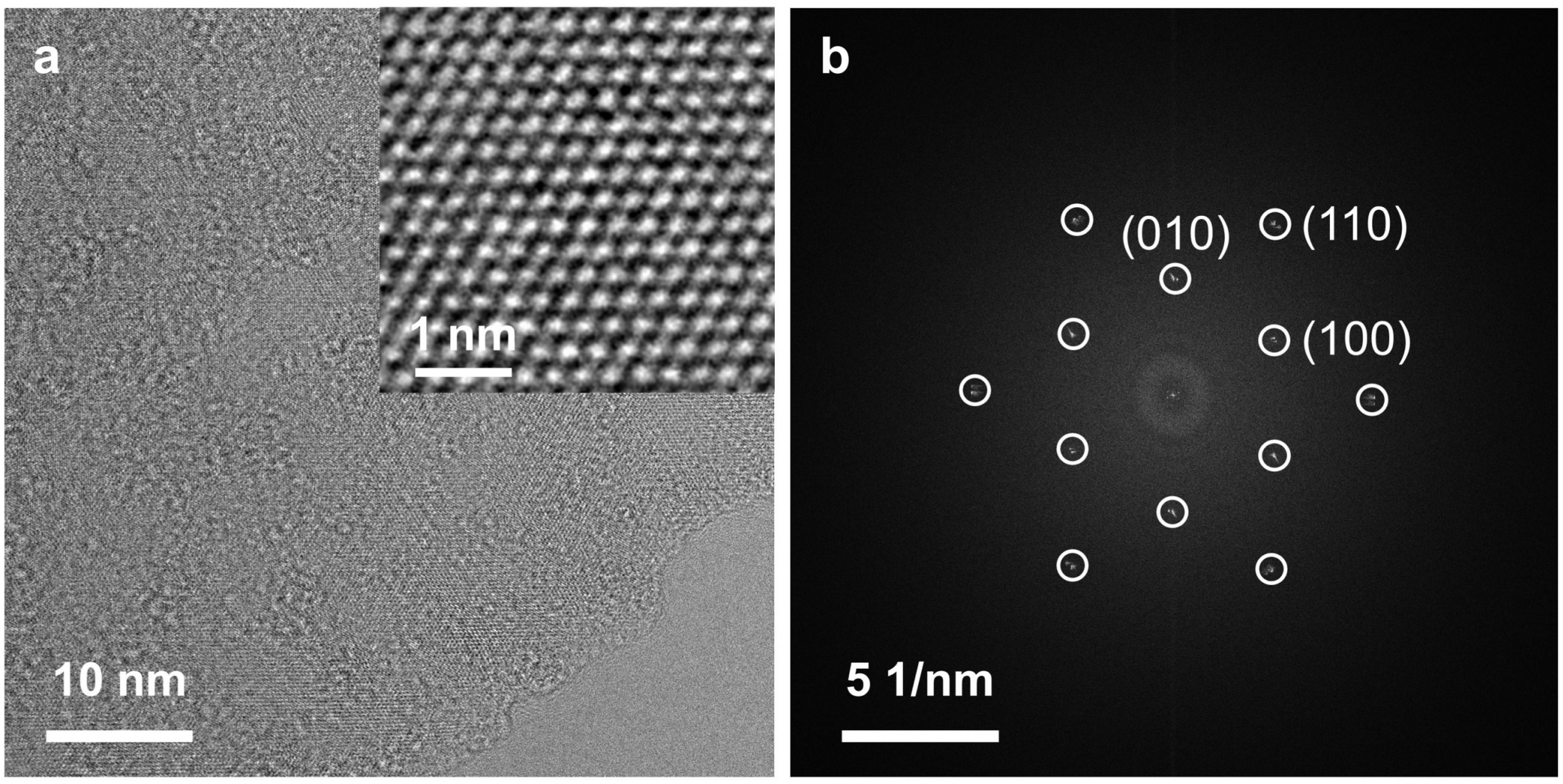

Supplementary Fig. 2 Structural characterizations of Mo_3S_7-$Ti_3C_2T_x$. a High-resolution TEM image and **b** corresponding selected area electron diffraction pattern of Mo_3S_7-$Ti_3C_2T_x$. The TEM imaging experiments were independently repeated twice with similar results.

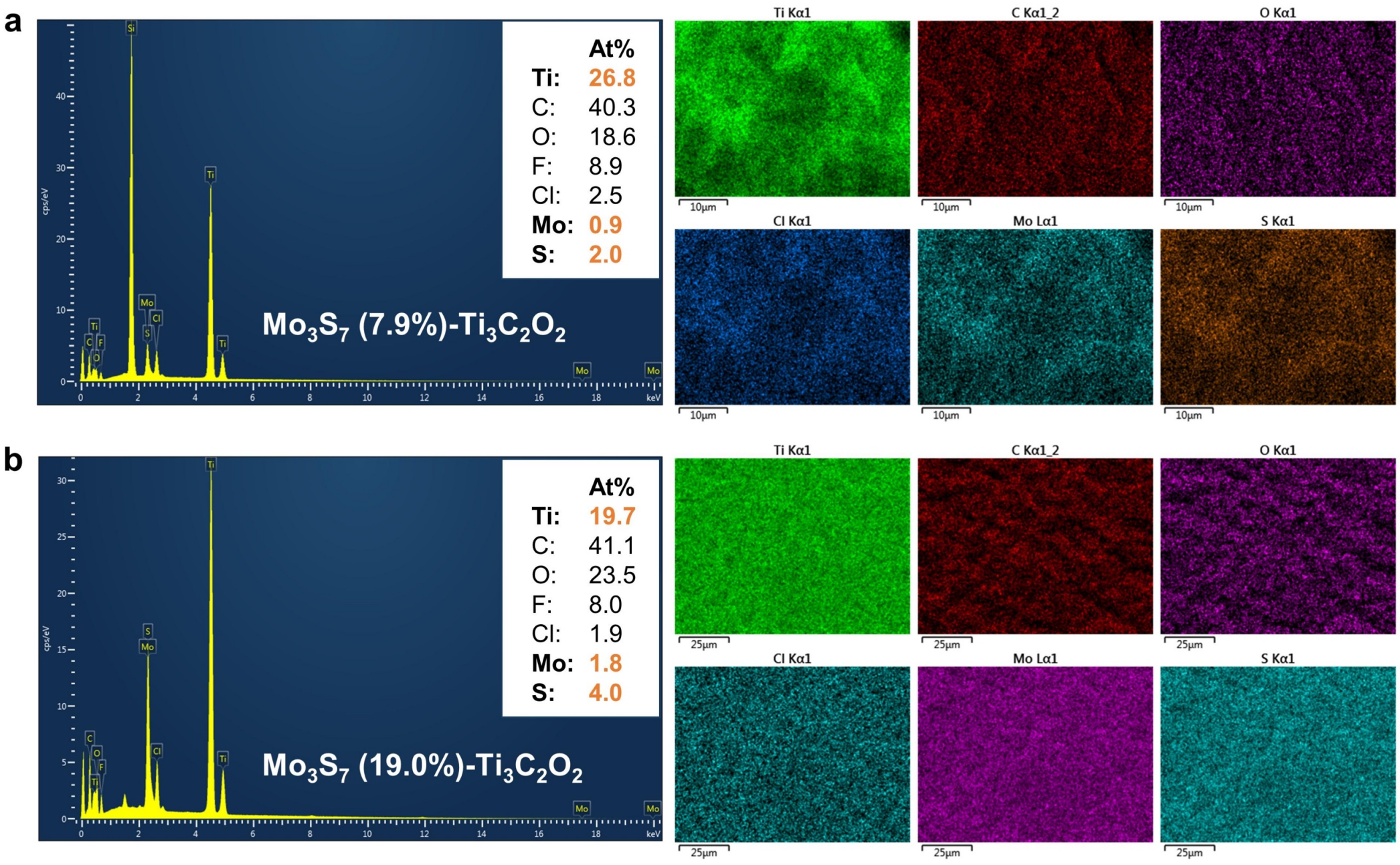

Supplementary Fig. 3 Elemental analysis of Mo_3S_7-$Ti_3C_2T_x$. Elemental analysis of Mo_3S_7-$Ti_3C_2T_x$ synthesized using **a** freshly prepared and **b** thermally treated (~50 °C) $Ti_3C_2T_x$ precursors. The calculated mass fractions of Mo_3S_7 are 7.9% and 19.0%, respectively, assuming a Mo_3S_7-$Ti_3C_2O_2$ composition. Increased oxygen terminations in the thermally treated $Ti_3C_2T_x$ likely promote the formation of Mo-O bonds, thereby enhancing the loading of Mo_3S_7. The SEM-EDS experiments were independently repeated twice with similar results.

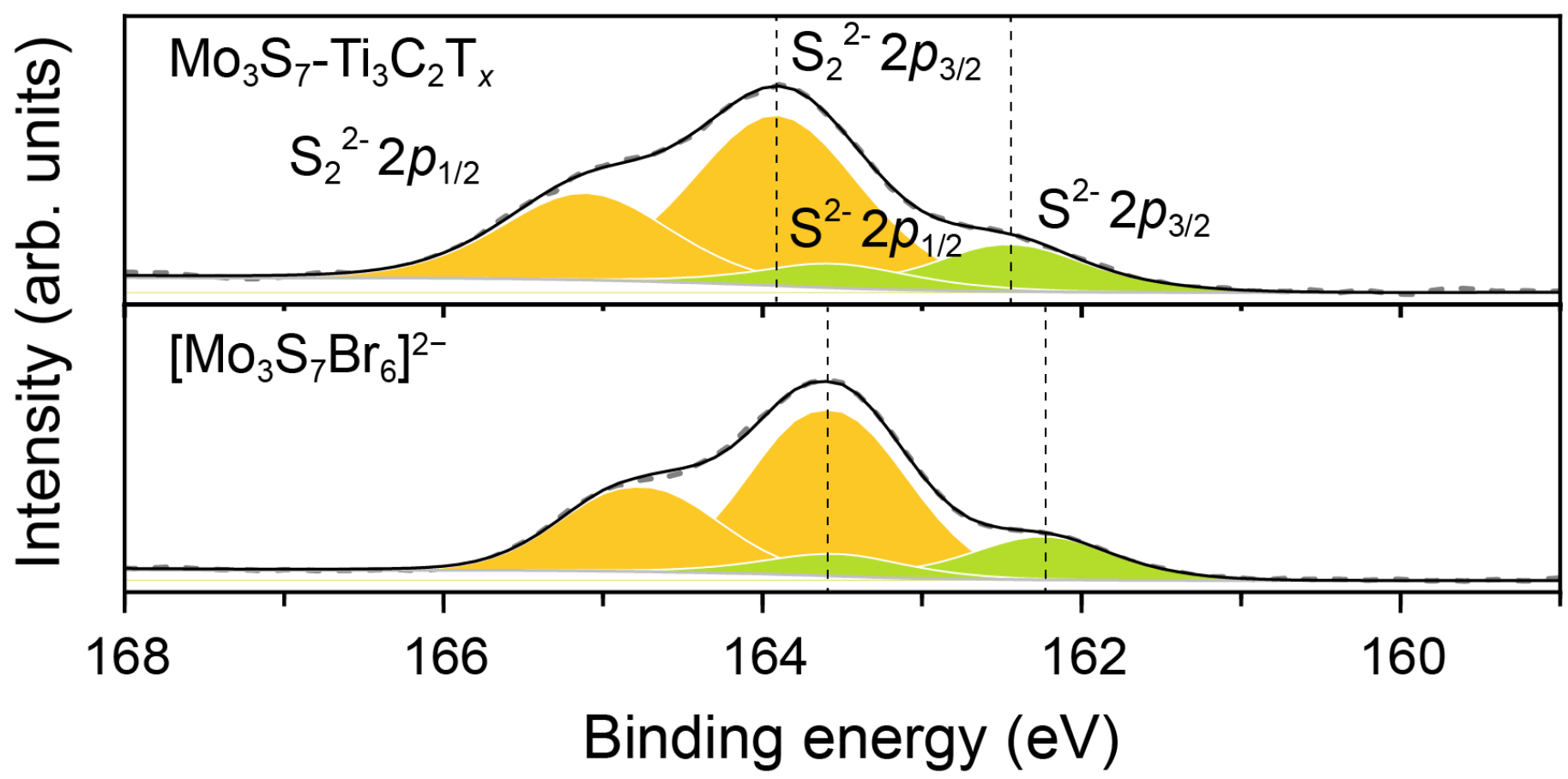

Supplementary Fig. 4 S 2*p* XPS analysis. High-resolution S 2*p* XPS spectra of Mo_3S_7-$Ti_3C_2T_x$ and $[Mo_3S_7Br_6]^{2-}$. Source data are provided as a Source Data file.

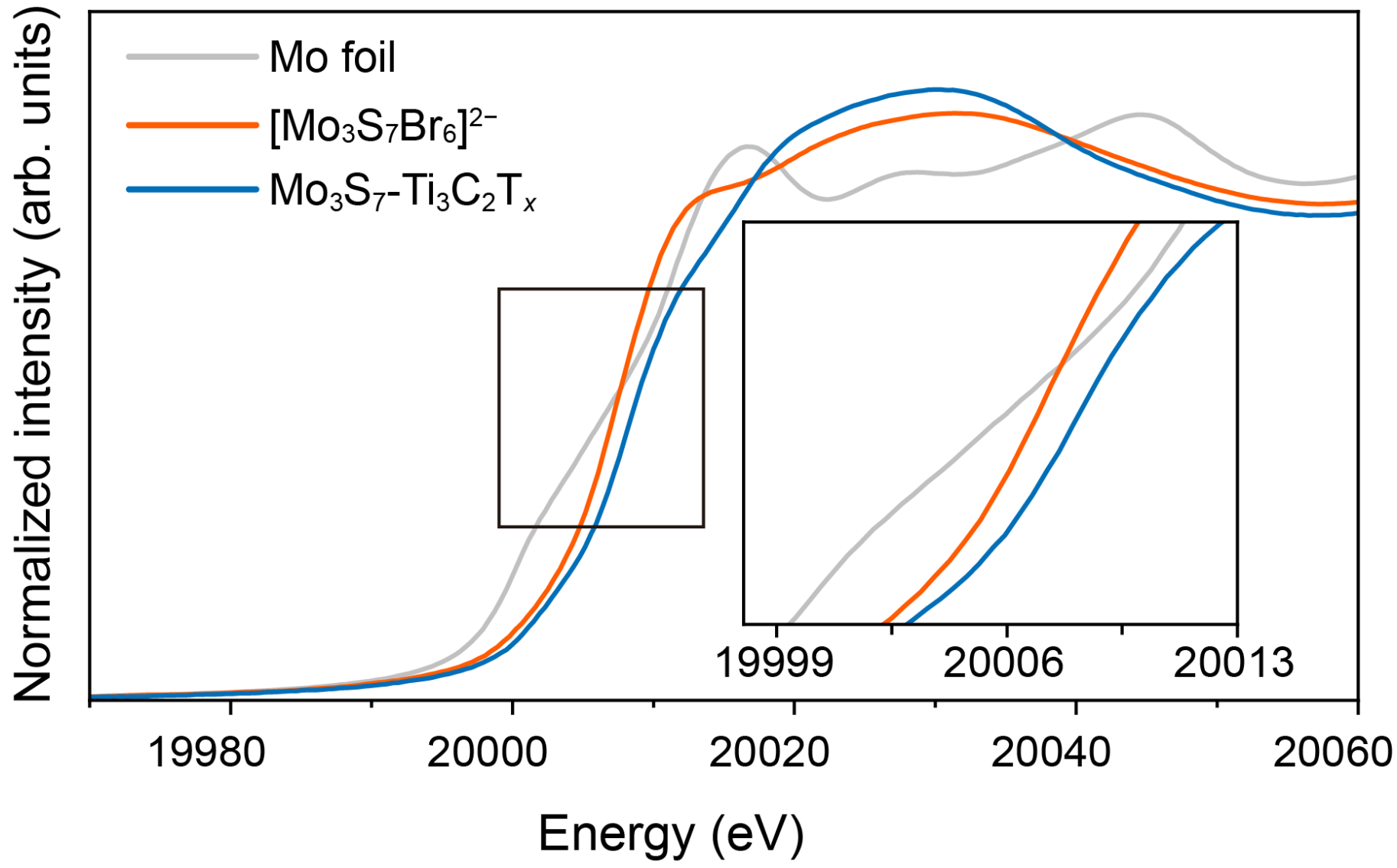

Supplementary Fig. 5 Mo K-edge XANES analysis. Mo K-edge XANES spectra of Mo foil, $[Mo_3S_7Br_6]^{2-}$, and Mo_3S_7-$Ti_3C_2T_x$. Source data are provided as a Source Data file.

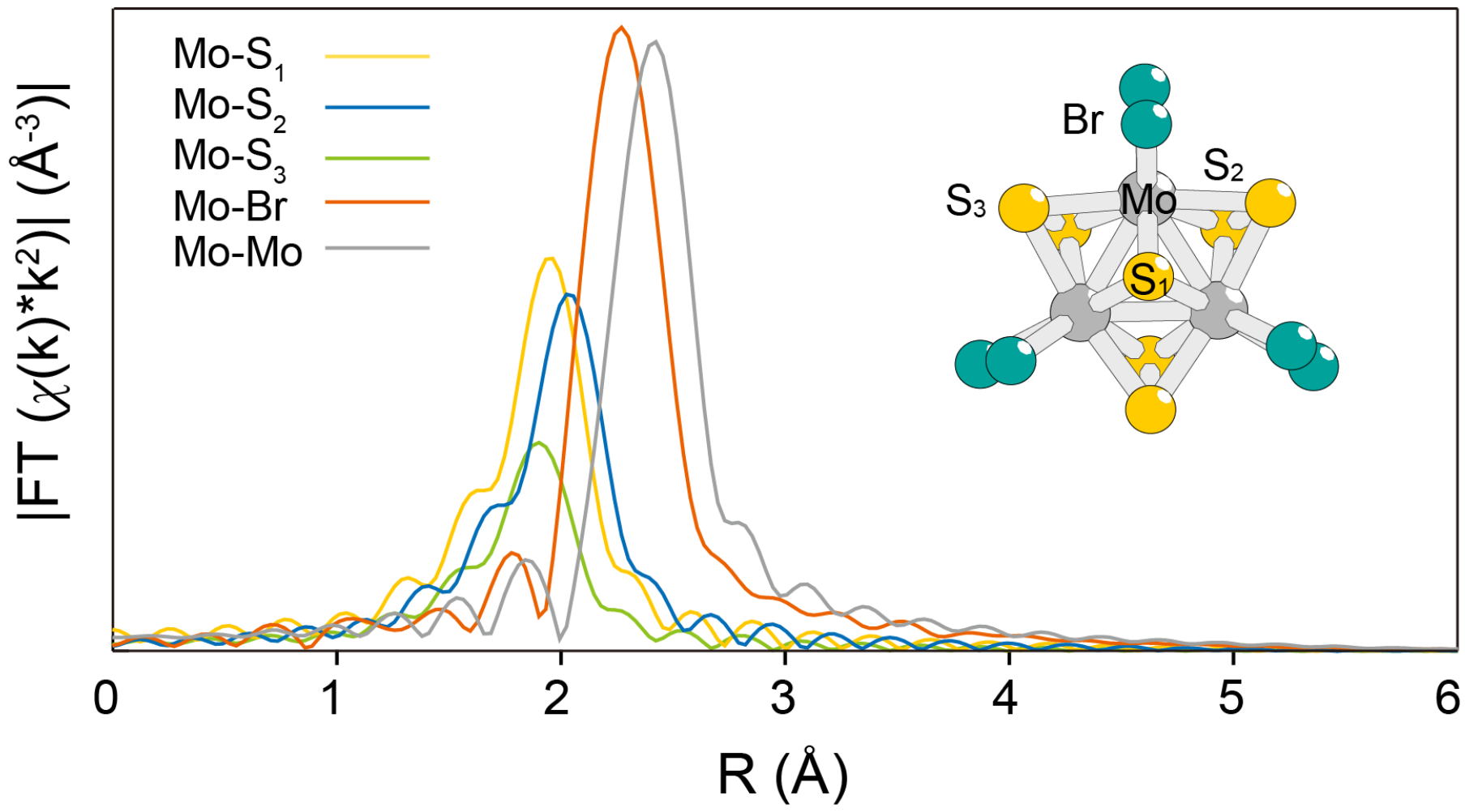

Supplementary Fig. 6 EXAFS scattering-path model of $[Mo_3S_7Br_6]^{2-}$. Simulated scattering paths in $[Mo_3S_7Br_6]^{2-}$, including three Mo–S paths with effective path lengths (R_{eff}) of 2.34 Å, 2.39 Å, and 2.48 Å, one Mo–Br path (R_{eff} = 2.61 Å), and one Mo–Mo path (R_{eff} = 2.74 Å), respectively. The structural model is based on the crystallographic data of $(NEt_4)_2Mo_3S_7Br_6$ (CCDC: 142952). Source data are provided as a Source Data file.

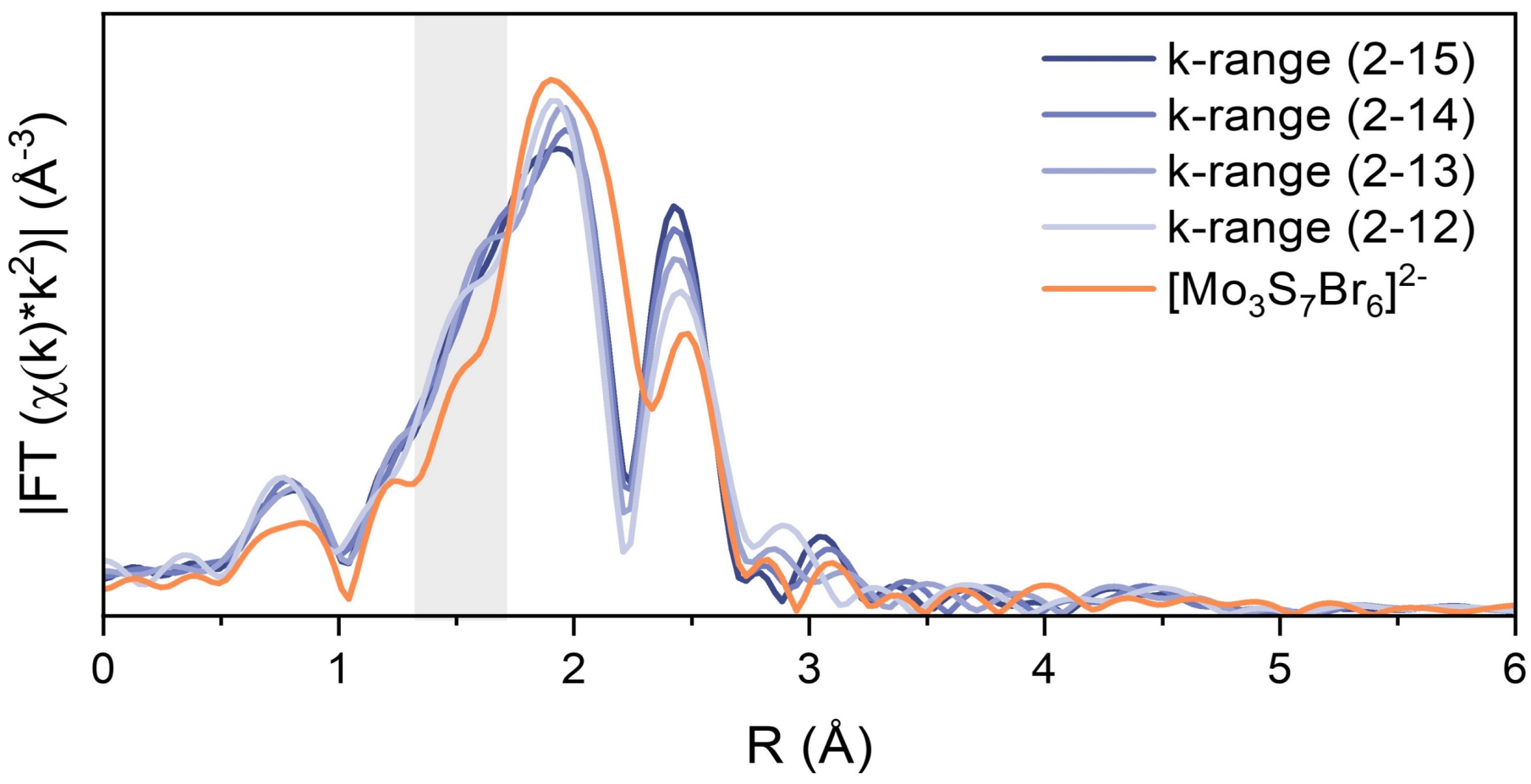

Supplementary Fig. 7 k-range-dependent EXAFS analysis. k^2-weighted Fourier transforms of Mo_3S_7-$Ti_3C_2T_x$ EXAFS with different k-ranges. Source data are provided as a Source Data file.

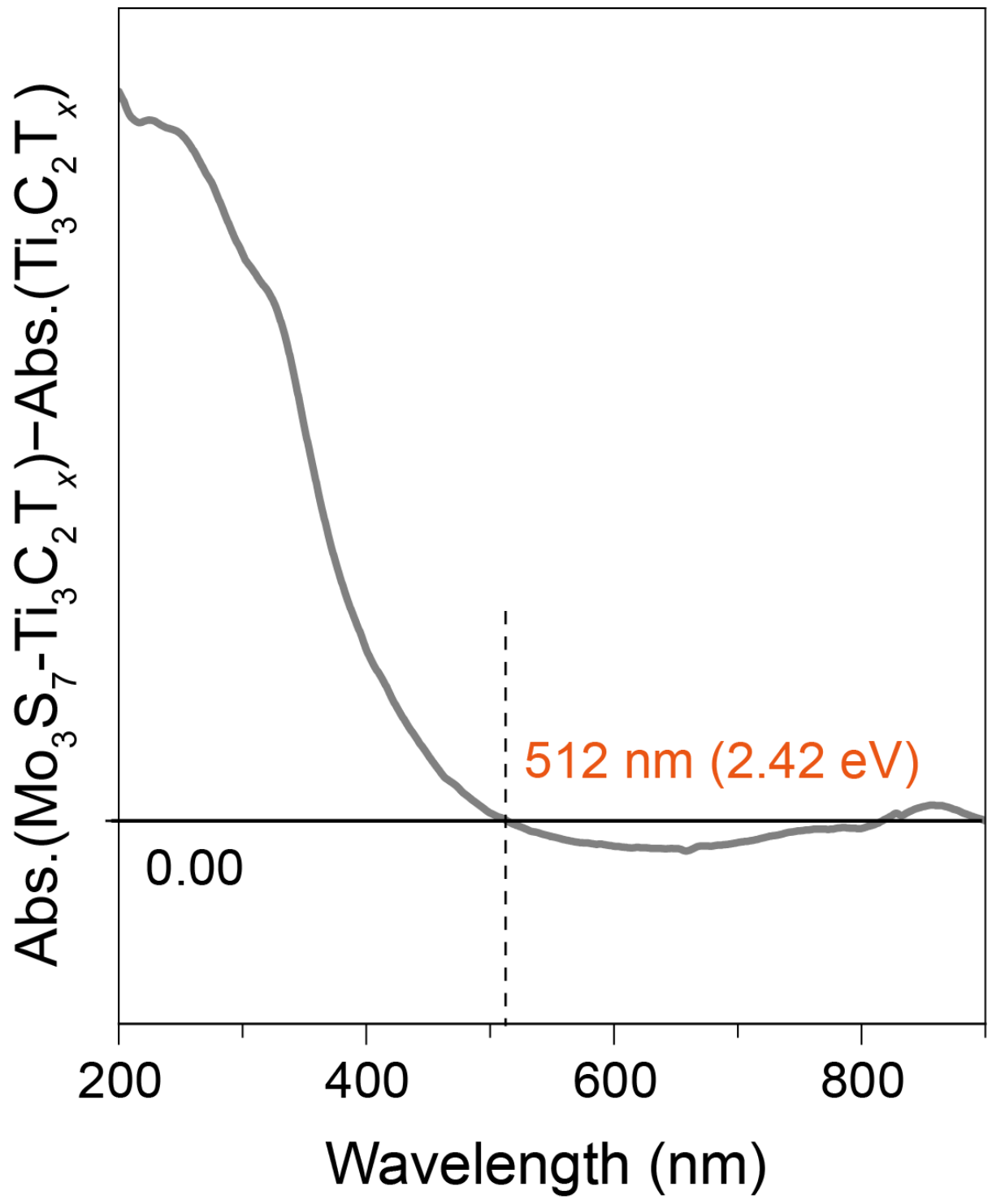

Supplementary Fig. 8 Difference absorption spectrum. Normalized difference absorption spectrum of Mo_3S_7-$Ti_3C_2T_x$ relative to pristine $Ti_3C_2T_x$, obtained after normalization at 900 nm (1.38 eV). Source data are provided as a Source Data file.

Mo_3S_7 is formed *in situ* via bromide dissociation from $Mo_3S_7Br_6$, which prevents direct measurement of its standalone absorption spectrum. Therefore, the calculated bandgap of Mo_3S_7 (~1.5 eV) can be regarded as an approximate reference. To determine a more precise band gap, an optical-differential approach was adopted to estimate the spectral contribution of the Mo_3S_7. Assuming negligible cluster absorption at 900 nm (1.38 eV), the steady-state absorption spectra of $Ti_3C_2T_x$ and Mo_3S_7-$Ti_3C_2T_x$ was normalized at this wavelength. The resulting normalized difference spectrum indicates that additional cluster-related absorption emerges primarily at wavelengths shorter than ~512 nm (~2.42 eV).

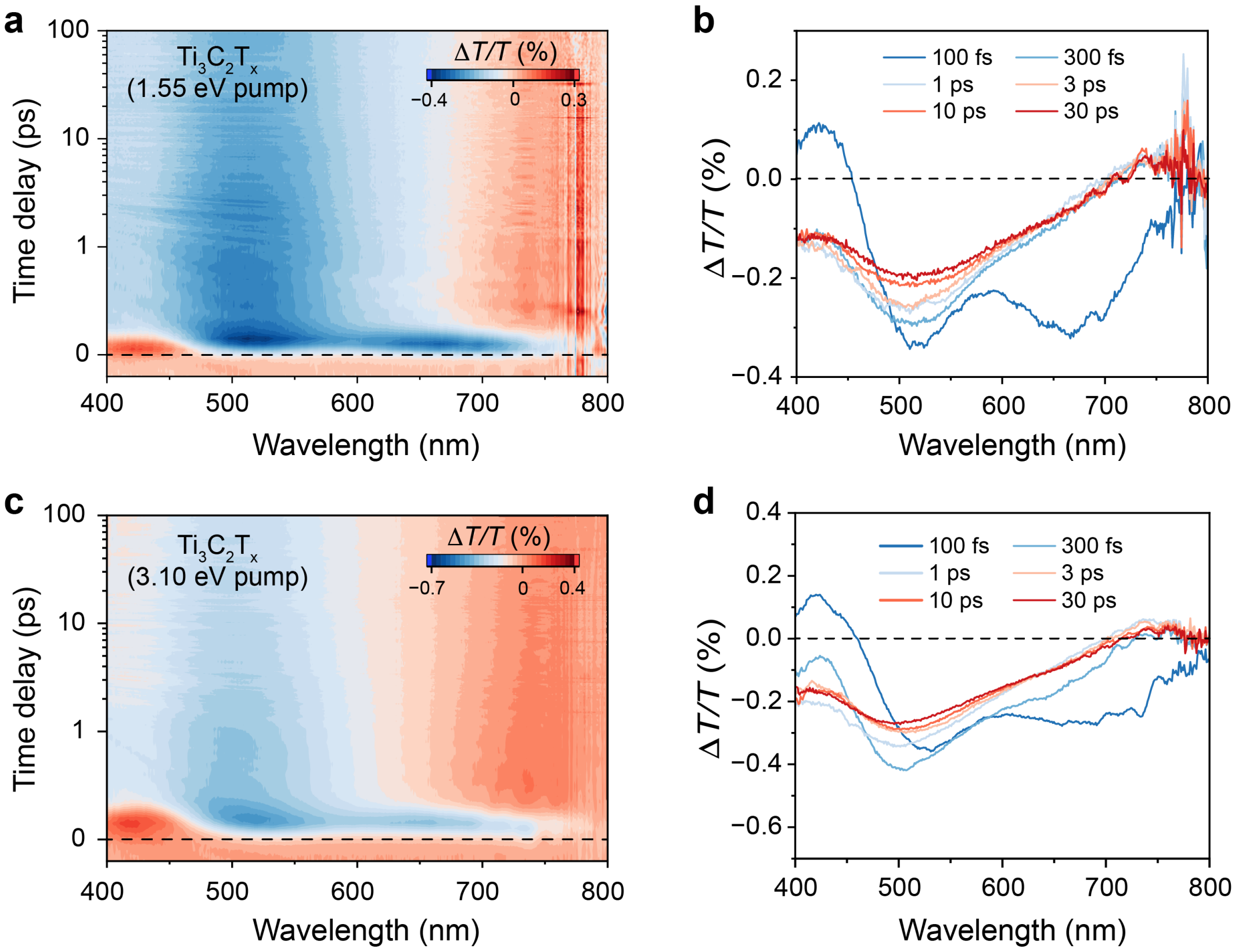

Supplementary Fig. 9 Transient optical responses of $Ti_3C_2T_x$. a Time-resolved differential transmission ($\Delta T/T$) spectra of $Ti_3C_2T_x$ as a function of probe wavelength and delay time under excitation at 1.55 eV with 100 fs pulses and 130 µW fluence. **b** Corresponding $\Delta T/T$ spectra at selected time delays from 100 fs to 30 ps. **c** $\Delta T/T$ spectra of $Ti_3C_2T_x$ as a function of probe wavelength and delay time under excitation at 3.10 eV, and with 90 fs-width pulses and 130 µW fluence. **d** Corresponding $\Delta T/T$ spectra at selected time delays from 100 fs to 30 ps. Source data are provided as a Source Data file.

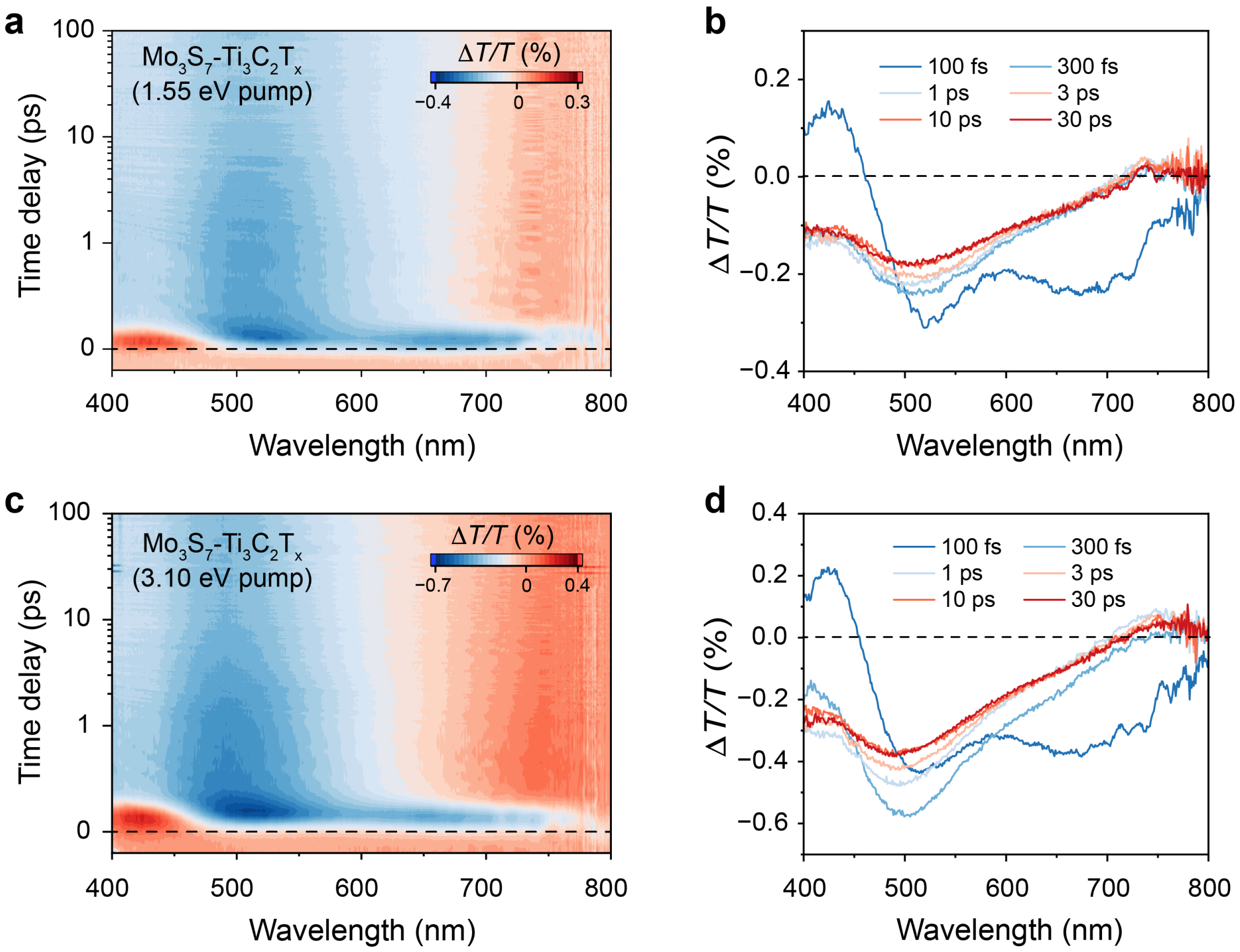

Supplementary Fig. 10 Transient optical responses of Mo_3S_7-$Ti_3C_2T_x$. a Time-resolved differential transmission ($\Delta T/T$) spectra of Mo_3S_7-$Ti_3C_2T_x$ as a function of probe wavelength and delay time under excitation at 1.55 eV with 100 fs-width pulses and 130 µW fluence. **b** Corresponding $\Delta T/T$ spectra at selected time delays from 100 fs to 30 ps. **c** $\Delta T/T$ spectra of Mo_3S_7-$Ti_3C_2T_x$ as a function of probe wavelength and delay time under excitation at 3.10 eV, and with 90 fs-width pulses and 130 µW fluence. **d** Corresponding $\Delta T/T$ spectra at selected time delays from 100 fs to 30 ps. Source data are provided as a Source Data file.

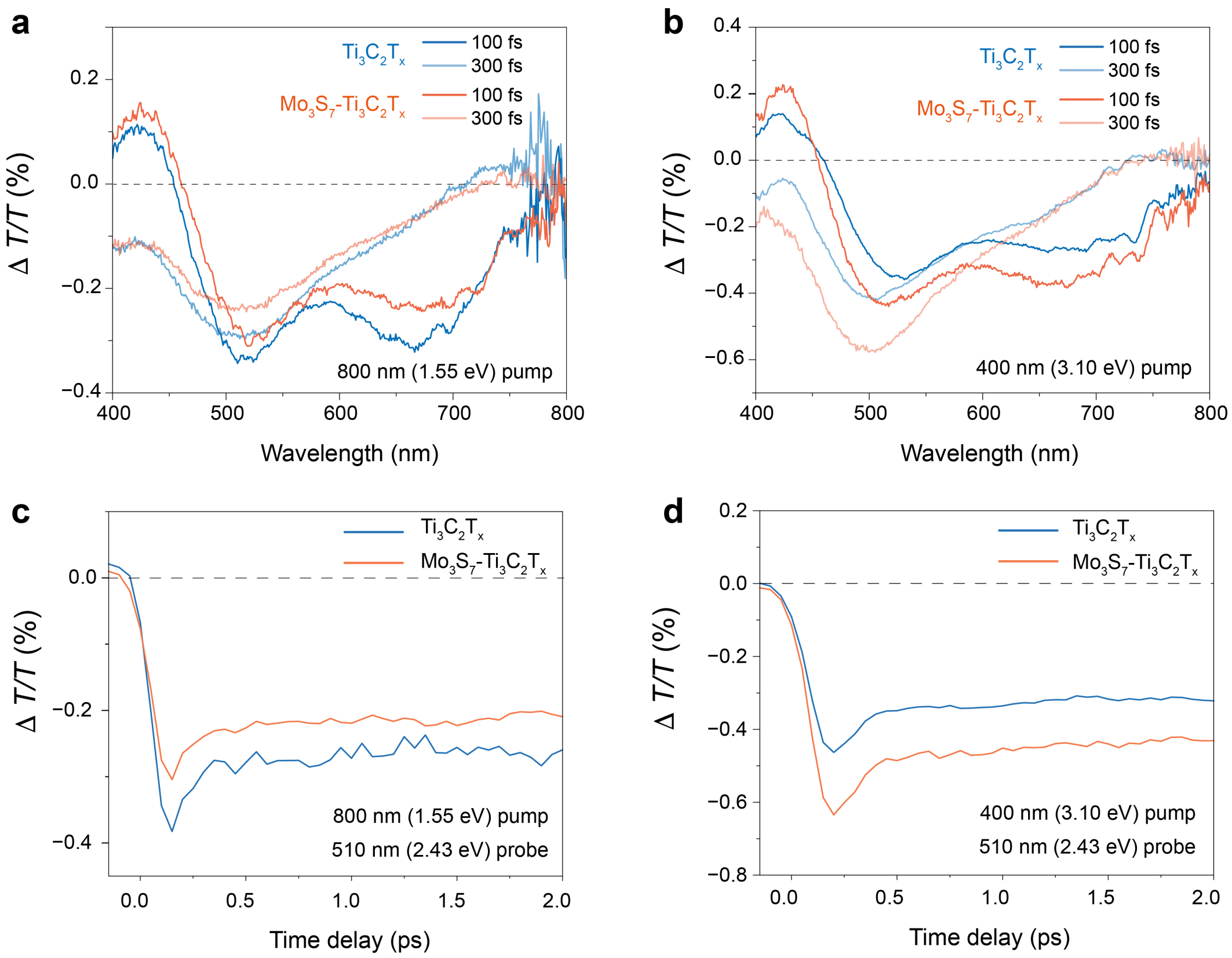

Supplementary Fig. 11 Comparison of transient optical responses of $Ti_3C_2T_x$ and Mo_3S_7-$Ti_3C_2T_x$. Comparison of the $\Delta T/T$ spectra of $Ti_3C_2T_x$ and Mo_3S_7-$Ti_3C_2T_x$ at selected time delays of 100 fs and 300 fs under **a** 1.55 eV and **b** 3.10 eV excitation, respectively. Kinetic traces of $Ti_3C_2T_x$ and Mo_3S_7-$Ti_3C_2T_x$, probed at 510 nm (2.43 eV) under **c** 1.55 eV and **d** 4.66 eV excitation, respectively. Source data are provided as a Source Data file.

Under 800 nm excitation, where Mo_3S_7 is not directly excited, a reduction in PIA intensity is observed that is qualitatively similar to that under 266 nm excitation, although differences in magnitude may arise from variations in interfacial transfer efficiency (Supplementary Fig. 11a and c). In contrast, under 400 nm excitation, where direct excitation of Mo_3S_7 is possible, the PIA-2 and PIA-3 bands exhibit increased intensity, accompanied by a noticeable blue shift of the PIA-2 band (Supplementary Fig. 11b and d). The observed increase in intensity may arise from the contribution of Mo_3S_7, which spectrally overlaps with the PIA-2 band of $Ti_3C_2T_x$. Notably, under 266 nm excitation, the $Ti_3C_2T_x$ contribution is expected to be even stronger than under 400 nm excitation, which likely overwhelms the contribution originating from Mo_3S_7 clusters.

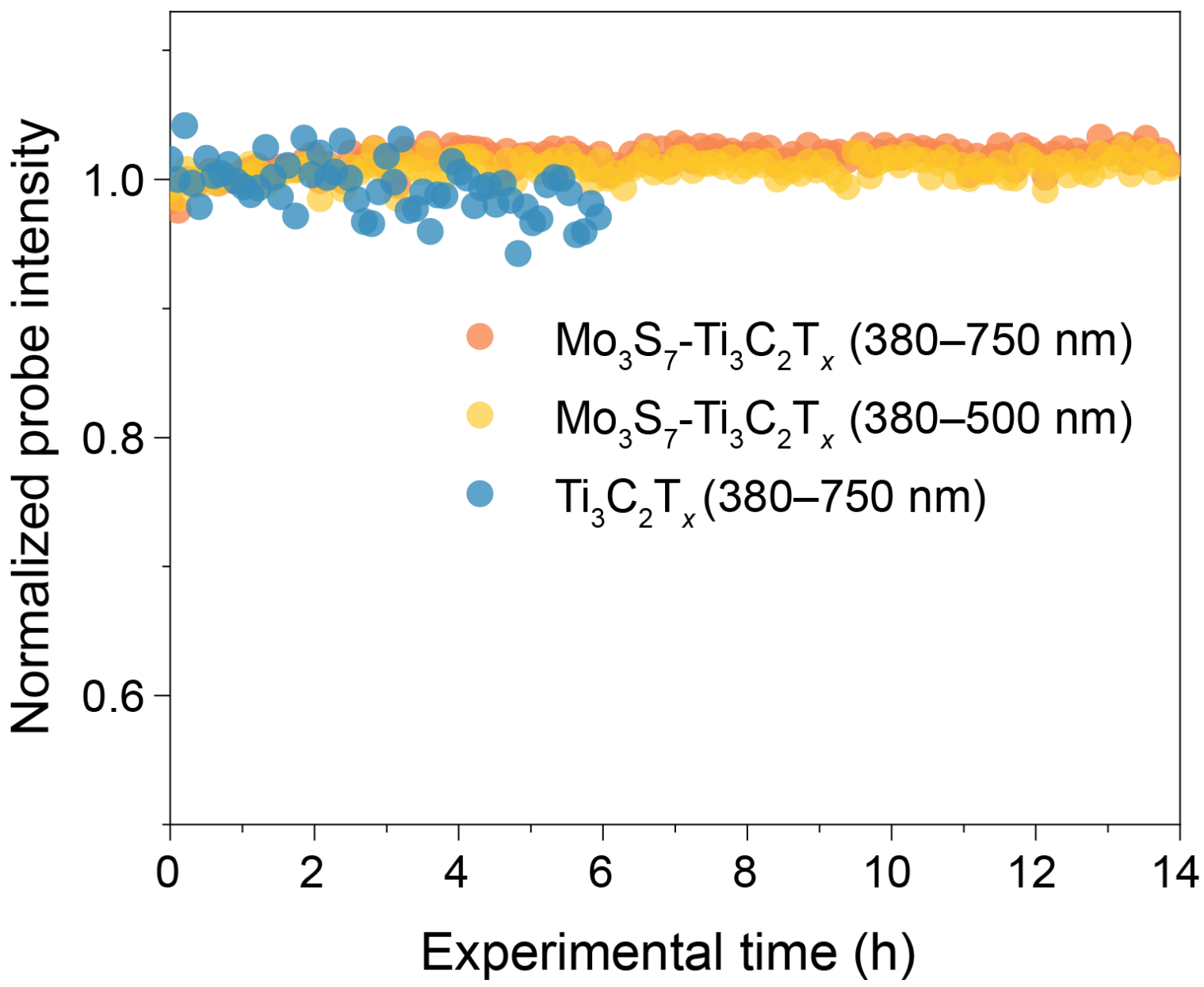

Supplementary Fig. 12 Photostability of $Ti_3C_2T_x$ and Mo_3S_7-$Ti_3C_2T_x$ under 266 nm excitation. Temporal evolution of the normalized probe intensity under 266 nm excitation for $Ti_3C_2T_x$ and Mo_3S_7-$Ti_3C_2T_x$, averaged over the indicated spectral ranges. The probe intensity remains constant over the entire measurement duration (~14 h), indicating no detectable sample degradation. Source data are provided as a Source Data file.

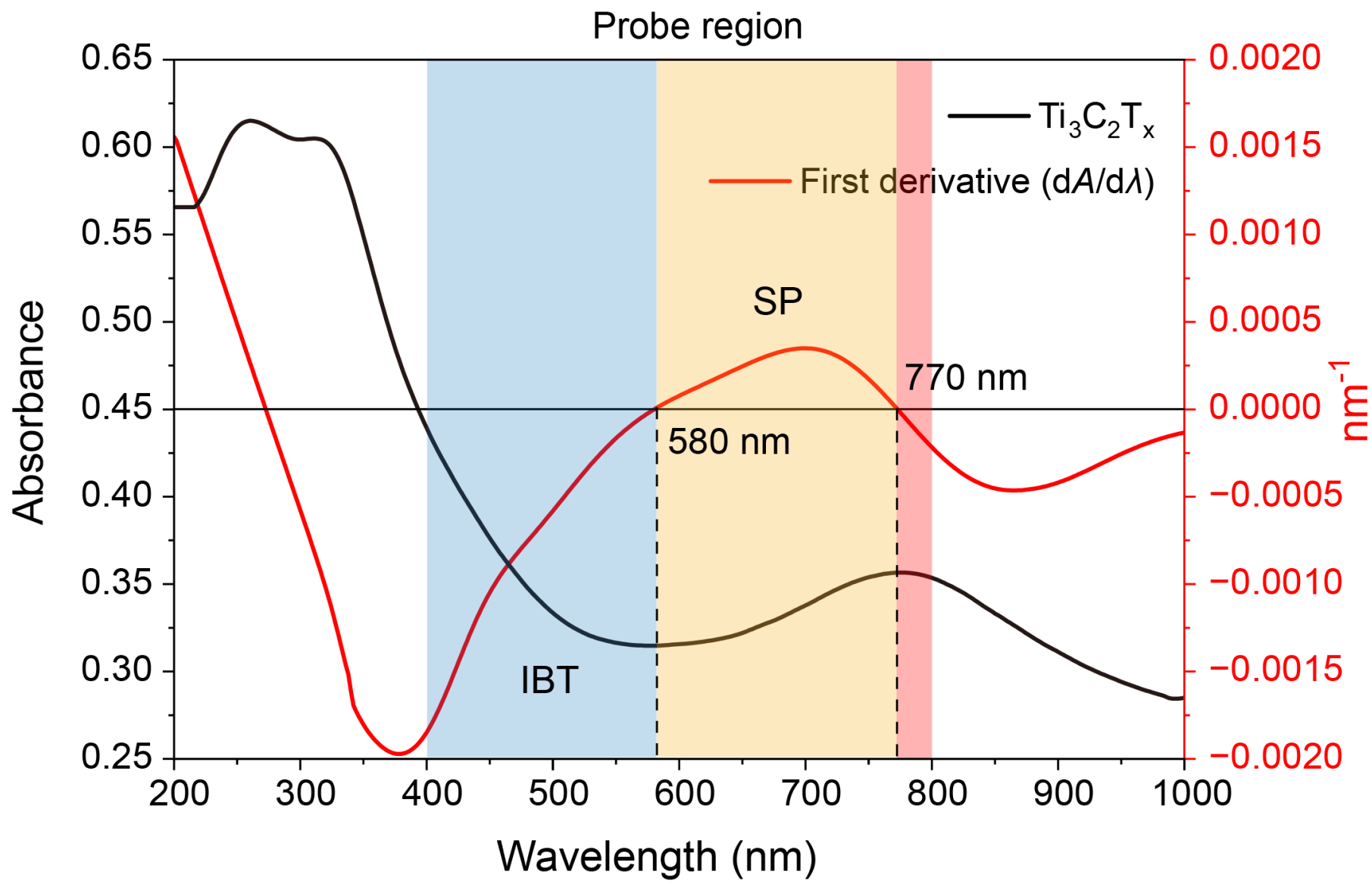

Supplementary Fig. 13 Steady-state absorption and derivative spectra of $Ti_3C_2T_x$. Steady-state absorption spectrum of $Ti_3C_2T_x$ (A) and its first derivative with respect to wavelength ($dA/d\lambda$), which reflects the spectral sensitivity of the optical response to small perturbations of the resonance energy. Source data are provided as a Source Data file.

First-derivative analysis of the steady-state absorption spectrum of $Ti_3C_2T_x$ with respect to wavelength, $dA/d\lambda$ exhibits zero crossings near ~580 nm and ~770 nm. Specifically, $dA/d\lambda < 0$ in the 400–580 nm region (IBT-dominated), whereas $dA/d\lambda > 0$ in the 580–770 nm region, where the SP contribution becomes dominant. This sign reversal implies that a perturbation of the optical resonance would produce oscillatory absorption changes with opposite signs on either side of the crossover wavelength, giving rise to an amplitude node and a π-phase inversion. The larger magnitude of $|dA/d\lambda|$ in the IBT-dominated region indicates a higher sensitivity of IBT-related transitions to lattice modulation. Nevertheless, further quantitative modeling would be valuable to fully disentangle these effects.

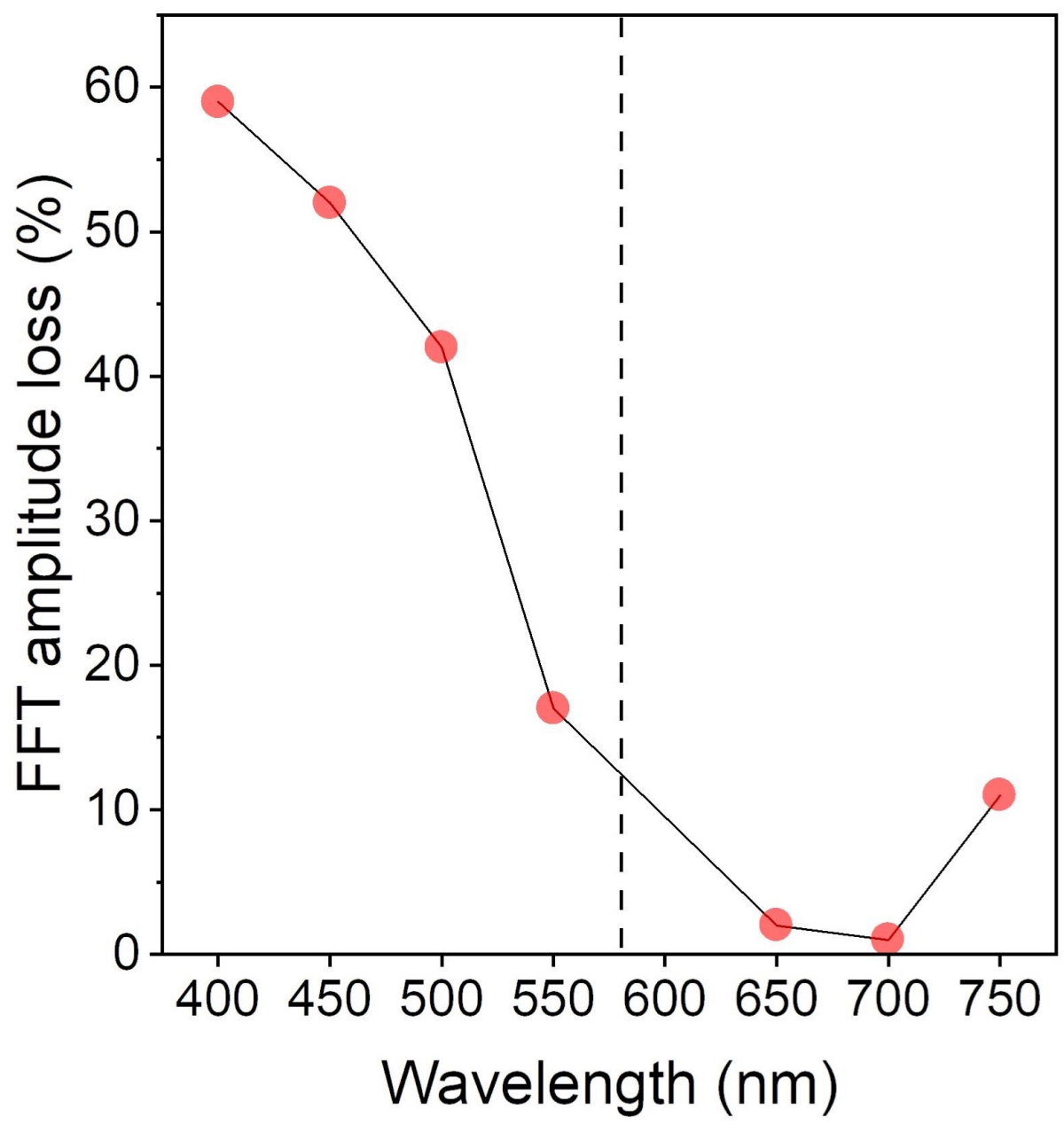

Supplementary Fig. 14 Probe-wavelength-dependent FFT amplitude loss in Mo_3S_7-$Ti_3C_2T_x$. Probe-wavelength-dependent FFT amplitude loss (%) of Mo_3S_7-$Ti_3C_2T_x$ relative to $Ti_3C_2T_x$ under 4.66 eV excitation. Source data are provided as a Source Data file.

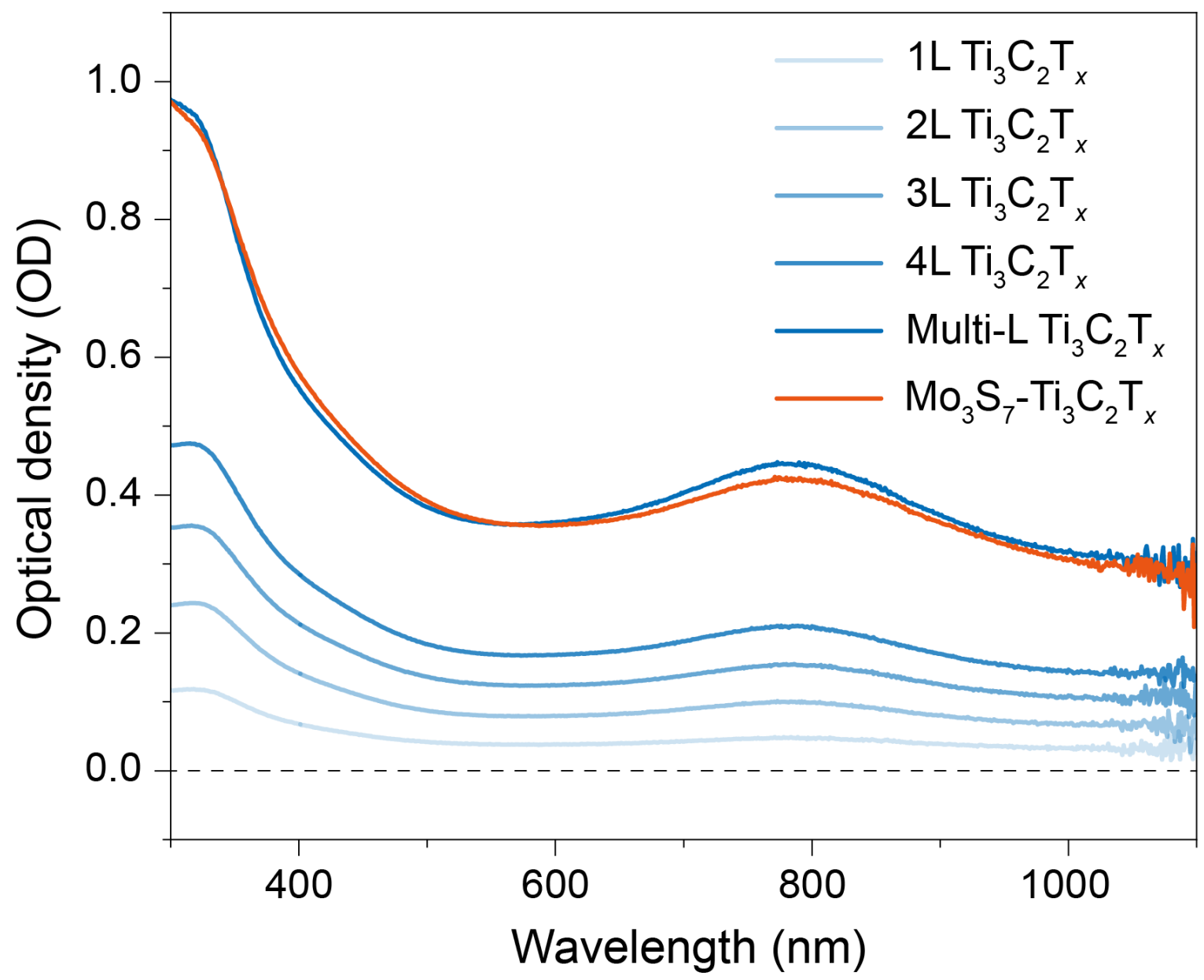

Supplementary Fig. 15 Optical-density-based thickness estimation of $Ti_3C_2T_x$ and Mo_3S_7-$Ti_3C_2T_x$ films. Layer-dependent UV–Vis optical density (OD) spectra of assembled $Ti_3C_2T_x$ films (as references), along with those of $Ti_3C_2T_x$ and Mo_3S_7-$Ti_3C_2T_x$ samples used in the OPTP measurements, revealing comparable thicknesses of approximately 8.5 layers for both $Ti_3C_2T_x$ and Mo_3S_7-$Ti_3C_2T_x$ samples. These OD spectra were used to determine the absorbed photon density (N_{abs}) at the pump wavelengths. Source data are provided as a Source Data file.

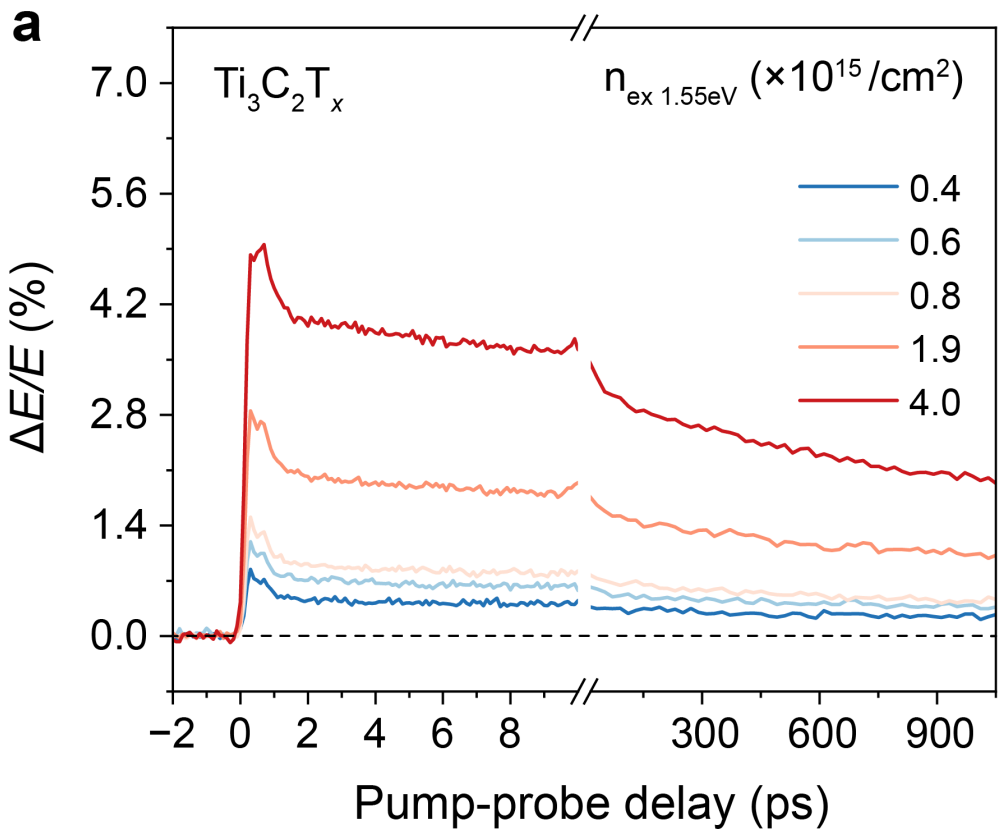

Supplementary Fig. 16 THz photoconductivity dynamics of $Ti_3C_2T_x$ and Mo_3S_7-$Ti_3C_2T_x$. Pump fluence-dependent OPTP results of **a** $Ti_3C_2T_x$ and **b** Mo_3S_7-$Ti_3C_2T_x$ under 800 nm excitation. Source data are provided as a Source Data file.

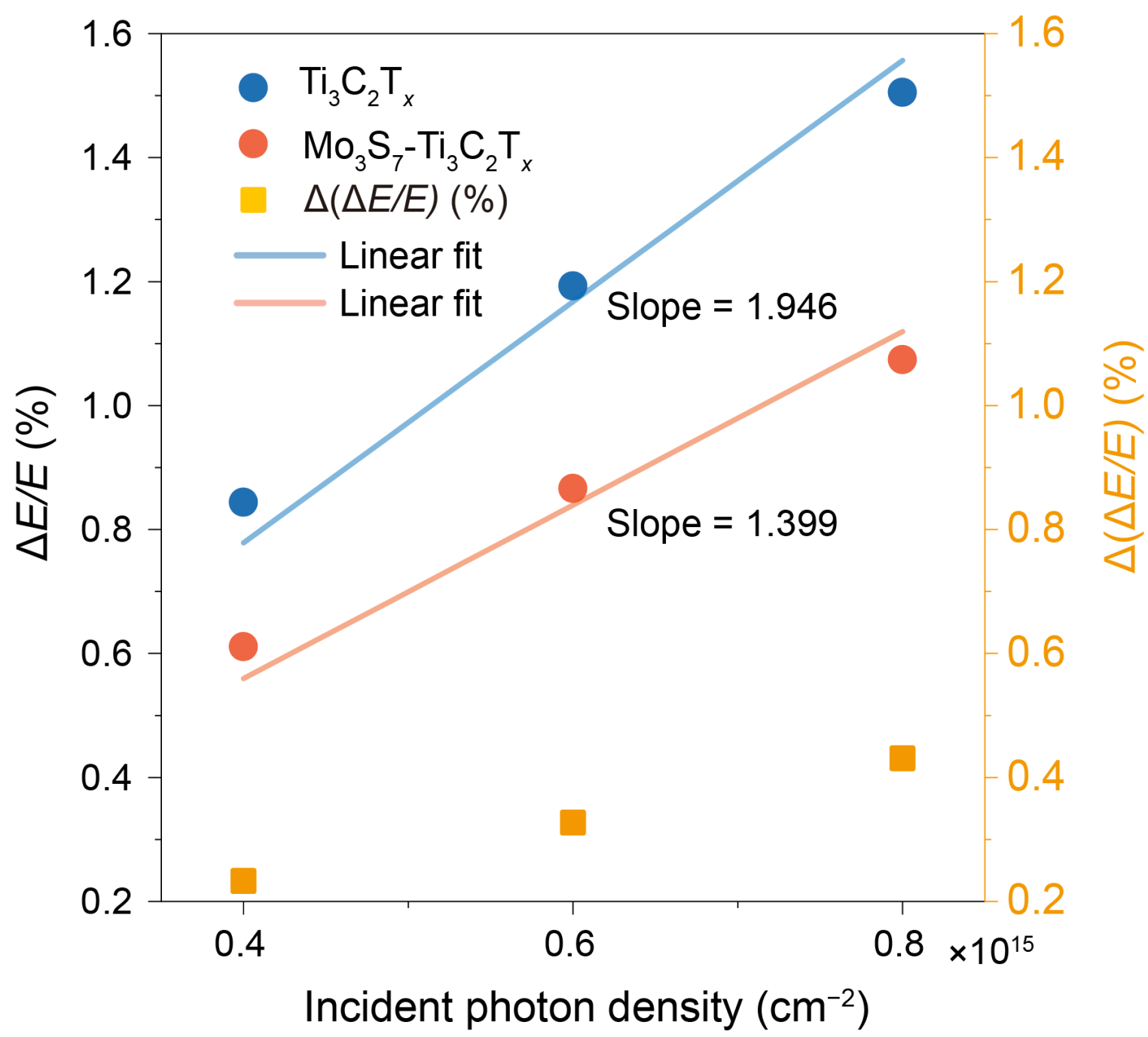

Supplementary Fig. 17 Fluence-dependent THz photoconductivity of $Ti_3C_2T_x$ and Mo_3S_7-$Ti_3C_2T_x$. Peak transient THz photoconductivity amplitude ($\Delta E/E$) (selected at 0.3 ps) as a function of incident photon density for pristine $Ti_3C_2T_x$ and Mo_3S_7-$Ti_3C_2T_x$. The smaller slope observed for Mo_3S_7-$Ti_3C_2T_x$ indicates a reduced sensitivity of the $T_{e,peak}$ to excitation fluence, consistent with interfacial energy and/or carrier transfer. Yellow squares show the fluence-dependent difference $\Delta(\Delta E/E)$ between the two samples, emphasizing the increasing suppression of electronic heating in the hybrid system at higher excitation densities. Source data are provided as a Source Data file.

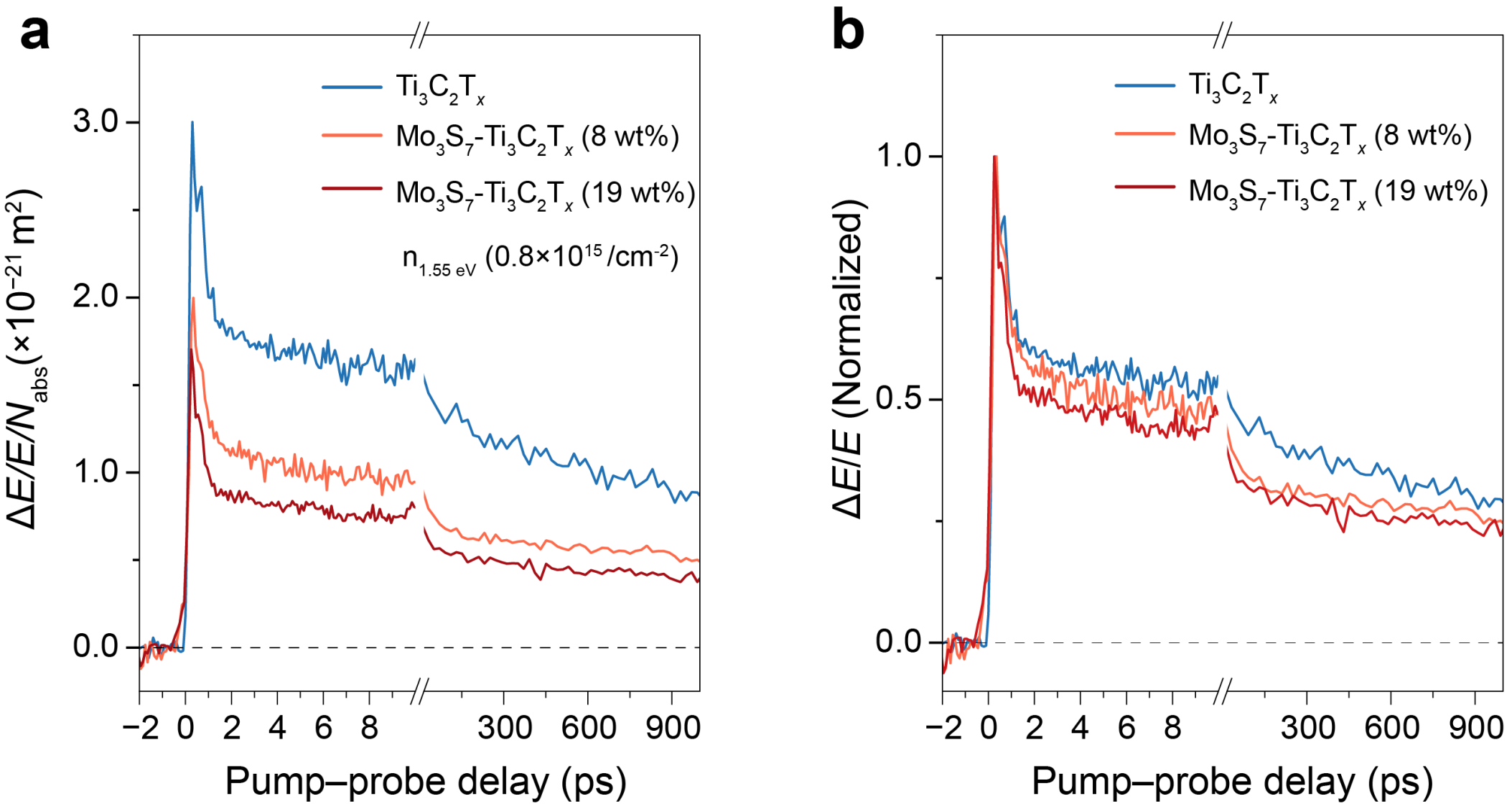

Supplementary Fig. 18 THz photoconductivity dynamics of Mo_3S_7-$Ti_3C_2T_x$. Mo_3S_7-loading-dependent THz photoconductivity dynamics of Mo_3S_7-$Ti_3C_2T_x$ under 800 nm (1.55 eV) excitation **a** without and **b** with normalization. Source data are provided as a Source Data file.

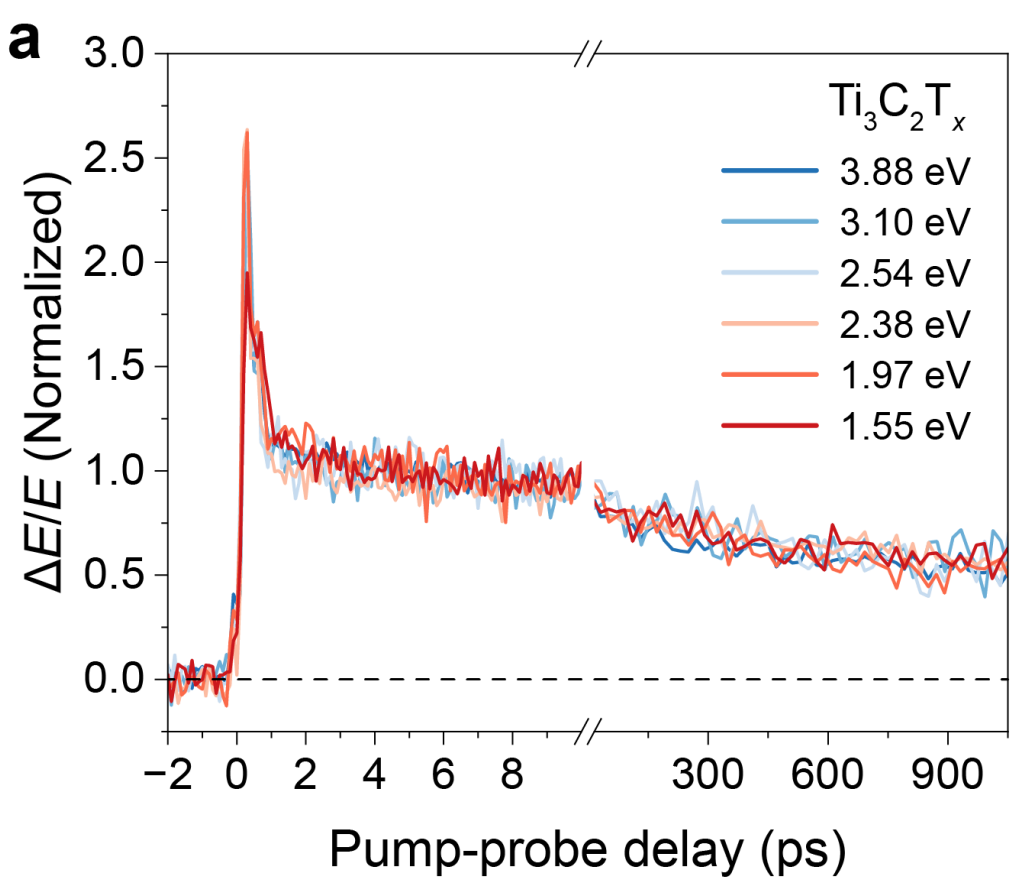

Supplementary Fig. 19 THz photoconductivity dynamics of $Ti_3C_2T_x$ and Mo_3S_7-$Ti_3C_2T_x$. Pump wavelength-dependent OPTP dynamics of **a** $Ti_3C_2T_x$ and **b** Mo_3S_7-$Ti_3C_2T_x$ under 0.8×10^{15} cm^{-2} photon excitation, normalized to the average signal in the 1–10 ps delay window. Source data are provided as a Source Data file.

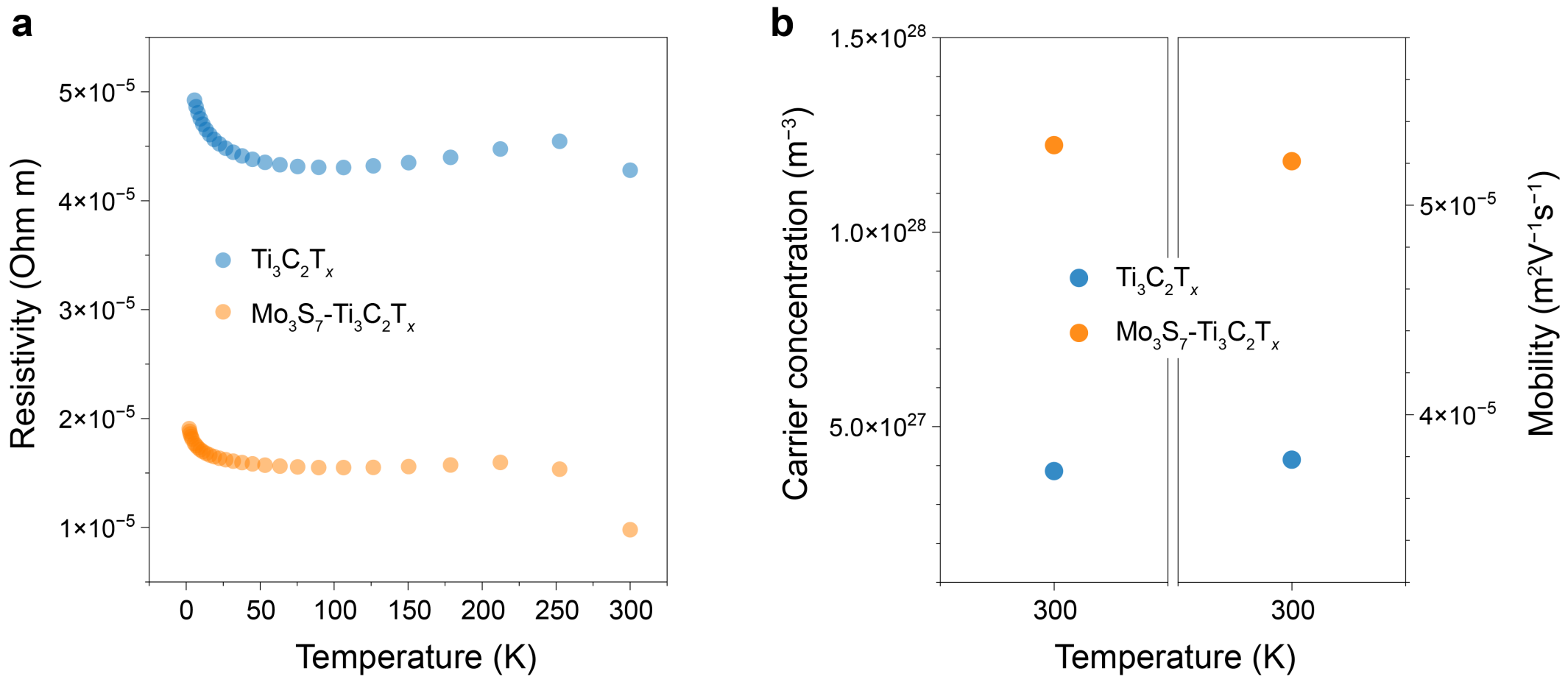

Supplementary Fig. 20 Electrical transport properties of $Ti_3C_2T_x$ and Mo_3S_7-$Ti_3C_2T_x$. a Temperature-dependent resistivity of $Ti_3C_2T_x$ and Mo_3S_7-$Ti_3C_2T_x$. **b** Hall carrier concentration (electrons) and Hall mobility at 300 K for $Ti_3C_2T_x$ and Mo_3S_7-$Ti_3C_2T_x$. Source data are provided as a Source Data file.

Supplementary Table 1. Statistical surface termination analysis of $Ti_3C_2T_x$ derived from quantitative XPS peak fitting. Source data are provided as a Source Data file.

**$Ti_3C_2T_x$**			**Mo_3S_7-$Ti_3C_2T_x$**		
O1s A<br>(–**O**)<br>At. %	O1s B<br>(–**OH/H_2O**)<br>At. %	F<br>(–**F**)<br>At. %	O1s A<br>(–**O**)<br>At. %	O1s B<br>(–**OH/H_2O**)<br>At. %	F<br>(–**F**)<br>At. %
7.82	9.39	10.29	6.80	11.99	7.38
(28.4 %)	(34.2%)	(37.4%)	(26.0%)	(45.8%)	(28.2%)

Deconvolution of the O 1s spectra reveals that pristine $Ti_3C_2T_x$ exhibits a mixed termination environment with an estimated O/OH/F ratio of approximately 28.4%/34.2%/37.4%. Oxygen-containing terminations (O + OH) collectively account for ~63% of the surface species, confirming their dominant presence prior to functionalization. After Mo_3S_7 immobilization, the termination distribution evolves to approximately 26.0%/45.8%/28.2%, with the fraction of oxygen-containing species increasing to ~72%. The relative increase in OH-related contributions is likely associated with enhanced hydration effects and expanded interlayer spacing upon Mo_3S_7 incorporation (Fig. 2d).

Although $Ti_3C_2O_2$ was adopted as a computationally tractable model, the potential influence of mixed surface terminations and hydration layers on interfacial pathways and the detailed electronic structure should be acknowledged.
