# Supplementary material for: Modulating nonequilibrium electron–phonon interactions and energy relaxation in MXenes by surface-anchored Mo3S7 nanoclusters
Source: Nat Commun. 2026 Jul 16;17:8829. doi: 10.1038/s41467-026-75614-4 (PMC13500858; doi:10.1038/s41467-026-75614-4)
Supplement: Supplementary file 2 [file 41467_2026_75614_MOESM2_ESM.pdf]

The Description of Additional Supplementary Files

Title: **Supplementary Data 1**

Description: Atomic coordinates of the optimized computational models of Mo_3S_7-$Ti_3C_2T_x$, including the 3 × 3 $Ti_3C_2O_2$ supercell with surface-anchored Mo_3S_7 nanoclusters.
