# Supplementary material for: Modulating nonequilibrium electron–phonon interactions and energy relaxation in MXenes by surface-anchored Mo3S7 nanoclusters
Source: Nat Commun. 2026 Jul 16;17:8829. doi: 10.1038/s41467-026-75614-4 (PMC13500858; doi:10.1038/s41467-026-75614-4)
Supplement: Supplementary file 4 [file 41467_2026_75614_MOESM5_ESM.pdf]

nature portfolio

Peer Review File

Modulating Nonequilibrium Electron–Phonon Interactions and Energy Relaxation in MXenes by Surface-Anchored Mo_3S_7 Nanoclusters

Corresponding Author: Professor Xinliang Feng

This file contains all reviewer reports in order by version, followed by all author rebuttals in order by version.

Version 0:

Reviewer comments:

Reviewer #1

(Remarks to the Author)
Zhang et al. present a well-designed and time-resolved study on modulating nonequilibrium electron–phonon interactions in Ti3C2Tx MXene through surface anchoring of Mo3S7 nanoclusters. The combination of femtosecond transient absorption and THz spectroscopy provides convincing evidence of an ultrafast (<100 fs) charge/energy extraction process that effectively suppresses electron–phonon coupling and enhances interfacial thermal transport. The results are interesting and potentially important for advancing the understanding of hot-carrier relaxation and energy management in 2D conductors. However, several clarifications and corrections are needed before publication:

Q1. lines 206-212, the authors stated that “The incorporation of Mo3S7 nanoclusters … indicating that the formation of the Mo3S7–Ti3C2Tx interface initiates an ultrafast process that competes with or modulates the hot carrier thermalization in Ti3C2Tx.” However, it is unclear how the authors ruled out the possibility that the observed dynamics changes arise from direct excitation of the Mo3S7 nanoclusters, but solely from interfacial charge/energy transfer. As shown in Fig. 3a, Mo3S7 exhibited strong absorption in the 250–350 nm range; thus, under 266 nm excitation, the signals from directly excited Mo3S7 may significantly influence both the carrier dynamics and coherent phonon behavior, potentially leading to misinterpretation. For example, the weaker signal of MXene–Mo3S7 observed in Figures 3e and 5d may be attributed to part of the pump light being absorbed by Mo3S7, which reduces the photon energy absorbed by Ti3C2Tx and leads to a decrease in the signal.

Therefore, it is recommended that the authors determine, under different excitation wavelengths (266, 400, and 800 nm), the contribution of Mo3S7 directly photoexcited by the pump light in the MXene–Mo3S7 sample to the transient absorption (TA) and optical-pump terahertz-probe (OPTP) signals, in order to obtain accurate conclusions.

Q2. The authors stated that “Quantitative EDS analysis yields a Mo/Ti atomic ratio of 0.034 (Supplementary Fig. 3), corresponding to an estimated 1.7% surface coverage of MXene terminations by Mo3S7 nanoclusters.” From this estimation, the probability of observing charge or energy transfer should be quite low, since the heterointerface accounts for only about 1.7% of the total surface area. However, in Fig. 3e, the fraction of the fast (~100 fs) electron–phonon interaction decreases from 58% to 42%, a change of about 16%. It remains unclear whether this variation is mainly caused by interfacial charge or energy transfer from MXene to Mo_3S_7. The possible influence of direct photoexcitation of Mo_3S_7 nanoclusters on the observed signals should also be further ruled out.

Q3. The authors could perform experiments using a longer pump wavelength. According to the calculated band structure shown in Fig. 5c, the bandgap of Mo3S7 is approximately 1 eV. Therefore, excitation with a laser of wavelength longer than 1300 nm would selectively excite the MXene while avoiding direct excitation of Mo3S7, providing more conclusive evidence for interfacial charge transfer. Additionally, it is recommended that the authors provide the transient dynamics of MXene and MXene–Mo_3S_7 under 400 nm and 800 nm excitation, similar to Figure 3e of the main text. According to the calculations in Figure 5c and the schematics in Figures 6d–e, charge transfer may also occur under the two excitation wavelengths, and examining how this affects the dynamics is also important.
Q4. What is the instrument response functon(IRF) in these experiments? If it is shorter than 60 fs, variations in the electron–phonon coupling times of MXene and MXene–Mo3S7 as a function of excitation fluence should be observable. If MXene absorbs more energy and can rapidly transfer it across the interface, one might expect the e–ph coupling in MXene–Mo_3S_7

to be less sensitive to excitation fluence than in pristine MXene. This could provide additional supporting evidence for interfacial carrier or energy transfer, as discussed in Giulia et al., Nature Materials 19, 1312–1318 (2020). However, it is also important to ensure that Mo3S7 does not directly absorb the pump photons in these measurements.

Q5. The authors mention that the incident photon fluence used for the OPTP measurements in Fig. 5c and d is the same for both MXene and MXene–Mo3S7 samples. However, are their absorption intensities also identical? In Fig. 3a and Supplementary Fig. 11, the absorption spectra are presented in arbitrary units, so the absorption intensities of MXene and MXene–Mo3S7 samples are unclear. It is recommended that the authors provide steady-state absorption spectra in the Supplementary Information, expressed in optical density (OD) units, to allow for an accurate comparison.

Reviewer #2

(Remarks to the Author)
The manuscript investigates the modulation of nonequilibrium electron–phonon (e–ph) interactions in Ti3C2Tx MXenes through surface-anchored Mo3S7 nanoclusters using ultrafast transient absorption, coherent phonon spectroscopy, and THz photoconductivity. The topic is timely and potentially impactful. However, several central issues remain unresolved. The mechanistic interpretation is insufficiently supported at the microscopic level, key connections to established nonthermal-electron relaxation physics in MXenes are missing, and alternative explanations for several observations are not adequately ruled out. Substantial revision would be required for publication in Nature Communications.

Major comments:

1. The central conclusion—that Mo3S7 nanoclusters competitively deplete nonthermal carriers on sub-100 fs timescales—relies solely on correlations between spectral attenuation and nanocluster anchoring. No quantitative energy-flow analysis or interfacial coupling estimates are provided to demonstrate that this channel genuinely competes with the intrinsic A_{1g}-mediated relaxation.

2. The reduced PIA amplitude may also arise from changes in transition dipole moments, local dielectric screening, Mo3S7 state filling, or interfacial hybridization. Without state-resolved probes (e.g., TR-XPS, TR-EELS, mid-IR signatures of interfacial charge), the assignment of PIA attenuation to carrier extraction remains speculative.

3. The π-phase shift and amplitude node around 580 nm are ascribed to IBT–SPR interference, yet no supporting modeling (e.g., vibronic modulation analysis or deformation-potential calculations) is presented. The observed behavior could also result from lattice softening, increased interlayer spacing, or modified plasmonic fields following Mo3S7 binding. These possibilities are not discussed.

4. The manuscript does not distinguish between electron transfer into Mo3S7 acceptor states, nonradiative plasmon-to-cluster energy transfer, phonon-mediated heat flow, or interfacial field quenching. Since no direct evidence of charge-separated species is provided, it remains unclear whether electrons, energy, or both are being transferred.

5. Critical controls are missing: MXene with an inert ligand of similar size to Mo3S7 (to decouple steric vs electronic effects), varying nanocluster coverage to show scaling of extraction rate, different terminations (F, OH, O) to test the dependence on surface electronic structure. Without these, it is difficult to attribute the observed modulation strictly to the Mo3S7–Ti3C2Tx interaction.

6. The negative photoconductivity in the OPTP measurements is attributed to enhanced extraction efficiency, but similar signals can arise from mobility loss, increased scattering, interfacial disorder, or changes in Drude weight. Frequency-resolved Drude–Smith analysis is needed to distinguish these effects and to justify the reported extraction efficiencies.

7. The assumption of a uniformly O-terminated MXene with clean Mo–O–Ti coordination is unrealistic. Mixed O/OH/F termination and hydration layers can strongly affect interfacial energetics and hot-carrier pathways A statistically meaningful surface-termination analysis (ToF-SIMS, STEM-EELS, or quantitative XPS peak fitting) is required to justify the structural model.

8. Compared with previous reports that show weak e–ph coupling in $Ti_3C_2T_x$ (e.g., THz studies), the presented results strongly emphasize suppression of A_{1g} coherent phonons. The manuscript fails to reconcile these discrepancies or provide a unified picture of how nanoclusters switch between strong and weak coupling regimes.

9. The manuscript relies on PBE-level electronic structures, which are known to be qualitatively unreliable for MXenes: semilocal GGA severely overdelocalizes Ti-3d states, underestimates d–d splittings and interband transition energies by several hundred meV, and fails to reproduce the termination-dependent crystal-field environment that governs A_{1g}/Eg phonon coupling and hot-electron relaxation pathways. Given the decisive influence of these d-manifold features on e–ph dynamics, plasmonic response, and TA/PIA assignment, PBE-based interpretations are fundamentally flawed and should be replaced with at least DFT+U or hybrid-DFT benchmarks.

Minor but Important Issues:

1. The reported “sub-100 fs extraction” is at or below the instrument response; deconvolution is insufficiently documented.

2. Spectral assignments (PIA-1/2/3) should be backed by calculations of excited-state absorption or joint DOS.

3. Potential nanocluster degradation under high-energy excitation (266 nm) is not addressed.

4. The Mo/Ti atomic ratio (0.034) suggests very low coverage; it is unclear how such sparse clustering produces the large phonon-amplitude suppression reported.

Reviewer #3

(Remarks to the Author)
The authors investigate how electron-phonon interactions in $Ti_3C_2T_x$ MXene can be tuned by modifying its surface with covalently attached Mo_3S_7 clusters. They use transient optical absorption to track photoinduced processes, specifically the excitation of coherent phonon modes. These measurements are complemented by optical pump–THz probe experiments that probe thermal relaxation. The topic is very interesting, and the data presented are of high quality, but at the current stage, the analysis and explanations provided are difficult to follow and unconvincing, raising concerns that the data may be over-interpreted.

1. The abstract is not a good representation of the paper's observations. Specifically, the statement: "Femtosecond transient absorption spectroscopy reveals an ultrafast, nonthermal electron/energy extraction, accompanied by probe wavelength-dependent suppression of the coherent A1g phonon mode." — is this observed in Mo_3S_7-modified MXene only, or in both modified and unmodified samples? How does the addition of Mo_3S_7 impact this behavior? Could you please rephrase this to ensure that your findings are unambiguous?
Similarly, the sentence: "Complementary optical-pump terahertz-probe measurements confirm sub-100 fs energy extraction, with efficiency increasing from ~28.6% at 1.55 eV to ~38.2% at 3.88 eV, driven by improved energetic alignment between the hot electrons in $Ti_3C_2T_x$ and the conduction-band manifold of Mo_3S_7 nanoclusters." — Again, is the efficiency increase driven by the increase in excitation energy, or by the presence of the nanoclusters? This needs clarification.

2. The authors state that the nanoclusters enhance interfacial thermal conductance and facilitate heat dissipation between MXene layers, and that hot photoexcited electrons rapidly transition to the nanoclusters, disrupting the rapid transfer of energy to the MXene crystal lattice that would otherwise result in heating. However, their observation (based on transient absorption spectroscopy) that the behavior of non-modified and nanocluster-modified MXene films differs only under 266 nm (4.66 eV) excitation and remains virtually unchanged under 400 nm or 800 nm excitation, raises questions. While this wavelength-dependent behavior is very interesting in its own right, it appears to limit the validity of the broader conclusion that surface-anchored nanoclusters "introduce a rapid energy harvesting pathway that even competes with intrinsic e-ph relaxation" to only the deep UV excitation regime. Could the authors please clarify or reconcile this apparent contradiction?

3. The authors discuss how electrons excited by surface plasmon (SP) absorption at ~780 nm and those excited by interband transitions behave differently. Could they please elaborate on this point? Specifically, interband excitation would be expected to generate electrons and holes with less excess kinetic energy, as compared to excitation by free-carrier (intraband or plasmonic) absorption, which typically leads to hotter carriers. Is this what the authors observe, and how is this distinction reflected in their data?
4. With 266 nm excitation, the main differences in the transient absorption spectra appear in the photoinduced absorption band (PIA-2) around 500 nm. Do the authors have insight into the origin and nature of this band?

5. The authors also note that the transient bleach at the plasmon resonance intensifies over the initial hundreds of femtoseconds, as clearly visible in the TA color maps. Could the authors comment on the processes at play here? In your view, does this imply that the increase in lattice temperature, leading to surface plasmon (SP) bleaching, takes this long to develop? Or could this apparent delay be influenced by the overlapping contribution from the PIA-3 band, which also extends into this spectral range? Clarifying this would help disentangle the spectral dynamics and better contextualize the competing processes.

6. When discussing the differences in coherent phonon oscillations between nanocluster-modified and unmodified MXene, the authors observe a pronounced effect in the interband transition (IBT)-dominated spectral range (<580 nm), where coherent phonon generation is suppressed in the modified MXene. This effect is attributed to efficient interfacial hot carrier harvesting from MXene to the nanoclusters. Could the authors please elaborate on why IBT transitions are particularly affected following 266 nm excitation, while SP (plasmonic) transitions appear relatively unaffected?

7. When analyzing the OPTP data under 800 nm excitation (Figs. 5a–c), the authors report a reduced overall amplitude of the photoinduced conductivity suppression in nanocluster-modified MXene, which they interpret as evidence of a rapid (~100 fs), non-thermal charge/energy extraction pathway. They also observe an accelerated decay of the photoexcited conductivity suppression in the modified sample. However, this interpretation appears to contradict the transient absorption (TA) data, where no significant changes are reported for 800 nm excitation (Figs. S8 and S9).
Furthermore, the conclusions from the OPTP measurements are based on changes in the magnitude of photoinduced conductivity, calculated as the change in amplitude per injected carrier density at different excitation photon energies. Could the authors clarify how this injected carrier density was determined? Since the steady-state absorption spectrum (Fig. 3a) is normalized to the SPR peak, it is difficult to assess the role of potential differences in absolute absorption between the modified and unmodified films at various excitation wavelengths.

Version 1:

Reviewer comments:

Reviewer #1

(Remarks to the Author)
I throughout read all the responses and the revised manuscript, I found the authors have carefully revised the manuscipt according the Reviewer 1's comments. I am also very satisfied with the author's response to the reviewer 2's concerns.I like to recommend accepting this manuscript for publication with the present version.

Reviewer #3

(Remarks to the Author)
The authors answered my questions. The revised manuscript will be of great interest to the MXene and ultrafast spectroscopy communities. I support publication in its present form.

Open Access This Peer Review File is licensed under a Creative Commons Attribution 4.0 International License, which permits use, sharing, adaptation, distribution and reproduction in any medium or format, as long as you give appropriate credit to the original author(s) and the source, provide a link to the Creative Commons license, and indicate if changes were made.
In cases where reviewers are anonymous, credit should be given to 'Anonymous Referee' and the source.
The images or other third party material in this Peer Review File are included in the article’s Creative Commons license, unless indicated otherwise in a credit line to the material. If material is not included in the article’s Creative Commons license and your intended use is not permitted by statutory regulation or exceeds the permitted use, you will need to obtain permission directly from the copyright holder.
To view a copy of this license, visit https://creativecommons.org/licenses/by/4.0/

To Reviewer 1:

Zhang et al. present a well-designed and time-resolved study on modulating nonequilibrium electron–phonon interactions in $Ti_3C_2T_x$ MXene through surface anchoring of Mo_3S_7 nanoclusters. The combination of femtosecond transient absorption and THz spectroscopy provides convincing evidence of an ultrafast (<100 fs) charge/energy extraction process that effectively suppresses electron–phonon coupling and enhances interfacial thermal transport. The results are interesting and potentially important for advancing the understanding of hot-carrier relaxation and energy management in 2D conductors. However, several clarifications and corrections are needed before publication:

Response: We appreciate the positive evaluation and constructive feedback from the reviewer, which is invaluable in improving the quality of our manuscript. All the raised concerns have been addressed through additional experiments and discussions. Details can be seen in the point-by-point responses below.

Q1. lines 206-212, the authors stated that "The incorporation of Mo_3S_7 nanoclusters … indicating that the formation of the Mo_3S_7–$Ti_3C_2T_x$ interface initiates an ultrafast process that competes with or modulates the hot carrier thermalization in $Ti_3C_2T_x$." However, it is unclear how the authors ruled out the possibility that the observed dynamics changes arise from direct excitation of the Mo_3S_7 nanoclusters, but solely from interfacial charge/energy transfer. As shown in Fig. 3a, Mo_3S_7 exhibited strong absorption in the 250–350 nm range; thus, under 266 nm excitation, the signals from directly excited Mo_3S_7 may significantly influence both the carrier dynamics and coherent phonon behavior, potentially leading to misinterpretation. For example, the weaker signal of MXene–Mo_3S_7 observed in Figures 3e and 5d may be attributed to part of the pump light being absorbed by Mo_3S_7, which reduces the photon energy absorbed by $Ti_3C_2T_x$ and leads to a decrease in the signal.

Therefore, it is recommended that the authors determine, under different excitation wavelengths (266, 400, and 800 nm), the contribution of Mo_3S_7 directly photoexcited by the pump light in the MXene–Mo_3S_7 sample to the transient absorption (TA) and optical-pump terahertz-probe (OPTP) signals, in order to obtain accurate conclusions.

Response: We sincerely thank the reviewer for this insightful comment. We agree that, given the strong absorption of Mo_3S_7 nanoclusters in the 250 ~ 350 nm region (**Fig. 3a**), direct excitation under 266 nm pumping must be carefully considered. Notably, multiple independent observations indicate that the dynamic changes observed in Mo_3S_7-$Ti_3C_2T_x$ relative to $Ti_3C_2T_x$ do not arise from direct excitation of

Postadresse:
Technische Universität Dresden, 01062 Dresden
Besucheradresse:
Mommsenstr. 4, Zi. 216, Walther-Hempel-Bau, 01069 Dresden
https://tu-dresden.de/mn/chemie/mc/mc2

Postadresse:
Max-Planck-Institut für Mikrostrukturphysik, Weinberg 2, 06120 Halle
Besucheradresse:
Max-Planck-Institut für Mikrostrukturphysik, Weinberg 2, 06120 Halle
https://www.mpi-halle.mpg.de/smfd-de

Mo_3S_7 nanoclusters, but instead reflect modified energy relaxation pathways in $Ti_3C_2T_x$ induced by interfacial coupling.

First, the transient absorption spectra reveal a selective attenuation of the PIA-1 and PIA-2 bands in Mo_3S_7-$Ti_3C_2T_x$, whereas the PIA-3 band does not exhibit comparable suppression (**Fig. 3c**). If a simple "photon-sharing" mechanism was dominant, in which absorption by Mo_3S_7 merely reduces the excitation density reaching $Ti_3C_2T_x$, a uniform attenuation across all PIA features would be expected. The experimentally observed band-selective suppression is inconsistent with this scenario. Moreover, FFT analysis of the A_{1g} coherent phonon mode reveals an asymmetric reduction in spectral intensity rather than a uniform decrease (**Fig. 4e and f**), further ruling out a trivial excitation-density effect and instead indicating altered carrier-phonon coupling dynamics arising from interfacial interactions.

Second, OPTP spectroscopy provides direct insight into pump-induced photoconductivity and thus the energy retained in the electronic system. The $Ti_3C_2T_x$ and Mo_3S_7-$Ti_3C_2T_x$ samples used in these measurements were prepared with comparable MXene thickness (~8.5 layers), and the dynamics were normalized to the absorbed photon density (N_{abs}) to ensure a rigorous quantitative comparison. Under both 1.55 eV and 1.97 eV excitation, photon energies at which Mo_3S_7 clusters exhibit negligible absorption (more detailed explanation is also provided in our response to your **Q3**), and the hybrid system consistently shows a pronounced reduction in photoconductivity amplitude together with accelerated recovery dynamics (**Fig. 5c**). These results strongly indicate that the observed changes arise from interfacial electronic interactions rather than from direct excitation of the nanoclusters.

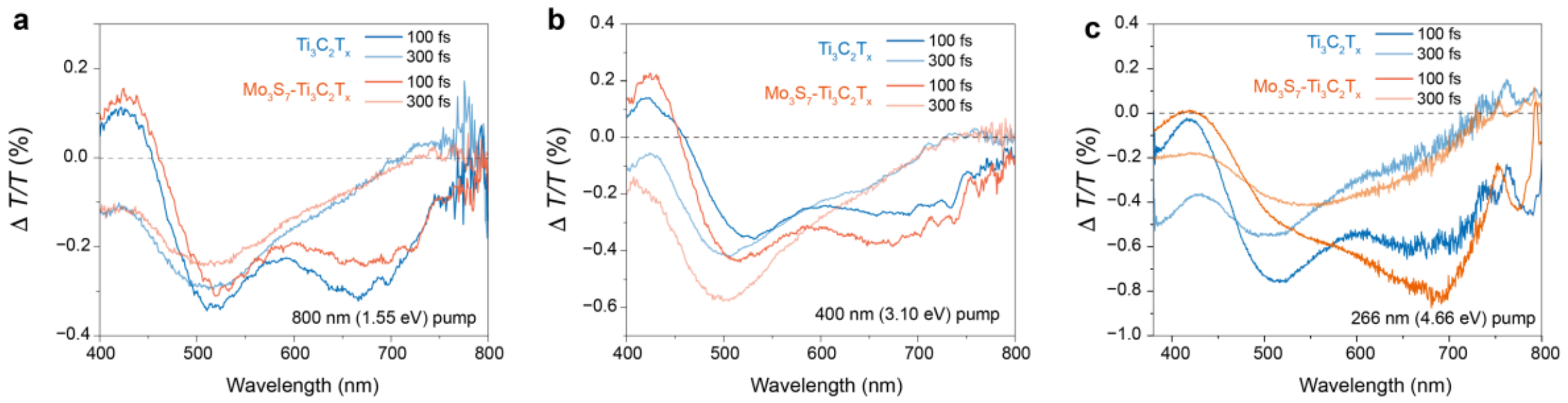

Fig. R1 $\Delta T/T$ spectra of $Ti_3C_2T_x$ and Mo_3S_7-$Ti_3C_2T_x$ at selected time delays of 100 fs and 300 fs under the excitation at **a**, 800 nm (1.55 eV), **b**, 400 nm (3.10 eV), and **c** 266 nm (4.66 eV).

We nevertheless considered the possibility that excited-state Mo_3S_7 nanoclusters could influence the spectral response through overlap effects. For example, a positive ground-state bleach of Mo_3S_7 could in principle spectrally overlap with PIA-1 and produce apparent attenuation. To examine this possibility, we followed the reviewer's suggestion and compared TA spectra of $Ti_3C_2T_x$ and Mo_3S_7-$Ti_3C_2T_x$ acquired under 800 nm, 400 nm, and 266 nm excitation. Under 800 nm excitation (**Fig. R1a**), where Mo_3S_7 is not

directly excited, the reduction in PIA intensity persists and qualitatively resembles that observed under 266 nm excitation, indicating that the effect does not rely on nanocluster excitation. In contrast, under 400 nm excitation (**Fig. R1b**), where direct excitation of Mo_3S_7 becomes possible, PIA-2 and PIA-3 display enhanced intensity and a noticeable blue shift of PIA-2, a behavior distinct from that observed under 266 nm excitation (**Fig. R1c**). Furthermore, under 266 nm excitation, the MXene contribution is expected to be even stronger than that under 400 nm excitation, such that any Mo_3S_7-related features at around ~510 nm would be expected to manifest as an additional negative contribution rather than the selective attenuation observed experimentally.

Taken together, while direct excitation of Mo_3S_7 under UV pumping may contribute marginally to the overall energy distribution, it cannot account for the correlated changes in amplitude, relaxation kinetics, coherent phonon behavior, and excitation-wavelength dependence consistently observed across both TA and OPTP measurements. Although Mo_3S_7 nanoclusters may influence the spectral response through local electronic structure modification and new interfacial configurations, the interpretation that most comprehensively explains all experimental findings is that formation of the Mo_3S_7-$Ti_3C_2T_x$ interface introduces an ultrafast interfacial energy and/or carrier harvesting pathway. This pathway competes with intrinsic relaxation channels in $Ti_3C_2T_x$ and thereby strongly modulates its nonthermal carrier relaxation dynamics. The corresponding clarification has been added into the revised manuscript (**Supplementary Fig. 11**).

Q2. The authors stated that “Quantitative EDS analysis yields a Mo/Ti atomic ratio of 0.034 (Supplementary Fig. 3), corresponding to an estimated 1.7% surface coverage of MXene terminations by Mo_3S_7 nanoclusters.” From this estimation, the probability of observing charge or energy transfer should be quite low, since the heterointerface accounts for only about 1.7% of the total surface area. However, in Fig. 3e, the fraction of the fast (~100 fs) electron–phonon interaction decreases from 58% to 42%, a change of about 16%. It remains unclear whether this variation is mainly caused by interfacial charge or energy transfer from MXene to Mo_3S_7. The possible influence of direct photoexcitation of Mo_3S_7 nanoclusters on the observed signals should also be further ruled out.

Response: We appreciate the constructive comment of the reviewer. The initially reported value of 1.7% was derived from the quantified Mo/Ti atomic ratio (0.034) obtained from elemental analysis. This value corresponds to a stoichiometric ratio calculated under two simplified assumptions: 1) O is the only surface termination species on $Ti_3C_2T_x$ (i.e., $Ti_3C_2O_2$), and 2) all O terminations are equally accessible and capable of coordinating with Mo_3S_7 nanoclusters. Therefore, the reported 1.7% represents a stoichiometric fraction rather than true geometric surface coverage. In realistic systems, the number of sterically accessible and chemically effective O coordination sites is substantially lower than the total

number of O terminations. Based on the simulated geometric structural models (**Fig. R2**), we estimate that only approximately 11.1% of O terminations can function as effective coordination sites for Mo_3S_7 nanoclusters at maximum packing density. Within this framework, the Mo_3S_7 loading in our sample corresponds to 15.3% utilization of the effective O coordination sites. Furthermore, $Ti_3C_2T_x$ typically contains mixed surface terminations, including –F, –Cl, and –OH groups, which are unlikely to effectively anchor Mo_3S_7 nanoclusters. The presence of these less-coordinating terminations further reduces the number of available anchoring sites and thus lowers the realistic geometric surface coverage.

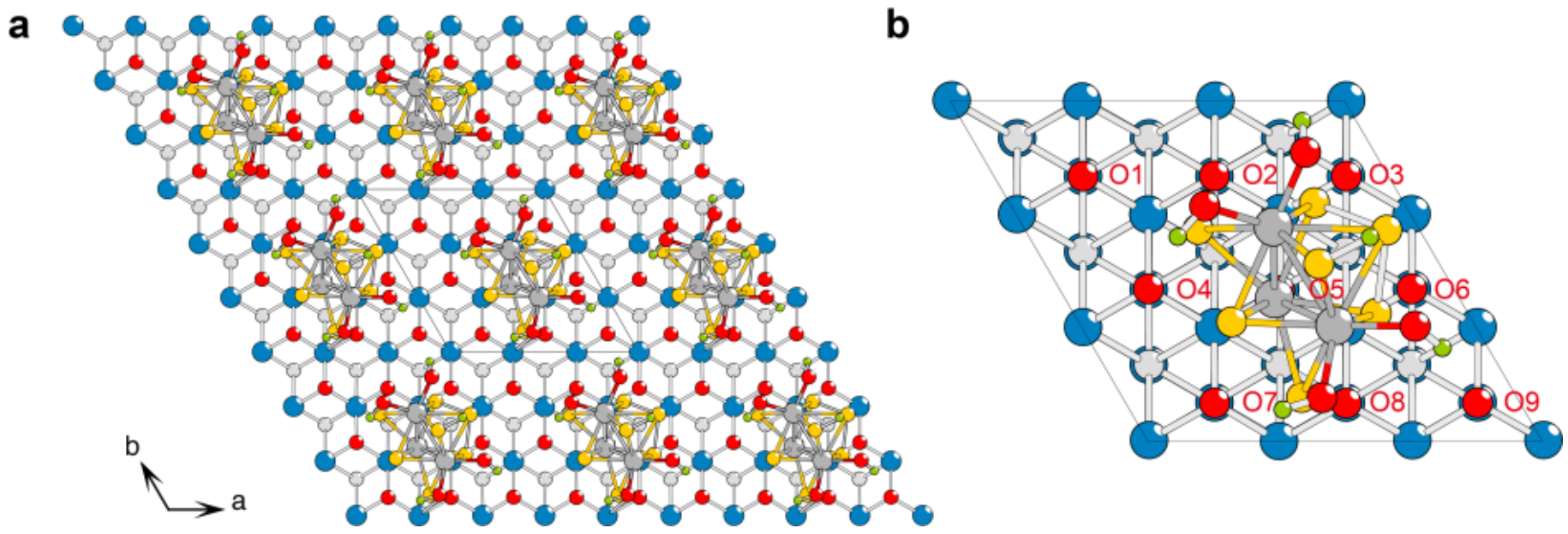

Fig. R2 a, Optimized model showing the maximum achievable packing density of Mo_3S_7 nanoclusters on $Ti_3C_2O_2$ when steric constraint is considered. **b**, All O terminations in a representative unit cell of Mo_3S_7–$Ti_3C_2O_2$, illustrating that only one-ninth of the O sites are sterically accessible and effective for coordination with Mo_3S_7 nanoclusters.

We acknowledge that describing the 1.7% stoichiometric ratio as "surface coverage" may have caused confusion, since this value does not directly represent geometric area coverage. To avoid ambiguity, we have revised the manuscript to describe the Mo_3S_7 loading primarily in terms of mass fraction (8 wt%), which provides a more intuitive and model-independent metric (**Page 6, Paragraph 1**). Although the absolute surface coverage is relatively low, we believe that this amount of Mo_3S_7 is sufficient to induce effective charge/energy transfer and pronounced modulation of electron-phonon (e-ph) interactions. Previous studies have demonstrated that comparable levels of interfacial modification can substantially alter carrier relaxation dynamics. For example, Mo_3S_{13} cluster-functionalized TiO_2 with 1.78 wt% loading achieves near-optimal photocatalytic H_2 evolution rates of ~65 μmol h^{-1}, indicating highly efficient charge transfer.[1] In addition, 0.5 wt% Pt-loaded $Ti_3C_2T_x$ has been reported to markedly influence electron–phonon interactions via efficient interfacial charge transfer.[2] These early published results collectively demonstrate that even small amounts of surface functionalization can substantially affect the electronic dynamics of host materials. The corresponding justification has been added to the revised manuscript (**Page 10**, **Paragraph 2**)

Q3. The authors could perform experiments using a longer pump wavelength. According to the calculated band structure shown in Fig. 5c, the bandgap of Mo_3S_7 is approximately 1 eV. Therefore, excitation with a laser of wavelength longer than 1300 nm would selectively excite the MXene while avoiding direct excitation of Mo_3S_7, providing more conclusive evidence for interfacial charge transfer. Additionally, it is recommended that the authors provide the transient dynamics of MXene and MXene–Mo_3S_7 under 400 nm and 800 nm excitation, similar to Figure 3e of the main text. According to the calculations in Figure 5c and the schematics in Figures 6d–e, charge transfer may also occur under the two excitation wavelengths, and examining how this affects the dynamics is also important.

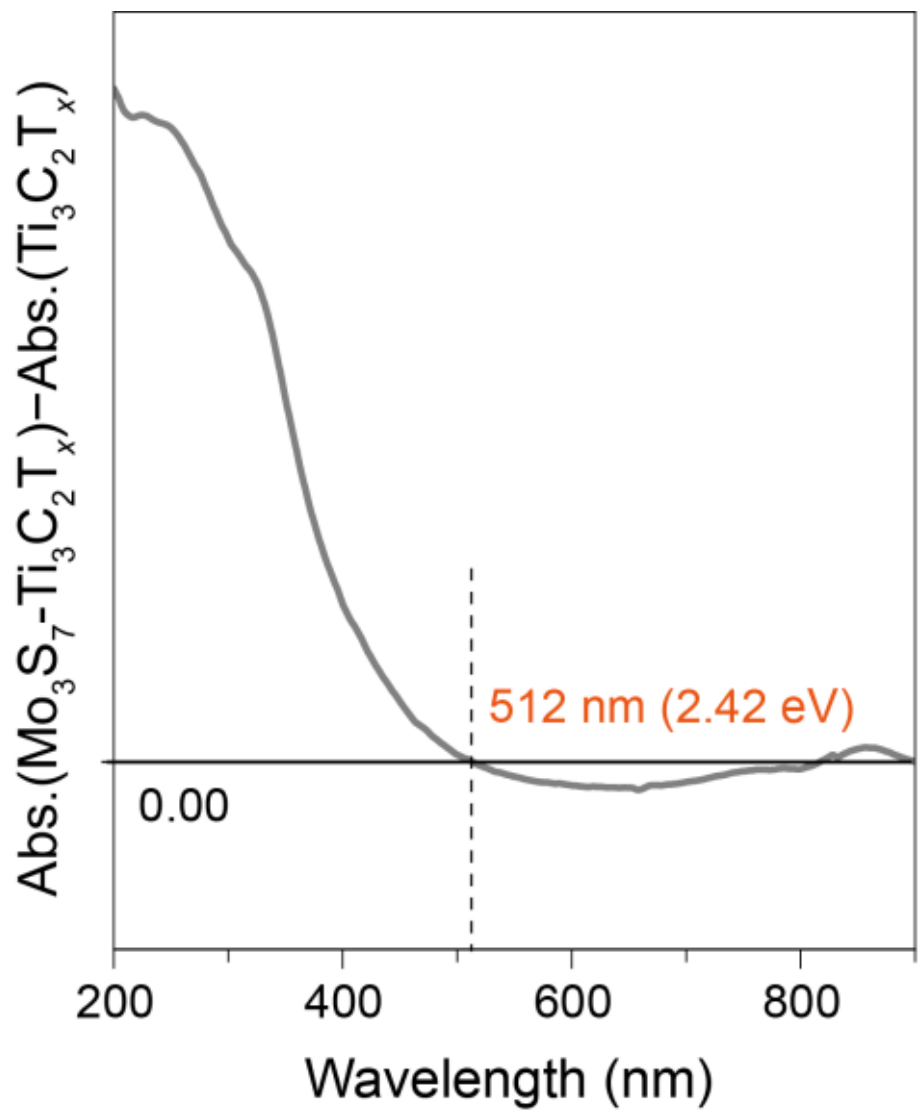

Fig. R3 Normalized difference absorption spectrum of Mo_3S_7-$Ti_3C_2T_x$ relative to pristine $Ti_3C_2T_x$, obtained after normalization to the absorbance at900 nm (1.38 eV).

Response: We would like to clarify that the bandgap of Mo_3S_7 (~1.5 eV) shown in **Fig. 5c** was estimated using semi-local PBE calculations. It has been known that this calculation method, even with the improved DFT+U approach, tends to underestimate bandgaps. Moreover, because the Mo_3S_7 nanoclusters are covalently grafted on $Ti_3C_2T_x$ from the $Mo_3S_7Br_6$ precursor, direct experimental determination of the intrinsic bandgap of the grafted clusters is not feasible in the present system. To better evaluate the possible spectral contribution of Mo_3S_7, we adopted an optical differential analysis. Considering negligible Mo_3S_7 absorption at 900 nm (1.38 eV), we normalized the steady-state absorption spectra of $Ti_3C_2T_x$ and Mo_3S_7-$Ti_3C_2T_x$ at this wavelength. The resulting normalized difference spectrum (**Fig. R3**) indicates that additional absorption attributable to Mo_3S_7 emerges predominantly at wavelengths shorter than 512 nm (~2.42 eV). This analysis indicates that Mo_3S_7 exhibits negligible or extremely weak absorption in the near-infrared region, particularly at 800 nm (1.55 eV). Therefore, 800 nm excitation can be considered to predominantly excite the $Ti_3C_2T_x$ host, while

largely avoiding direct excitation of the Mo_3S_7 nanoclusters. The corresponding discussion has been added to the revised manuscript (**Page 9**, **Paragraph 1**).

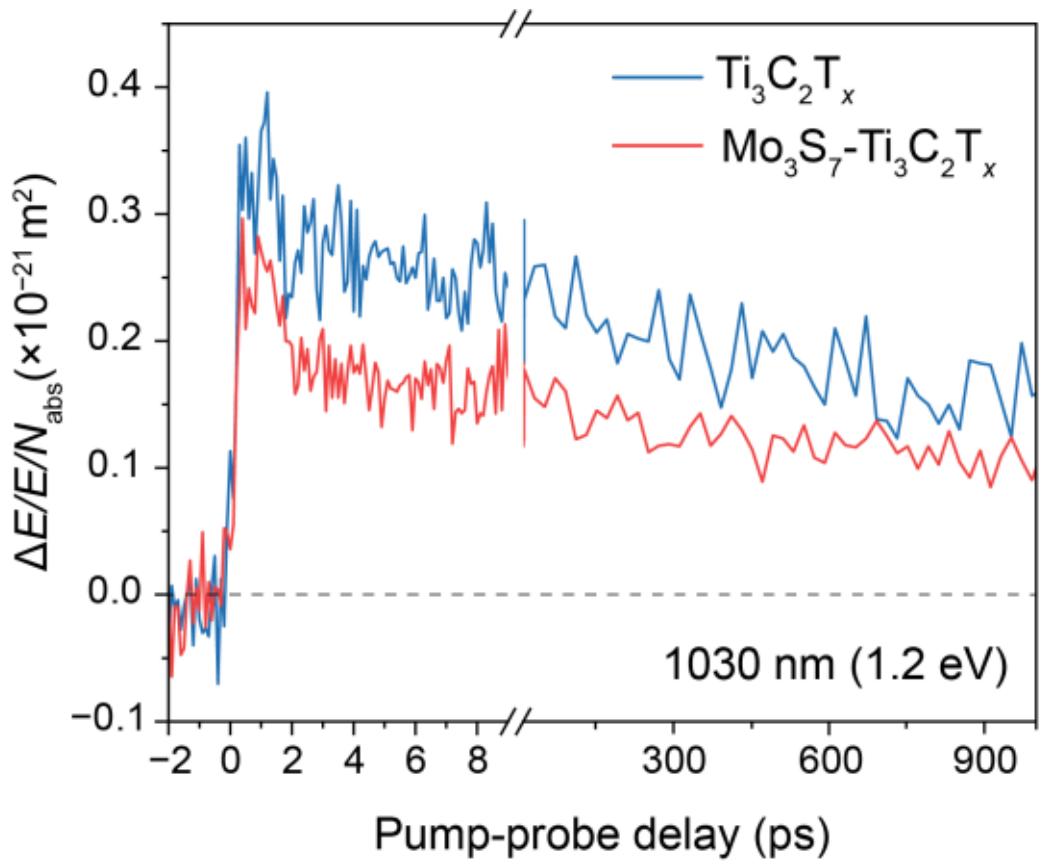

Fig. R4 THz photoconductivity dynamics of $Ti_3C_2T_x$ and Mo_3S_7-$Ti_3C_2T_x$ under 1030 nm (1.2 eV) excitation (incident photon density: 0.8×10^{15} cm^{-2}).

We fully agree that excitation at wavelengths longer than 1300 nm would further suppress any residual cluster absorption. However, due to the limited pump fluence available in our current setup, such measurements are not experimentally feasible at present. As an alternative approach, we performed additional OPTP measurements under 1030 nm (1.2 eV) excitation. As shown in **Fig. R4**, the Mo_3S_7-$Ti_3C_2T_x$ sample still exhibits a clear suppression of the photoconductivity peak amplitude compared to pristine $Ti_3C_2T_x$, consistent with the proposed interfacial modulation mechanism. Because 1030 nm excitation lies well below the estimated absorption onset of Mo_3S_7, these results provide further evidence that the observed dynamics are not driven by direct nanocluster excitation.

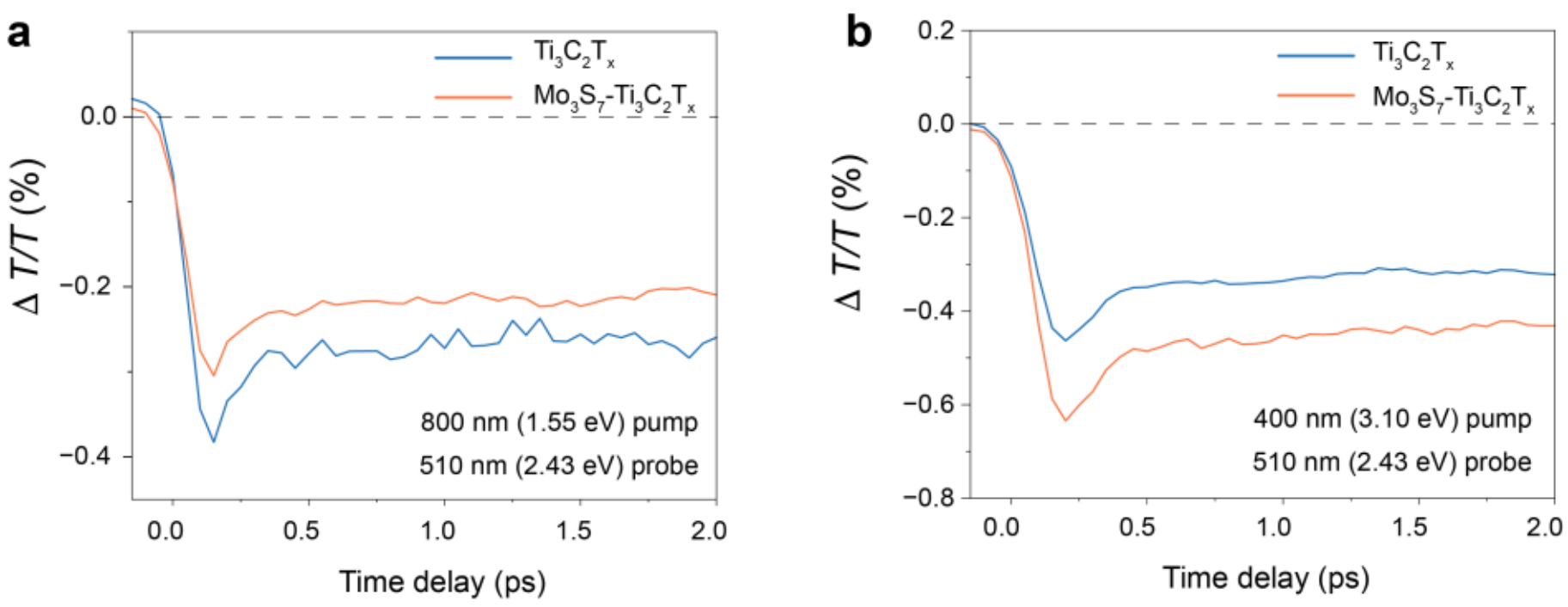

Fig. R5 Kinetic traces of $Ti_3C_2T_x$ and Mo_3S_7-$Ti_3C_2T_x$, probed at 510 nm (2.43 eV) under **a**, 800 nm (1.55 eV) and **b**, 400 nm (3.10 eV) excitation.

Regarding the reviewer's request for transient dynamics under 400 nm and 800 nm excitation, the corresponding $\Delta T/T$ spectral comparisons have been discussed in our response to your **Q1**. In addition, we now provide kinetic traces probed at 510 nm (2.43 eV) under both 400 nm and 800 nm excitation (**Fig. R5**). Kinetic analysis of the PIA-2 decay (probed at 510 nm) reveals a similar attenuation of the $\Delta T/T$ amplitude under 800 nm (1.55 eV) excitation, whereas the $\Delta T/T$ amplitude is enhanced under 400 nm (3.10 eV) excitation. The corresponding discussion has been added to the revised manuscript (**Supplementary Fig. 11**).

Q4. What is the instrument response function (IRF) in these experiments? If it is shorter than 60 fs, variations in the electron–phonon coupling times of MXene and MXene–Mo_3S_7 as a function of excitation fluence should be observable. If MXene absorbs more energy and can rapidly transfer it across the interface, one might expect the e–ph coupling in MXene–Mo_3S_7 to be less sensitive to excitation fluence than in pristine MXene. This could provide additional supporting evidence for interfacial carrier or energy transfer, as discussed in Giulia et al., Nature Materials 19, 1312–1318 (2020). However, it is also important to ensure that Mo_3S_7 does not directly absorb the pump photons in these measurements.

Response: Thank you for the thoughtful comment and for directing us to the important literature reference. In our study, transient dynamics was fitted using a Gaussian IRF with a full width at half maximum (FWHM) of 50 fs. As discussed in the mentioned literature, ultrafast interfacial extraction of hot carriers can effectively reduce the peak electronic temperature ($T_{e,peak}$) reached immediately after photoexcitation. This suppression of electronic heating modifies the subsequent thermalization pathway and typically manifests as a weaker dependence of the electron–phonon coupling time (τ_{e-ph}) on excitation fluence. In our OPTP measurements, a positive $\Delta E/E$ signal is a well-established signature of photoinduced free-carrier heating in metallic systems. The transient THz photoconductivity amplitude is directly linked to the broadening of the electronic distribution and therefore serves as a sensitive proxy for the electronic temperature. For this reason, the peak amplitude of $\Delta E/E$ provides a more direct and experimentally robust measure of $T_{e,peak}$ and its fluence dependence than extracting an effective τ_{e-ph}, particularly when only modest variations are anticipated.

To exclude any contributions from direct photoexcitation of Mo_3S_7 nanoclusters, we examined the fluence-dependent OPTP response under 800 nm (1.55 eV) excitation, where Mo_3S_7 exhibits negligible absorption, as discussed in detail in our response to your **Q3**. **Fig. R6** further shows the maximum transient THz photoconductivity amplitude $\Delta E/E$, extracted at ~0.3 ps, as a function of pump fluence. At low excitation densities ($I_0 < 0.8 \times 10^{15}$ photons cm^{-2}), $\Delta E/E$ increases linearly with fluence for both samples, consistent with a monotonic rise in electronic temperature. Notably, the slope of this linear dependence is clearly smaller for Mo_3S_7-$Ti_3C_2T_x$ than for pristine $Ti_3C_2T_x$, indicating that $T_{e,peak}$ is less

sensitive to excitation fluence in the hybrid system. The fluence-dependent difference $\Delta(\Delta E/E)$ further highlights the cumulative suppression of electronic heating at higher excitation densities. This reduced fluence sensitivity is fully consistent with an ultrafast interfacial energy or carrier transfer pathway that limits electronic temperature buildup in $Ti_3C_2T_x$ by rapidly redistributing energy across the interface. The corresponding discussion has been added to the revised manuscript (**Page 14**, **Paragraph 1**).

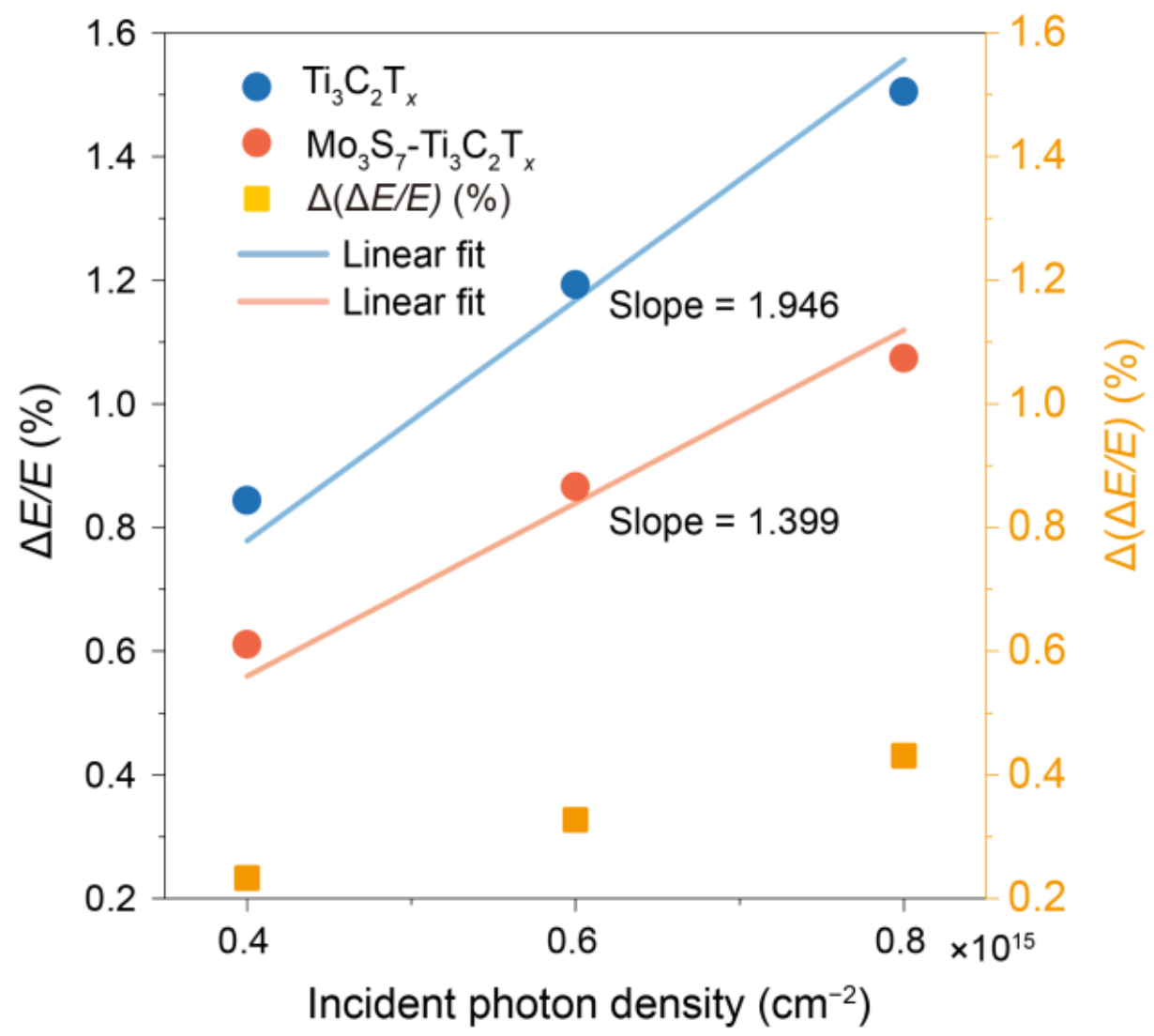

Fig. R6 Pump-fluence dependence of the transient THz photoconductivity. Peak transient THz photoconductivity amplitude ($\Delta E/E$) (at 0.3 ps) as a function of incident photon density for pristine $Ti_3C_2T_x$ and Mo_3S_7-$Ti_3C_2T_x$. The smaller slope observed for Mo_3S_7-$Ti_3C_2T_x$ indicates reduced sensitivity of the peak electronic temperature ($T_{e,peak}$) to excitation fluence, consistent with interfacial energy and/or carrier transfer. Yellow squares show the fluence-dependent difference $\Delta(\Delta E/E)$ between the two samples, highlighting the progressively stronger suppression of electronic heating in the hybrid system at higher excitation densities.

Q5. The authors mention that the incident photon fluence used for the OPTP measurements in Fig. 5c and d is the same for both MXene and MXene–Mo_3S_7 samples. However, are their absorption intensities also identical? In Fig. 3a and Supplementary Fig. 11, the absorption spectra are presented in arbitrary units, so the absorption intensities of MXene and MXene–Mo_3S_7 samples are unclear. It is recommended that the authors provide steady-state absorption spectra in the Supplementary Information, expressed in optical density (OD) units, to allow for an accurate comparison.

Response: We appreciate the insightful comment of the reviewer. In fact, the photoinduced conductivity changes in our OPTP analysis were normalized to the absorbed photon density (N_{abs}), rather than to the incident photon fluence. N_{abs} for each sample was determined from the absolute absorption at the

excitation wavelength, extracted from steady-state absorption spectra measured under identical experimental conditions, including the same substrate, comparable thickness, and identical measurement geometry. After normalization to N_{abs}, the absorbed photon densities at the excitation wavelengths are comparable for the $Ti_3C_2T_x$ and Mo_3S_7-$Ti_3C_2T_x$ samples used in the OPTP experiments. Therefore, the differences observed in the OPTP dynamics originate from intrinsic variations in ultrafast carrier and energy dissipation processes induced by nanocluster anchoring, rather than from trivial differences in optical absorption. To improve transparency and enable direct comparison, the steady-state absorption spectra expressed in optical density (OD) units (**Fig. R7**) are provided in the revised manuscript (**Supplementary Fig. 15**). By contrast, the spectra presented in **Fig. 3a** are intentionally shown in normalized (arbitrary) units to emphasize differences in spectral shape and to highlight the influence of Mo_3S_7 on the optical response.

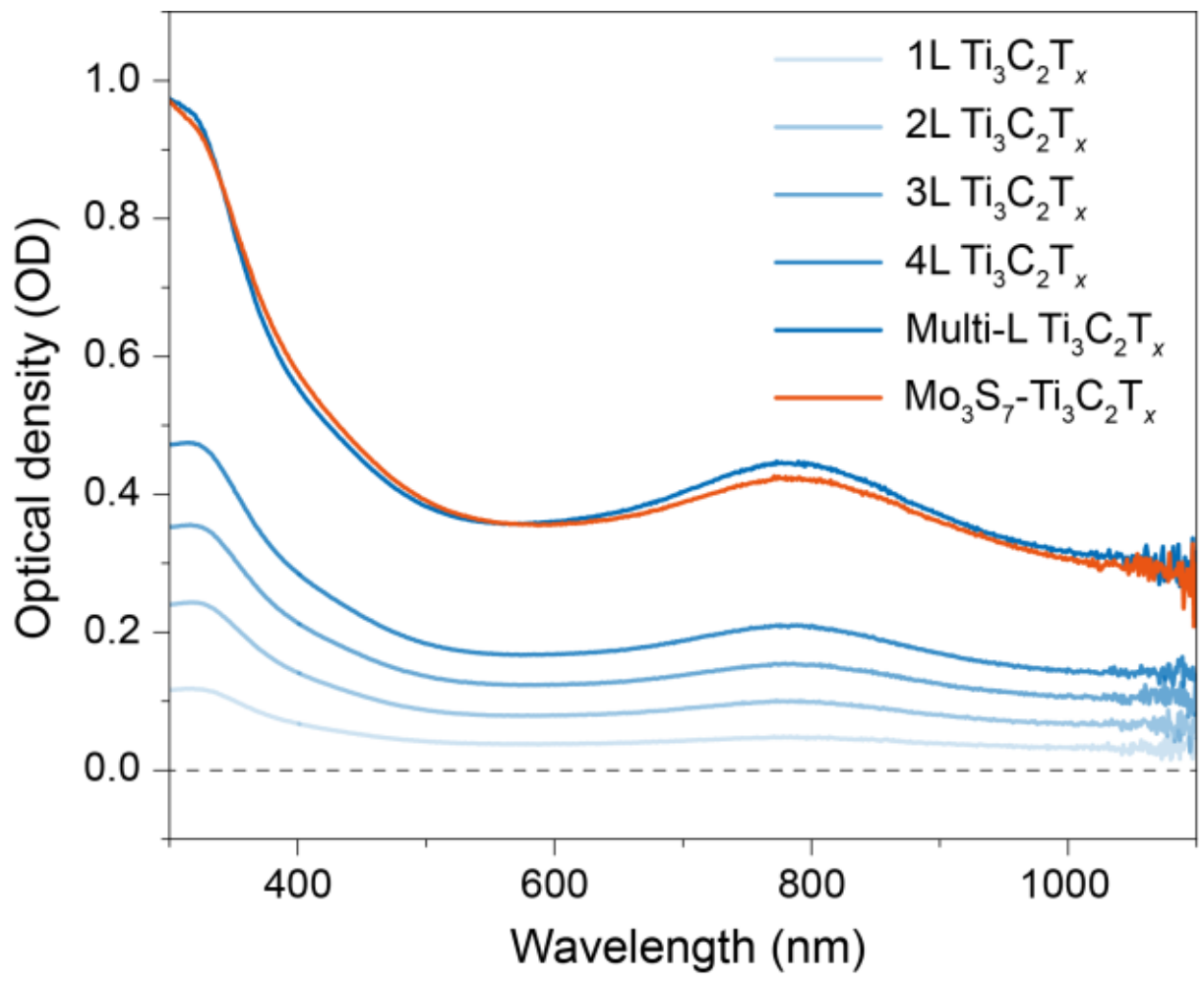

Fig. R7 Layer-dependent UV–Vis optical density (OD) spectra of assembled $Ti_3C_2T_x$ films (as references), along with those of $Ti_3C_2T_x$ and Mo_3S_7-$Ti_3C_2T_x$ samples used in the OPTP measurements, revealing comparable thicknesses of approximately 8.5 layers for both $Ti_3C_2T_x$ and Mo_3S_7-$Ti_3C_2T_x$ samples. These OD spectra were used to determine the absorbed photon density (N_{abs}) at the pump wavelengths.

To Reviewer 2:

The manuscript investigates the modulation of nonequilibrium electron–phonon (e–ph) interactions in $Ti_3C_2T_x$ MXenes through surface-anchored Mo_3S_7 nanoclusters using ultrafast transient absorption, coherent phonon spectroscopy, and THz photoconductivity. The topic is timely and potentially impactful. However, several central issues remain unresolved. The mechanistic interpretation is insufficiently supported at the microscopic level, key connections to established nonthermal-electron relaxation physics in MXenes are missing, and alternative explanations for several observations are not adequately ruled out. Substantial revision would be required for publication in Nature Communications.

Response: We appreciate the positive evaluation and constructive feedback from the reviewer, which is invaluable in improving the quality of our manuscript. All the concerns raised have been addressed through additional experiments and discussions. Details can be seen in the point-by-point responses below.

Major comments:

1. The central conclusion—that Mo_3S_7 nanoclusters competitively deplete nonthermal carriers on sub-100 fs timescales—relies solely on correlations between spectral attenuation and nanocluster anchoring. No quantitative energy-flow analysis or interfacial coupling estimates are provided to demonstrate that this channel genuinely competes with the intrinsic A_1g-mediated relaxation.

Response: We thank the reviewer for this thoughtful comment. We agree that a fully quantitative energy-flow analysis, including explicit interfacial coupling constants, would enable a direct comparison of absolute energy dissipation rates between the Mo_3S_7-mediated pathway and the intrinsic A_{1g}-mediated relaxation. We acknowledge that such a rigorous quantitative partitioning of competing channels would require detailed modeling of interfacial coupling strengths and energy redistribution, which is beyond the temporal and experimental resolution of the present work. Accordingly, the term 'competitive' is used here in a kinetic sense, referring to parallel ultrafast relaxation pathways operating on comparable sub-100 fs timescales, rather than implying a strict quantitative comparison of absolute energy fluxes. Nevertheless, we believe that the combined OPTP and TA results provide robust time-resolved constraints that allow the competition to be established qualitatively and semi-quantitatively.

Previous studies on $Ti_3C_2T_x$ have shown that nonthermal electrons can directly couple to the A_{1g} phonon mode before full electron–electron (e–e) thermalization.[3] In particular, the comparable timescales of e–e scattering (~50 fs) and coherent A_{1g} phonon formation (~60–70 fs) indicate that a significant fraction of nonthermal carriers can participate in intrinsic phonon-mediated relaxation during the early-time

nonthermal regime. This defines a well-established temporal window in which A_{1g}-mediated dissipation competes with any additional ultrafast channels.

In metallic and semi-metallic systems, a positive $\Delta E/E$ signal (i.e., negative photoconductivity response) is a well-established signature of photoinduced free-carrier heating.[4] After normalization to absorbed photon density, the peak transient THz photoconductivity amplitude provides a reliable proxy for the peak electronic temperature ($T_{e,peak}$), as it directly reflects the degree of electronic distribution broadening.[5, 6] Because $T_{e,peak}$ represents the fraction of absorbed optical energy retained in the thermalized electronic subsystem, a reduction in the peak THz photoconductivity amplitude indicates that a portion of the initially excited electronic energy is removed before complete electronic thermalization (~125 fs).[7, 8]

In the present system, the systematic suppression of the peak THz photoconductivity amplitude in Mo_3S_7-$Ti_3C_2T_x$ demonstrates a lower $T_{e,peak}$ relative to pristine $Ti_3C_2T_x$. This observation implies that an additional ultrafast relaxation pathway extracts electronic energy on sub-100 fs timescales. Such early-time energy removal necessarily reduces the population of nonthermal electrons available to drive intrinsic A_{1g}-mediated relaxation. Consistently, transient absorption measurements reveal a pronounced suppression of the coherent A_{1g} phonon amplitude upon Mo_3S_7 anchoring.

Together, these time-resolved constraints establish that the Mo_3S_7-mediated interfacial pathway operates within the same early-time nonthermal window as intrinsic A_{1g} coupling and diverts a non-negligible fraction of electronic energy before phonon-driven relaxation proceeds. In this clearly defined kinetic sense, the interfacial channel genuinely competes with intrinsic A_{1g}-mediated relaxation, even though a fully quantitative decomposition of energy flow lies beyond the scope of the present experiments. The corresponding clarification has been added into the revised manuscript (**Page 10, Paragraph 2**).

2. The reduced PIA amplitude may also arise from changes in transition dipole moments, local dielectric screening, Mo_3S_7 state filling, or interfacial hybridization. Without state-resolved probes (e.g., TR-XPS, TR-EELS, mid-IR signatures of interfacial charge), the assignment of PIA attenuation to carrier extraction remains speculative.

Response: Thank you for this insightful comment. We fully agree that a reduced PIA amplitude cannot be uniquely attributed to carrier extraction, and it may also arise from changes in transition dipole moments, local dielectric screening, state filling within Mo_3S_7, or interfacial hybridization effects. However, we would like to emphasize that PIA attenuation is not treated as a standalone indicator of carrier extraction in this work. Instead, it is interpreted as one element within a set of correlated, time-

resolved observables that together constrain the underlying relaxation pathways. Specifically, the reduced PIA amplitude is accompanied by a pronounced suppression of coherent A_{1g} phonon generation in the IBT-dominated probe region, a systematic reduction in the peak amplitude and accelerated recovery of the transient THz photoconductivity in OPTP measurements, and a clear excitation-wavelength dependence that reflects the efficiency of nonthermal carrier generation rather than steady-state absorption. Taken together, these amplitude, dynamical, and wavelength-dependent trends are consistent with a scenario in which a fraction of the initially excited nonthermal electronic energy is removed from the MXene subsystem on ultrafast timescales, prior to full electronic thermalization. Within this early-time window, such energy removal naturally reduces both the population of free carriers contributing to PIA and the nonthermal electrons available to drive coherent phonon generation.

Within this framework, alternative mechanisms such as dielectric screening, interfacial hybridization, or Mo_3S_7 state filling may indeed contribute to the observed PIA modulation. However, these effects alone cannot readily account for the simultaneous suppression of coherent phonon amplitude, the reduced peak electronic temperature, and the consistent excitation-wavelength dependence observed across both TA and OPTP measurements. We therefore treat the observed PIA attenuation as supporting, but not definitive, evidence for an effective ultrafast interfacial energy and/or carrier harvesting pathway. The manuscript has been revised to clarify this interpretation explicitly and to avoid presenting PIA attenuation as direct proof of a specific microscopic transfer mechanism (**Page 10**, **Paragraph 2**).

3. The π-phase shift and amplitude node around 580 nm are ascribed to IBT–SPR interference, yet no supporting modeling (e.g., vibronic modulation analysis or deformation-potential calculations) is presented. The observed behavior could also result from lattice softening, increased interlayer spacing, or modified plasmonic fields following Mo_3S_7 binding. These possibilities are not discussed.

Response: We appreciate the constructive comment of the reviewer. We agree that, in the absence of explicit quantitative modeling, the observed π-phase shift and amplitude node around ~580 nm cannot be uniquely assigned to a single microscopic mechanism. Accordingly, we clarify that our interpretation is physically motivated and spectroscopically constrained, rather than a definitive microscopic attribution. In the original manuscript, π-phase shift and amplitude node near 580 nm were tentatively attributed to spectral interference between IBT and SP contributions, based on their crossover in the steady-state absorption spectrum. To provide a clearer rationale, we now frame this interpretation within the established picture of coherent phonon modulation of optical resonances. In MXenes, coherent phonons are predominantly generated through a displacive excitation of coherent phonons (DECP) mechanism,[3] leading to a time-dependent lattice displacement $Q(t)$. This displacement modulates the electronic structure and optical transition energies, producing an oscillatory transient absorption

component that can be approximated to first order as **equation (R1)**, where $\Delta A(\omega,t)$ is the time-dependent change in the observable A at angular frequency ω, $A(\omega)$ is the equilibrium (unperturbed) value of the observable as a function of angular frequency, $\partial A(\omega)/\partial Q$ is the partial derivative describing how sensitive A is to the generalized coordinate Q, $Q(t)$ is the time-dependent generalized coordinate (small displacement from equilibrium), ω is the angular frequency, and t is time. This relation emphasizes that the amplitude and phase of the oscillatory signal are governed not only by the phonon coordinate itself, but critically by the spectral sensitivity of the optical response to lattice distortion.

$$\Delta A\,(\omega,t) \approx \left(\frac{\partial A(\omega)}{\partial Q}\right) Q(t) \qquad \textbf{(R1)}$$

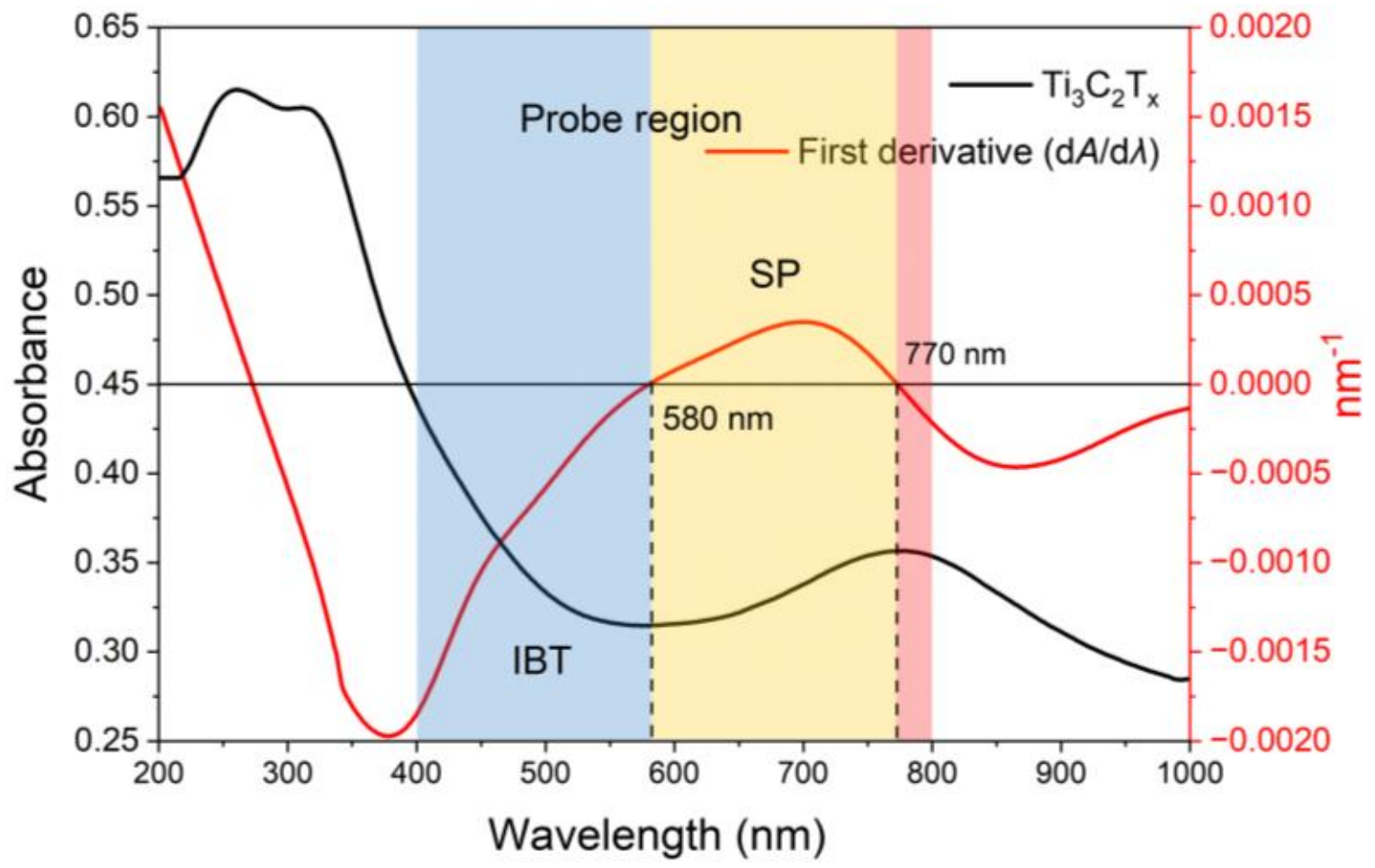

Fig. R8 Steady-state absorption spectrum of $Ti_3C_2T_x$ (A) and its first derivative with respect to wavelength ($dA/d\lambda$), which reflects the spectral sensitivity of the optical response to small perturbations of the resonance energy.

To evaluate this sensitivity spectrally, we performed a first-derivative analysis of the steady-state absorption spectrum of $Ti_3C_2T_x$ with respect to wavelength, $dA/d\lambda$ (**Fig. R8**). The derivative spectrum exhibits zero crossings near ~580 nm and ~770 nm. In particular, $dA/d\lambda$ is negative in the 400–580 nm region, which is dominated by IBT contributions, and positive in the 580–770 nm region, where the SP contribution becomes increasingly significant. This sign reversal implies that a small lattice-induced shift of the optical resonance would produce oscillatory absorption changes with opposite signs on either side of the crossover wavelength, naturally giving rise to an amplitude node and a π-phase inversion. Moreover, the larger magnitude of $|dA/d\lambda|$ in the IBT-dominated region indicates greater sensitivity of IBT-related transitions to lattice modulation. These characteristics closely match the experimental observation of an amplitude minimum near ~580 nm accompanied by a π-phase shift. We therefore interpret the phase inversion as arising from coherent phonon-induced modulation of overlapping IBT

and SP contributions. When Mo_3S_7 nanoclusters are anchored, the suppression of coherent phonon generation predominantly affects the IBT-sensitive region, while the SP-dominated region, governed by collective free-carrier oscillations, is comparatively less sensitive.

We acknowledge that Mo_3S_7 anchoring may also induce lattice softening, subtle structural changes, or modifications of local plasmonic fields. While such effects cannot be fully excluded, they are not expected, in isolation, to produce a sharply localized amplitude node with a π-phase shift at a specific probe wavelength without the presence of spectrally overlapping resonances exhibiting opposite modulation sensitivities. The IBT-SP crossover therefore provides a physically consistent and spectroscopically constrained explanation for the observed behavior. We have added the corresponding discussion to the revised manuscript (**Supplementary Fig. 13**).

4. The manuscript does not distinguish between electron transfer into Mo_3S_7 acceptor states, nonradiative plasmon-to-cluster energy transfer, phonon-mediated heat flow, or interfacial field quenching. Since no direct evidence of charge-separated species is provided, it remains unclear whether electrons, energy, or both are being transferred.

Response: We thank the reviewer for this insightful comment. Indeed, based on the present measurements, it is not possible to unambiguously distinguish among all the mechanisms mentioned. In principle, several of these mechanisms could contribute to the ultrafast removal of photoexcited electronic energy from the $Ti_3C_2T_x$ subsystem. Nevertheless, when interpreted in the context of recent ultrafast spectroscopy studies on $Ti_3C_2T_x$-based interfaces, the dominant pathways can be reasonably constrained based on their characteristic timescales and distinct experimental signatures. In particular, Zhang *et al.* demonstrated that within the nonthermal electron regime (<125 fs), multiple interfacial dissipation channels can coexist.[7] Immediately following photoexcitation, direct transfer of nonthermalized electrons into interfacial acceptor states and nonthermal-electron-driven interfacial energy transfer can occur concurrently, with relative contributions governed by excitation energy and band alignment. Importantly, these processes are intrinsically coupled in the early-time regime: nonthermal electron transfer can occur on ~50 fs timescales, while strong interfacial electronic scattering and associated energy dissipation proceed on comparable sub-100 fs timescales.

In this work, the temporal resolution of our measurements (~100 fs) does not allow us to experimentally disentangle these two ultrafast channels. However, the systematic reduction of the peak THz photoconductivity amplitude, together with its clear excitation-energy dependence, demonstrates that a substantial fraction of electronic energy is removed from the $Ti_3C_2T_x$ electronic subsystem before full electronic thermalization is completed. This behavior is consistent with ultrafast interfacial dissipation

operating within the nonthermal regime and is compatible with the coexistence of nonthermal electron transfer and electronic energy transfer to Mo_3S_7.

Phonon-mediated heat flow represents a distinct and temporally separable pathway. Such processes typically rely on acoustic phonon coupling and manifest on picosecond timescales, as previously observed in $Ti_3C_2T_x$-based hybrid systems, including $Ti_3C_2T_x$/dye interfaces[7] and $Ti_3C_2T_x/MoS_2$ heterostructures[9]. In our measurements, we also observe slower THz photoconductivity evolution in the 2–8 ps window, which we attribute to phonon-mediated interfacial heat transfer. This slower component is therefore distinguished from the sub-100 fs suppression of peak electronic temperature that governs the early-time competition with coherent phonon generation.

Additionally, interfacial field quenching, which would primarily modify the optical or THz response through static or transient electric fields without real charge or energy transfer, does not readily account for the combined experimental observations. In particular, it cannot naturally explain the excitation energy-dependent suppression of THz photoconductivity, the accelerated recovery dynamics, or the systematic reduction of coherent A_{1g} phonon amplitude observed across both OPTP and TA measurements. These signatures are more consistent with genuine ultrafast electronic energy dissipation rather than a purely field-induced modulation of the probe response.

To avoid confusion, we have modified our title as "*Modulating Nonequilibrium Electron–Phonon Interactions and Energy Relaxation in MXenes by Surface-Anchored Mo_3S_7 Nanoclusters*" to describe the effective interfacial dissipation mechanism introduced by Mo_3S_7 anchoring. This terminology is intended to capture the collective outcome of coupled ultrafast electron transfer and energy dissipation processes, without over-specifying their relative microscopic contributions beyond what can be resolved by the present experiments. We have added the corresponding discussion to the revised manuscript (**Page 1**, **Paragraph 1**).

5. Critical controls are missing: MXene with an inert ligand of similar size to Mo_3S_7 (to decouple steric vs electronic effects), varying nanocluster coverage to show scaling of extraction rate, different terminations (F, OH, O) to test the dependence on surface electronic structure. Without these, it is difficult to attribute the observed modulation strictly to the Mo_3S_7–$Ti_3C_2T_x$ interaction.

Response: We thank the reviewer for the valuable suggestion. To address to the concern raised, we performed additional OPTP measurements on $Ti_3C_2T_x$ with varied Mo_3S_7 loading densities. Importantly, these measurements were conducted under 800 nm (1.55 eV) excitation, at which Mo_3S_7 exhibits negligible absorption, thereby excluding direct nanocluster photoexcitation and allowing us to isolate

interfacial effects. The results reveal a clear and monotonic decrease in the peak THz photoconductivity amplitude with increasing Mo_3S_7 coverage (**Fig. R9a**). Furthermore, after normalization of the transient photoconductivity traces, the decay dynamics progressively accelerate as the Mo_3S_7 loading increases (**Fig. R9b**). This systematic scaling behavior provides strong evidence that the modulation of carrier dynamics is governed by interfacial interaction strength rather than by a generic perturbation of the MXene lattice or morphology.

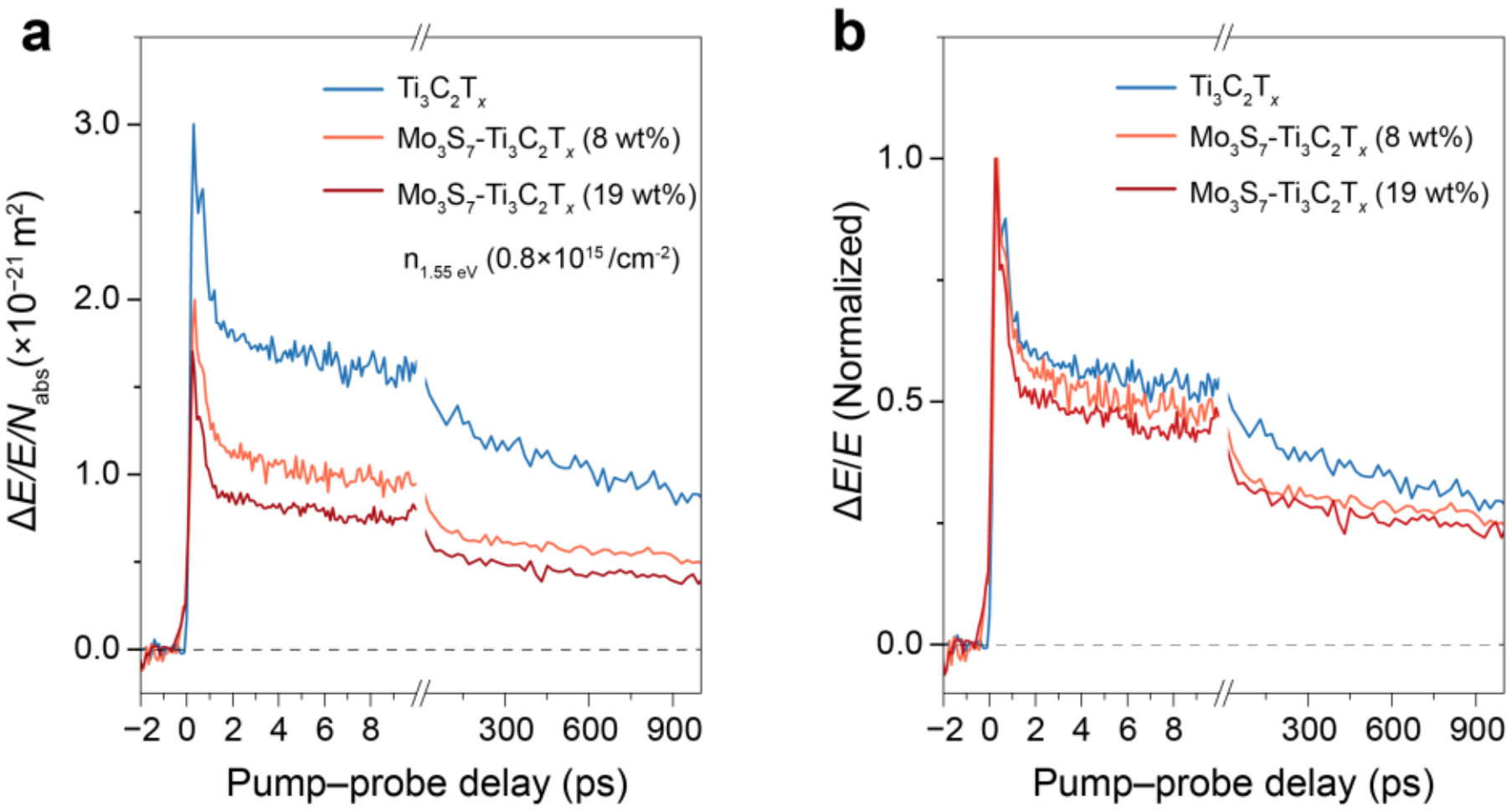

Fig. R9 Mo_3S_7-coverage-dependent THz photoconductivity dynamics of Mo_3S_7-$Ti_3C_2T_x$ under 800 nm (1.55eV) excitation **a**, without and **b**, with normalization.

We acknowledge that additional controls, such as incorporating inert ligands of comparable size or systematically varying surface terminations (–F, –OH, –O), would further strengthen mechanistic interpretation. However, due to the intrinsic chemical complexity of MXene surfaces and the experimental difficulty of independently tuning termination chemistry while maintaining comparable structural and optical properties, a comprehensive exploration of all such variables lies beyond the scope of the present study. The corresponding discussion has been added to the revised manuscript (**Page 14, Paragraph 1**).

6. The negative photoconductivity in the OPTP measurements is attributed to enhanced extraction efficiency, but similar signals can arise from mobility loss, increased scattering, interfacial disorder, or changes in Drude weight. Frequency-resolved Drude–Smith analysis is needed to distinguish these effects and to justify the reported extraction efficiencies.

Response: We fully agree that a suppressed negative photoconductivity observed in OPTP measurements can, in principle, originate from multiple mechanisms in addition to interfacial charge/energy extraction. In pristine $Ti_3C_2T_x$, the negative transient THz photoconductivity is consistent

with photoinduced carrier heating, a well-established response in metallic systems, where the increased electron-impurity scattering reduces conductivity without significantly changing carrier density. At the same time, a transiently increased electronic temperature indicates the enhanced e–e and e–ph scattering. In contrast, the pronounced suppression of the negative THz photoconductivity observed in Mo_3S_7-$Ti_3C_2T_x$ is attributed to ultrafast removal of electronic energy from $Ti_3C_2T_x$ via interfacial pathways. This interpretation is supported by the correlated observations of excitation-wavelength-dependent suppression of the THz photoconductivity amplitude, systematically accelerated recovery dynamics in the hybrid sample, and reduced sensitivity of the peak electronic temperature ($T_{e,peak}$) to pump fluence in the presence of Mo_3S_7 nanoclusters.

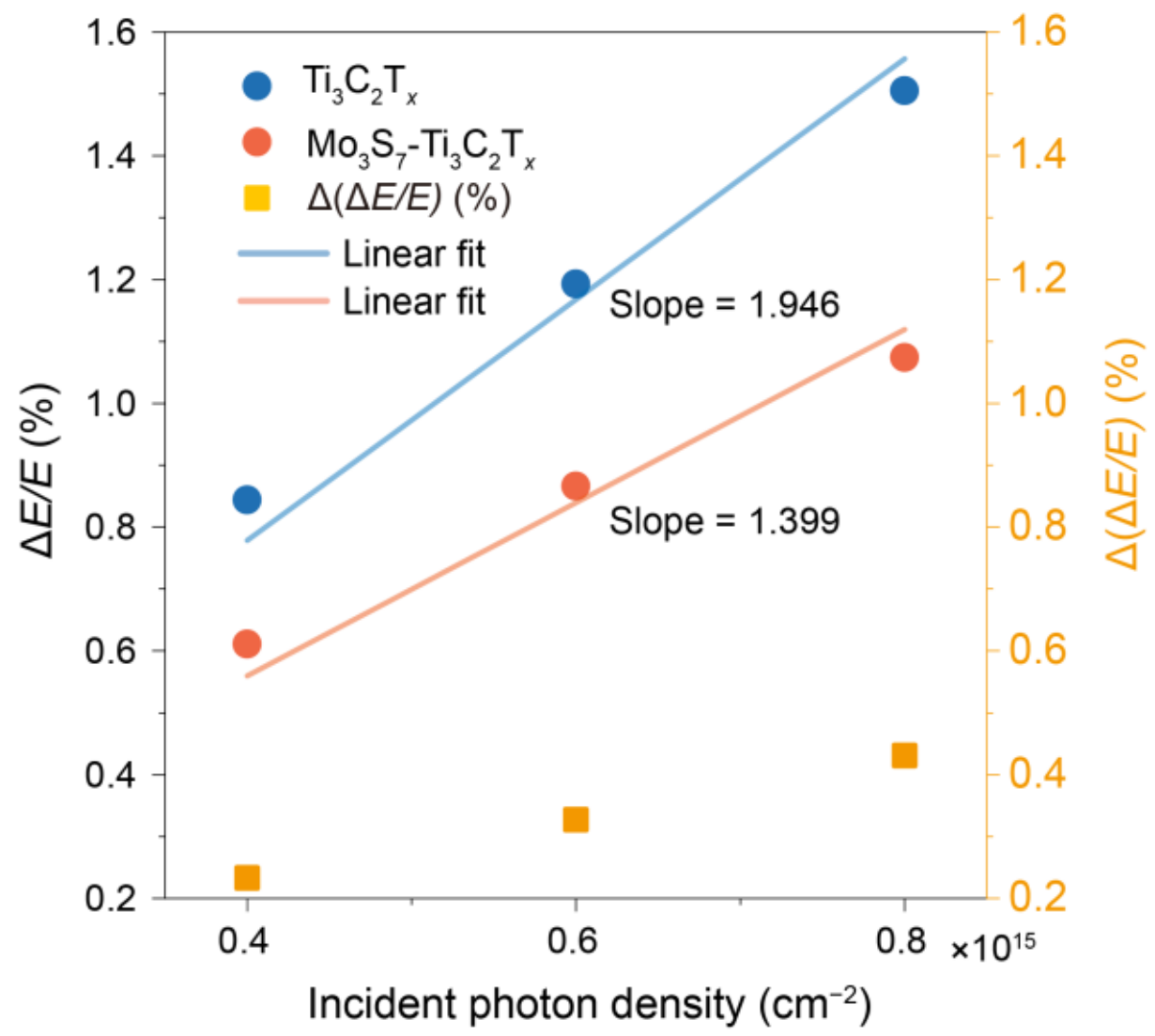

Fig. R10 Pump-fluence dependence of the transient THz photoconductivity. Peak transient THz photoconductivity amplitude ($\Delta E/E$) (at 0.3 ps) as a function of incident photon density for pristine $Ti_3C_2T_x$ and Mo_3S_7–$Ti_3C_2T_x$. The smaller slope observed for Mo_3S_7–$Ti_3C_2T_x$ indicates a reduced sensitivity of the $T_{e,peak}$ to excitation fluence, consistent with interfacial energy and/or carrier transfer.

As reported previously, ultrafast interfacial extraction of hot carriers can effectively limit the peak electronic temperature ($T_{e,peak}$) of the photoexcited electron gas.[10] A reduced $T_{e,peak}$ modifies subsequent relaxation dynamics and weakens the fluence dependence of e–ph coupling. In our OPTP experiments, the peak amplitude of $\Delta E/E$ offers a more direct measure of $T_{e,peak}$ and its fluence dependence than extracting an effective $\tau_{e\text{–}ph}$. To exclude contributions from direct photoexcitation of Mo_3S_7 nanoclusters, we examined the fluence-dependent OPTP response under 800 nm (1.55 eV) excitation. **Fig. R10** presents the maximum transient THz photoconductivity amplitude ($\Delta E/E$) as a function of pump fluence. At low excitation densities ($I_0 < 0.8 \times 10^{15}$ photons cm^{-2}), $\Delta E/E$ increases linearly with fluence for both samples, consistent with a monotonic rise in electronic temperature. Notably, the slope of this linear

dependence is smaller for Mo_3S_7-$Ti_3C_2T_x$ than for pristine $Ti_3C_2T_x$, indicating that $T_{e,peak}$ is less sensitive to excitation fluence in the hybrid system. The fluence-dependent difference $\Delta(\Delta E/E)$ further highlights the cumulative suppression of electronic heating at higher excitation densities. This behavior is consistent with the interfacial energy or carrier transfer mechanism in the previous report,[10] where limiting the electronic temperature rise leads to a reduced fluence dependence of e–ph relaxation dynamics. The corresponding discussion has been added to the revised manuscript (**Page 14**, **Paragraph 1**).

In addition, steady-state transport measurements provide additional evidence for interfacial electronic interactions in Mo_3S_7-$Ti_3C_2T_x$ (**Fig. R11**). Temperature-dependent resistivity analysis reveals a crossover from two-dimensional variable-range hopping transport in pristine $Ti_3C_2T_x$ to three-dimensional VRH conduction in Mo_3S_7-$Ti_3C_2T_x$, indicating enhanced interlayer electronic coupling mediated by Mo_3S_7 nanoclusters. Hall measurements further show increased carrier concentration and mobility, while the Seebeck coefficient exhibits an increased magnitude upon Mo_3S_7 incorporation, suggesting modifications of the electronic structure near the Fermi level. These steady-state transport results support the presence of strong interfacial electronic coupling in the hybrid system. The corresponding discussion has been added to the revised manuscript (**Page 16**, **Paragraph 2**).

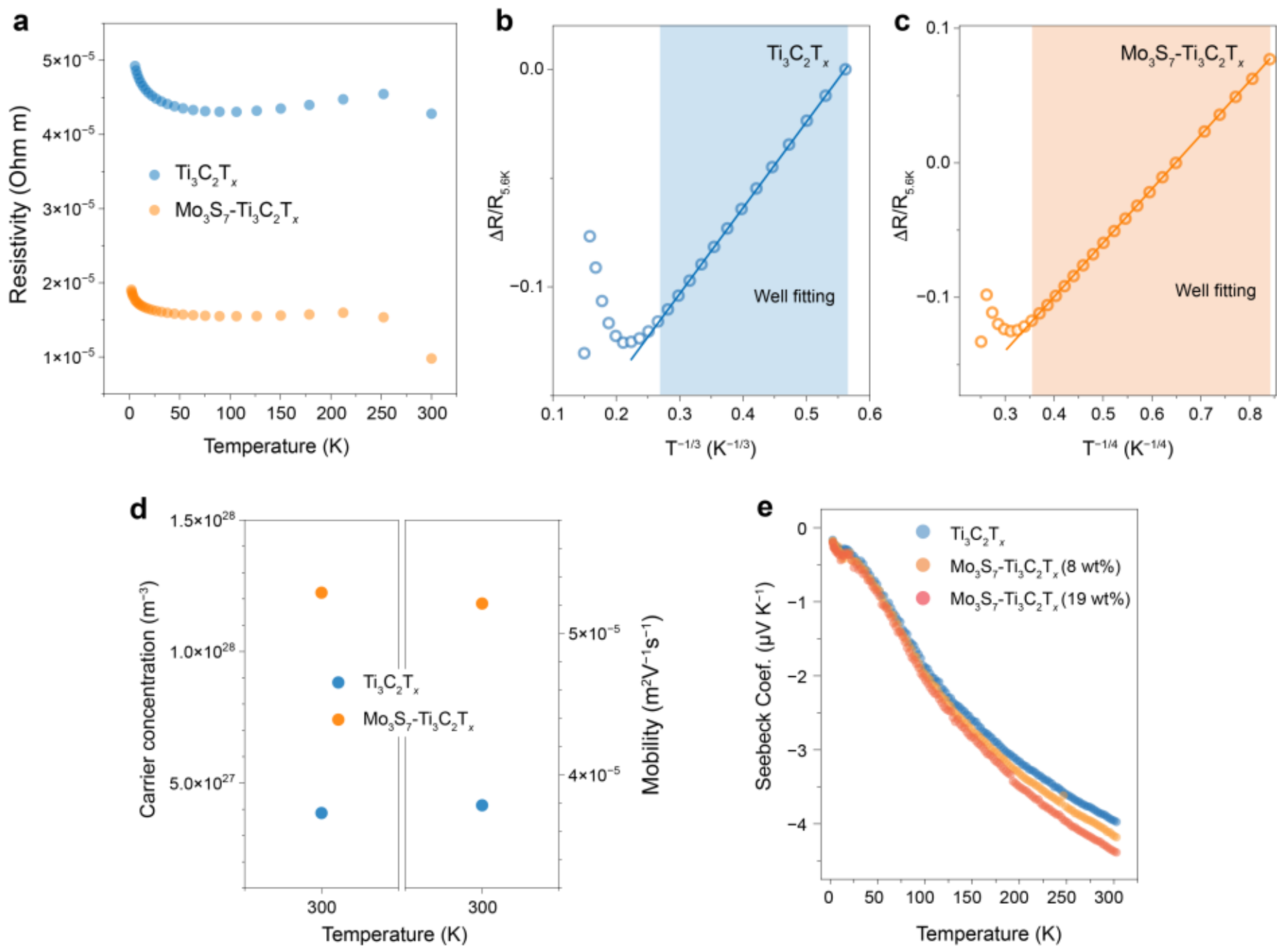

Fig. R11 Electric and thermoelectric transport properties of Mo_3S_7-$Ti_3C_2T_x$. a Temperature-dependent resistivity. **b** Normalized resistance vs. $T^{-1/3}$ for $Ti_3C_2T_x$, showing linear behavior below ~100 K characteristic of two-dimensional variable-range hopping. **c** Normalized resistance vs. $T^{-1/4}$ for

Mo_3S_7-$Ti_3C_2T_x$, indicating three-dimensional variable-range hopping. **d** Hall carrier concentration (electrons) and Hall mobility at 300 K for $Ti_3C_2T_x$ and Mo_3S_7-$Ti_3C_2T_x$. **e** Seebeck coefficient of $Ti_3C_2T_x$ and Mo_3S_7-$Ti_3C_2T_x$ with different cluster loadings, showing the incremental influence of Mo_3S_7 on thermoelectric effect.

Therefore, mechanisms such as static disorder, mobility-limited scattering increase, or changes in Drude weight do not naturally explain the combined amplitude, dynamics, and wavelength-dependent suppression observed in our experiments. These mechanisms would primarily affect THz transport, whereas we independently observe a pronounced suppression of coherent A_{1g} phonon generation in the TA measurements. Because TA directly probes ultrafast electronic and lattice dynamics and is insensitive to THz transport, these observations indicate a genuine modification of the early-time electronic energy landscape rather than a simple change in carrier mobility. While frequency-resolved Drude-Smith analysis can be informative under quasi-steady-state conditions, in highly metallic $Ti_3C_2T_x$ under intense ultrafast photoexcitation, the early-time THz response is dominated by strongly non-equilibrium carrier distributions and rapid e–e scattering on sub-100 fs timescales. Under such conditions, the assumptions of the Drude-Smith formalism are not strictly satisfied, and the analysis would not uniquely disentangle carrier density, scattering rate, and effective mass in the ultrafast regime. Therefore, although a full frequency-resolved transport analysis could be informative in future studies, the present OPTP results, together with complementary TA measurements, consistently support the conclusion that Mo_3S_7 nanoclusters introduce an additional ultrafast interfacial energy and/or carrier extraction pathway that suppresses electronic heating and competes with intrinsic relaxation processes in $Ti_3C_2T_x$.

7. The assumption of a uniformly O-terminated MXene with clean Mo–O–Ti coordination is unrealistic. Mixed O/OH/F termination and hydration layers can strongly affect interfacial energetics and hot-carrier pathways A statistically meaningful surface-termination analysis (ToF-SIMS, STEM-EELS, or quantitative XPS peak fitting) is required to justify the structural model.

Response: We appreciate the constructive comment of the reviewer. Indeed, $Ti_3C_2T_x$ surfaces are chemically heterogeneous rather than uniformly O-terminated. Such complexity can potentially influence interfacial energetics and hot-carrier transport pathways. To provide a statistically meaningful basis for the structural model, we performed quantitative XPS peak-fitting analysis to determine the surface termination composition of $Ti_3C_2T_x$ before and after Mo_3S_7 anchoring (**Table R1**). Deconvolution of the O 1s spectra reveals that pristine $Ti_3C_2T_x$ exhibits a mixed termination environment with an estimated O/OH/F ratio of approximately 28.4%/34.2%/37.4%. Oxygen-containing terminations (O + OH) collectively account for ~63% of the surface terminations, confirming

their dominant presence even prior to functionalization. After Mo_3S_7 anchoring, the surface termination distribution evolves to approximately 26.0%/45.8%/28.2% for O/OH/F, with the fraction of oxygen-containing species increasing to ~72%. The relative increase in OH-related contributions is likely associated with enhanced hydration effects and expanded interlayer spacing upon Mo_3S_7 incorporation (**Fig. 2d**). These results confirm that the $Ti_3C_2T_x$ surface remains chemically heterogeneous both before and after functionalization, while O- and OH-related terminations dominate the interfacial chemistry in both cases.

Table R1. Statistical surface termination analysis of $Ti_3C_2T_x$ derived from quantitative XPS peak fitting.

**$Ti_3C_2T_x$**			**Mo_3S_7–$Ti_3C_2T_x$**		
O1s A (**–O**) At. %	O1s B (**–OH/H_2O**) At. %	F (**–F**) At. %	O1s A (**–O**) At. %	O1s B (**–OH/H_2O**) At. %	F (**–F**) At. %
7.82	9.39	10.29	6.80	11.99	7.38
(28.4 %)	(34.2%)	(37.4%)	(26.0%)	(45.8%)	(28.2%)

Regarding the computational model, we acknowledge that a comprehensive treatment of mixed terminations would require large configurational sampling and is beyond the scope of the present study. We emphasize that the purpose of the electronic-structure calculations here is not to quantitative model ultrafast carrier or phonon dynamics, but rather to provide a physically reasonable and internally consistent picture of the relative energetic positions and density of interband transitions in the visible spectral range. We therefore employ $Ti_3C_2O_2$ (now treated with a DFT+U framework) as a representative and widely used model for MXene simulations. This choice is justified because it provides a computationally tractable surface termination, has been extensively used in prior studies of MXene electronic and optical properties enabling direct comparison with literatures, and yields general insights into the d-manifold-dominated interband transitions that are broadly relevant across different termination chemistries. The electronic-structure calculations thus serve as qualitative support for the experimentally observed spectral features rather than as a complete statistical description of surface disorder. The corresponding discussion has been incorporated into the revised manuscript (**Page 17, Paragraph 1; Supplementary Table 1**).

8. Compared with previous reports that show weak e–ph coupling in $Ti_3C_2T_x$ (e.g., THz studies), the presented results strongly emphasize suppression of A_{1g} coherent phonons. The manuscript fails to

reconcile these discrepancies or provide a unified picture of how nanoclusters switch between strong and weak coupling regimes.

Response: Thank you for the valuable comment. We would like to clarify that these statements are not contradictory, but rather reflect fundamentally different physical regimes, timescales, and observables probed in the respective experiments. In THz transport measurements,[11] the reported weak e–ph coupling reflects quasi-equilibrium or near-steady-state carrier dynamics, where momentum relaxation and charge transport are examined after electronic thermalization. In this regime (e.g., ~200 ps after photoexcitation for $Nb_4C_3T_x$, or under steady-state conditions in $Ti_3C_2T_x$), carrier-phonon interactions are effectively described by scattering rates, long-range Fröhlich-type coupling, and large-polaron behavior, leading to weak coupling in the transport sense. By contrast, the coherent A_{1g} phonon dynamics discussed in our study and in recent ultrafast vibrational studies probe a far-from-equilibrium regime within the first few hundred femtoseconds following photoexcitation.[3] During this early-time window, nonthermal electrons couple selectively and efficiently to specific phonon modes, particularly the A_{1g} mode, leading to strong mode-selective e–ph coupling (λ = 1.62 ± 0.33 for A_{1g} mode) and efficient energy injection prior to electronic thermalization. This does not imply globally strong e–ph coupling relevant for transport, but rather a transient, non-thermal energy-flow channel operative only at ultrashort timescales. Within this unified framework, the role of Mo_3S_7 nanoclusters does not substantially alter the intrinsic e–ph coupling of $Ti_3C_2T_x$. Instead, the nanoclusters introduce an additional ultrafast interfacial dissipation pathway that operates on a comparable sub-100 fs timescale. This pathway intercepts a portion of the nonthermal electronic energy that would otherwise drive coherent A_{1g} phonon formation, thereby suppressing the e–ph interaction strength for the non-equilibrium electronic system.

9. The manuscript relies on PBE-level electronic structures, which are known to be qualitatively unreliable for MXenes: semilocal GGA severely overdelocalizes Ti-3d states, underestimates d–d splittings and interband transition energies by several hundred meV, and fails to reproduce the termination-dependent crystal-field environment that governs A_1g/Eg phonon coupling and hot-electron relaxation pathways. Given the decisive influence of these d-manifold features on e–ph dynamics, plasmonic response, and TA/PIA assignment, PBE-based interpretations are fundamentally flawed and should be replaced with at least DFT+U or hybrid-DFT benchmarks.

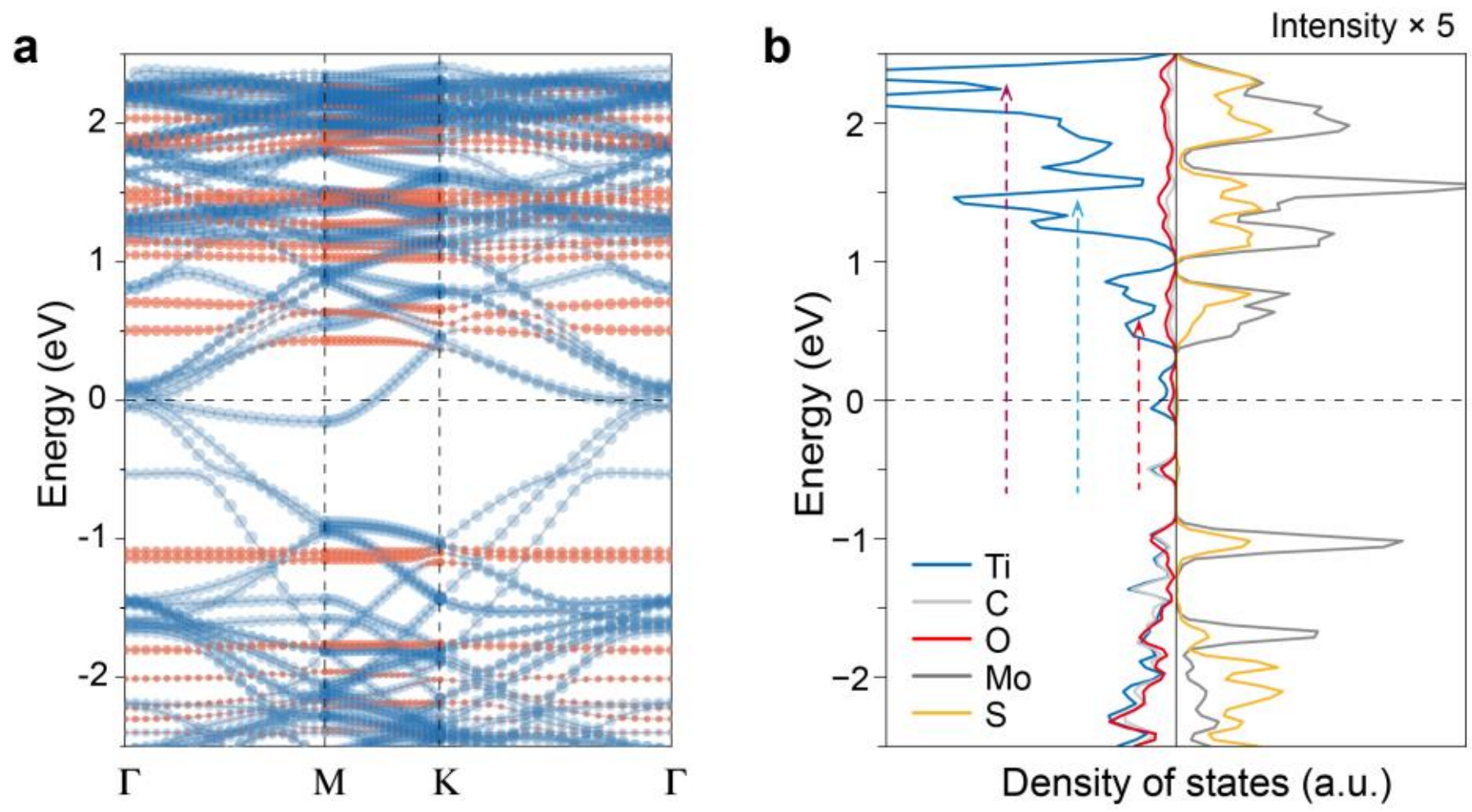

Fig. R12 a, Projected electronic band structure, and **b**, Projected density of states (DOS) of Mo_3S_7-$Ti_3C_2T_x$ with DFT+U benchmark.

Response: We thank the reviewer for this technically informed comment. To address this concern, we performed additional electronic-structure calculations for $Ti_3C_2O_2$ using the DFT+U method, which partially corrects self-interaction errors and provides a more accurate description of transition-metal d states (**Fig. R12**). Nevertheless, we would like to mention that the purpose of the electronic-structure calculations in this work is not to quantitatively model ultrafast carrier or phonon dynamics. Rather, they offer a physically reasonable and internally consistent picture of the relative energetic positions and density of interband transitions in the visible spectral range. The assignments of transient absorption and PIA features rely primarily on experimentally resolved temporal dynamics and established spectroscopic signatures, while the electronic-structure calculations serve as qualitative support for the existence and spectral distribution of dense interband-transition manifolds. We fully acknowledge that surface terminations influence the detailed electronic structure, local crystal-field environment, and interfacial dynamics of MXenes. A comprehensive treatment of mixed terminations would require large configurational sampling and lies beyond the scope of the present work, as explained in our response to your **Q7**. Besides, our main experimental conclusions, namely the assignment of PIA features to transient renormalization of pre-existing IBTs and the distinction between interband, plasmonic, and free-carrier responses based on their temporal characteristics, do not depend sensitively on termination-specific crystal-field details. Rather, they are governed by the metallic nature of MXenes, the presence of dense Ti 3d-derived interband manifolds, and their strong coupling to nonequilibrium electronic and lattice dynamics. Accordingly, we have added the corresponding discussion to the revised manuscript (**Page 17**, **Paragraph 1; Fig. 7b-c**).

Minor but Important Issues:

1. The reported "sub-100 fs extraction" is at or below the instrument response; deconvolution is insufficiently documented.

Response: We thank the reviewer for raising this important methodological concern. In our kinetic analysis, the transient dynamics were fitted using a bi-exponential decay function convoluted with a Gaussian instrument response function (IRF) with a full width at half maximum (FWHM) of 50 fs. While this temporal resolution allows us to distinguish the presence of an ultrafast relaxation component from slower processes, it does not permit a reliable extraction of its intrinsic lifetime when the process occurs on timescales comparable to or shorter than the IRF. In addition, the earliest experimentally accessible probe delay in our measurements is ~100 fs. By this time, the ultrafast relaxation process is already largely completed. Consequently, the extracted fast component is IRF-limited, and assigning a specific decay constant shorter than the instrumental resolution would not be physically meaningful. For this reason, we refer to the process as occurring on a "sub-100 fs" timescale, which should be interpreted as an upper bound rather than a quantitatively resolved lifetime. To improve clarity and transparency, we have revised the manuscript to explicitly document the convolution procedure (**Page 22**, **Paragraph 1**).

2. Spectral assignments (PIA-1/2/3) should be backed by calculations of excited-state absorption or joint DOS.

Response: We appreciate the constructive comment of the reviewer. In metallic MXenes, which possess a high density of states near the Fermi level and strongly non-equilibrium carrier distributions immediately after photoexcitation, rigorous calculations of excited-state absorption or joint density of states in the conventional semiconductor sense are technically challenging and often not uniquely interpretable, particularly in the early-time nonthermal regime probed here. In such systems, photoexcitation does not create discrete excited states but instead generates a hot-electron distribution within continuous bands. As a result, "excited-state absorption" is not well defined in the usual framework applicable to semiconductors. Instead, we adopt a combined strategy that integrates time-resolved spectroscopic signatures with established theoretical and experimental studies to rationalize the observed PIA features.

In a recent study,[12] simultaneous ultrafast transmission and reflection (SUTR) pump-probe measurements on $Ti_3C_2T_x$, enabled reconstruction of the complex refractive index (n + ik) over 450–1300 nm. That work demonstrated that the distinction between interband, plasmonic, and free-carrier responses in MXenes is governed primarily by their characteristic temporal dynamics rather than by

spectral position alone. Specifically, probe wavelengths near ~520 nm exhibited positive transient absorption assigned to pump-induced band renormalization of pre-existing interband transitions persisting beyond the e–ph thermalization. In contrast, the response near ~700 nm was confined to ultrafast e–e timescales and attributed to plasmonic excitation, while the near-infrared region (>1000 nm), dominated by free carriers, exhibited negligible transient absorption. Consistent with this framework, we assign PIA-1 and PIA-2 to pump-induced renormalization of interband transitions in $Ti_3C_2T_x$. Photoexcitation drives the electronic system far from equilibrium, leading to transient modifications of electronic self-energy, occupation distribution, and e–ph scattering rates. These effects alter the line shape and spectral weight of pre-existing interband transitions, producing positive differential absorption signals. Importantly, this assignment does not rely on the creation of new excited-state absorption channels, but rather reflects transient redistribution within the dense interband manifold intrinsic to metallic $Ti_3C_2T_x$. This interpretation is further supported by recent first-principles calculations identifying dense Ti 3d-derived interband-transition manifolds spanning the visible range,[4] underscoring the abundance of optically allowed transitions whose spectral weight can be transiently redistributed under non-equilibrium excitation.

By contrast, PIA-3 (~700 nm) exhibits distinct dynamics. It is confined to sub-200 fs timescales and evolves into a bleaching-like feature near the plasmon resonance at later delays. This behavior is characteristic of ultrafast perturbation of a collective plasmonic excitation dominated by enhanced e–e scattering immediately after photoexcitation. At early times, increased electronic scattering broadens the plasmon line shape, leading to apparent absorption enhancement on the resonance flank. As the electronic system thermalizes, this nonthermal broadening diminishes and the plasmonic response evolves into a quasi-thermal hot-electron state with reduced oscillator strength.

Within this unified picture, the strong attenuation of PIA-1 and PIA-2 upon Mo_3S_7 anchoring can be understood as suppressed band renormalization in MXene. Interfacial carrier extraction and/or energy dissipation reduces the buildup of a hot, broadened electronic distribution, thereby weakening the pump-induced modification of interband transitions. Consequently, the amplitudes of PIA-1 and PIA-2 are strongly reduced and decay more rapidly. In contrast, PIA-3 arises from ultrafast plasmon damping primarily governed by e–e scattering and is therefore comparatively less sensitive to interfacial extraction. The corresponding discussion has been added to the revised manuscript (**Page 9**, **Paragraph 2**).

3. Potential nanocluster degradation under high-energy excitation (266 nm) is not addressed.

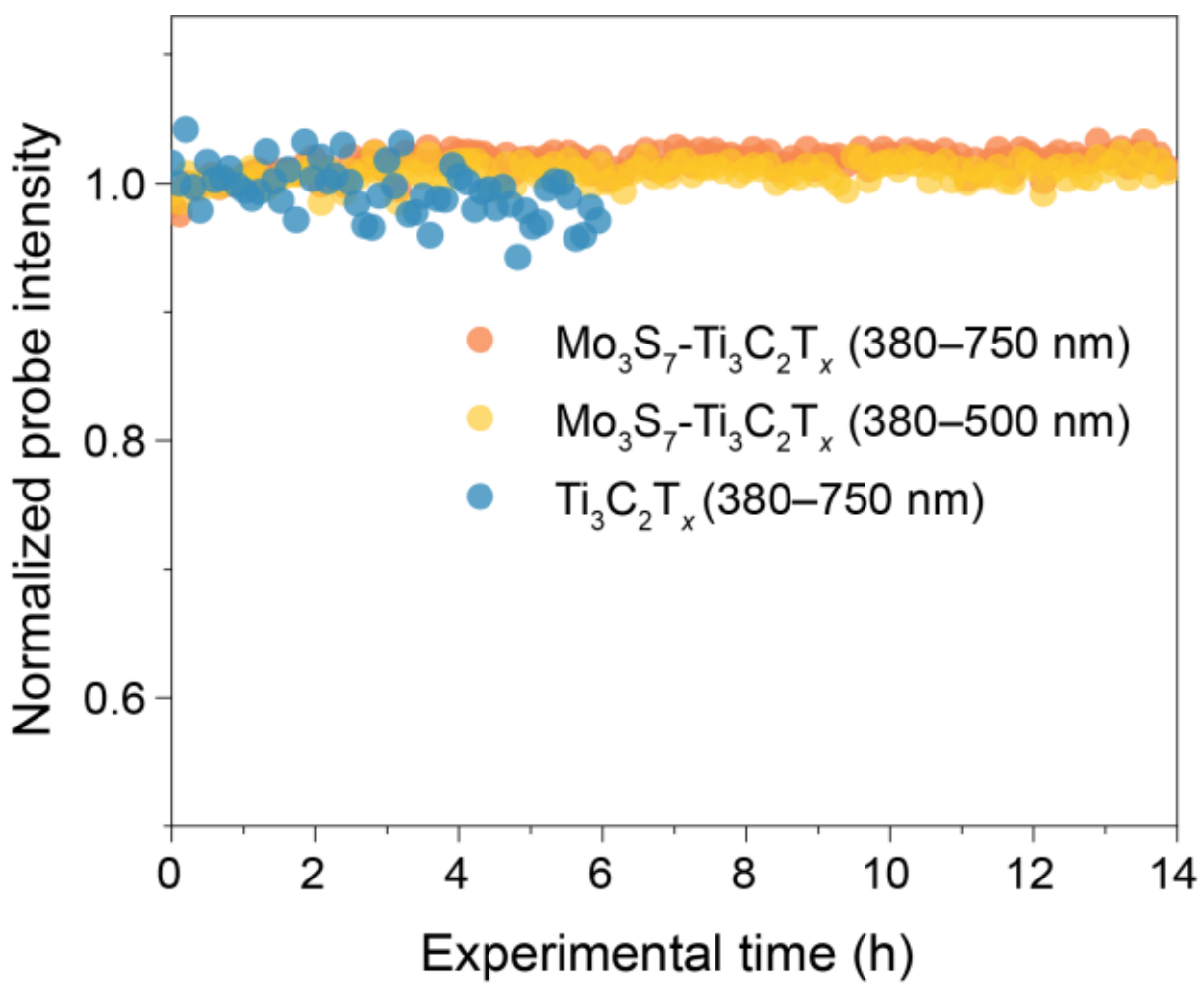

Fig. R13 Temporal evolution of the normalized probe intensity under 266 nm excitation for $Ti_3C_2T_x$ and Mo_3S_7-$Ti_3C_2T_x$, averaged over the indicated spectral ranges. The probe intensity remains constant over the entire measurement duration (~14 h), indicating no detectable sample degradation.

Response: Thank you for the valuable comment. The TA spectra presented in this work were obtained by averaging 129 consecutive scans for the Mo_3S_7-$Ti_3C_2T_x$ sample under 266 nm excitation, corresponding to a total acquisition time of approximately 14 hours. The pump fluence used in the TA measurements was 118 μJ cm^{-2}, which falls within the moderate-to-low fluence range commonly employed in ultrafast transient absorption studies and is not expected to induce photodamage under standard conditions. In ultrafast spectroscopy, sample degradation typically manifests as a progressive reduction of signal amplitude or systematic spectral changes between consecutive scans due to irreversible chemical or structural evolution. To further substantiate this concern, we monitored the temporal evolution of the averaged probe spectrum in the 380–750 nm range, which directly overlaps with the steady-state absorption band of Mo_3S_7 (**Fig. R13**). The probe spectra were normalized to their initial values to track any long-term drift. It is expected that nanocluster degradation would induce a decrease in absorption, leading to a corresponding increase in probe transmission over time. For an initial absorbance on the order of ~0.8–1.0 (optical density), even partial degradation would produce a measurable increase in probe intensity on the order of tens of percent. However, the experimentally observed probe intensity remains essentially constant throughout the entire acquisition period, with fluctuations below 1%, which are within the normal range of laser power and detector stability. No systematic increase in transmission or evolution of spectral features is detected. These observations demonstrate that no detectable degradation of Mo_3S_7 nanoclusters occurs under the applied 266 nm excitation conditions during the TA measurements. The corresponding discussion has been added to the revised manuscript (**Page 10**, **Paragraph 2; Supplementary Fig. 12**).

4. The Mo/Ti atomic ratio (0.034) suggests very low coverage; it is unclear how such sparse clustering produces the large phonon-amplitude suppression reported.

Response: We appreciate the constructive comment of the reviewer. The initially reported value of 1.7% was derived from the quantified Mo/Ti atomic ratio (0.034) obtained from elemental analysis. This value corresponds to a stoichiometric ratio calculated under two simplified assumptions: 1) O is the only surface termination species on $Ti_3C_2T_x$ (i.e., $Ti_3C_2O_2$), and 2) all O terminations are equally accessible and capable of coordinating with Mo_3S_7 nanoclusters. Therefore, the reported 1.7% represents a stoichiometric fraction rather than true geometric surface coverage. In realistic systems, the number of sterically accessible and chemically effective O coordination sites is substantially lower than the total number of O terminations. Based on the simulated geometric structural models (**Fig. R14**), we estimate that only approximately 11.1% of O terminations can function as effective coordination sites for Mo_3S_7 nanoclusters at maximum packing density. Within this framework, the Mo_3S_7 loading in our sample corresponds to 15.3% utilization of the effective O coordination sites. Furthermore, $Ti_3C_2T_x$ typically contains mixed surface terminations, including –F, –Cl, and –OH groups, which are unlikely to effectively anchor Mo_3S_7 nanoclusters. The presence of these less-coordinating terminations further reduces the number of available anchoring sites and thus lowers the realistic geometric surface coverage.

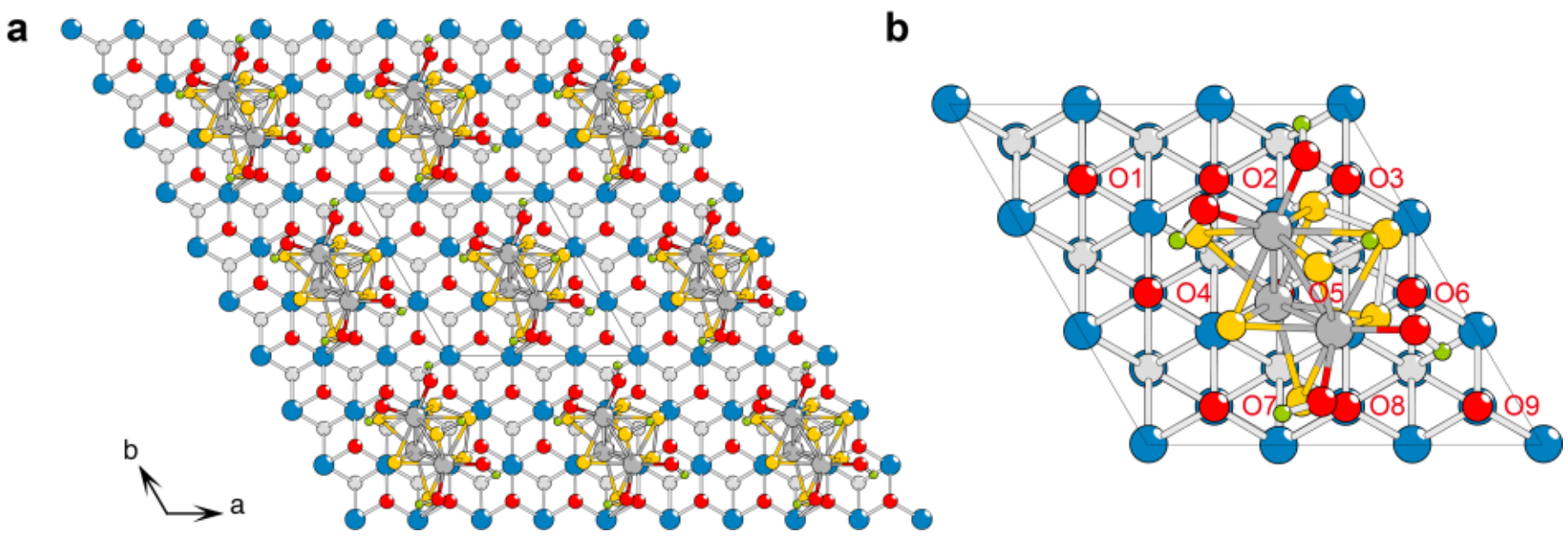

Fig. R14 a, Optimized model showing the maximum achievable packing density of Mo_3S_7 nanoclusters on $Ti_3C_2O_2$ when steric constraint is considered. **b**, All O terminations in a representative unit cell of Mo_3S_7–$Ti_3C_2O_2$, illustrating that only one-ninth of the O sites are sterically accessible and effective for coordination with Mo_3S_7 nanoclusters.

We acknowledge that describing the 1.7% stoichiometric ratio as "surface coverage" may have caused confusion, since this value does not directly represent geometric area coverage. To avoid ambiguity, we have revised the manuscript to describe the Mo_3S_7 loading primarily in terms of mass fraction (8 wt%), which provides a more intuitive and model-independent metric (**Page 6, Paragraph 1**). Although the absolute surface coverage is relatively low, we believe that this amount of Mo_3S_7 is sufficient to induce effective charge/energy transfer and pronounced modulation of electron-phonon (e-ph) interactions.

Previous studies have demonstrated that comparable levels of interfacial modification can substantially alter carrier relaxation dynamics. For example, Mo_3S_{13} cluster-functionalized TiO_2 with 1.78 wt% loading achieves near-optimal photocatalytic H_2 evolution rates of ~65 μmol h^{-1}, indicating highly efficient charge transfer.[1] In addition, 0.5 wt% Pt-loaded $Ti_3C_2T_x$ has been reported to markedly influence electron–phonon interactions via efficient interfacial charge transfer.[2] These early published results collectively demonstrate that even small amounts of surface functionalization can substantially affect the electronic dynamics of host materials. The corresponding justification has been added to the revised manuscript (**Page 10**, **Paragraph 2**).

To Reviewer 3:

The authors investigate how electron-phonon interactions in $Ti_3C_2T_x$ MXene can be tuned by modifying its surface with covalently attached Mo_3S_7 clusters. They use transient optical absorption to track photoinduced processes, specifically the excitation of coherent phonon modes. These measurements are complemented by optical pump–THz probe experiments that probe thermal relaxation. The topic is very interesting, and the data presented are of high quality, but at the current stage, the analysis and explanations provided are difficult to follow and unconvincing, raising concerns that the data may be over-interpreted.

Response: We appreciate the positive evaluation and constructive feedback of the reviewer, which are invaluable in improving the quality of our manuscript. All the concerns raised have been addressed through additional experiments and discussions. Details can be seen in the point-by-point responses below.

1. The abstract is not a good representation of the paper's observations. Specifically, the statement: "Femtosecond transient absorption spectroscopy reveals an ultrafast, nonthermal electron/energy extraction, accompanied by probe wavelength-dependent suppression of the coherent A1g phonon mode." — is this observed in Mo_3S_7-modified MXene only, or in both modified and unmodified samples? How does the addition of Mo_3S_7 impact this behavior? Could you please rephrase this to ensure that your findings are unambiguous?

Similarly, the sentence: "Complementary optical-pump terahertz-probe measurements confirm sub-100 fs energy extraction, with efficiency increasing from ~28.6% at 1.55 eV to ~38.2% at 3.88 eV, driven by improved energetic alignment between the hot electrons in $Ti_3C_2T_x$ and the conduction-band manifold of Mo_3S_7 nanoclusters." — Again, is the efficiency increase driven by the increase in excitation energy, or by the presence of the nanoclusters? This needs clarification.

Response: Thank you for the valuable comment. We would like to clarify that the ultrafast nonthermal electron/energy extraction and the probe-wavelength-dependent suppression of the coherent A_{1g} phonon mode are observed specifically in Mo_3S_7-anchored $Ti_3C_2T_x$, and are not as general features of pristine $Ti_3C_2T_x$. The incorporation of Mo_3S_7 nanoclusters introduces an ultrafast interfacial dissipation pathway that competes with the intrinsic nonthermal e–ph coupling in $Ti_3C_2T_x$, thereby suppressing coherent A_{1g} phonon generation.

We would also like to clarify that the observed increase in extraction efficiency is not solely driven by the increase in excitation energy, but arises from the combined effect of Mo_3S_7 nanocluster anchoring and excitation-energy-dependent energetic alignment. The presence of Mo_3S_7 nanoclusters is necessary

to enable the sub-100 fs interfacial energy/charge extraction, as evidenced by the absence of comparable ultrafast extraction dynamics in pristine $Ti_3C_2T_x$ under identical excitation conditions. Increasing the excitation photon energy populates higher-energy hot electrons in $Ti_3C_2T_x$, improving the energetic overlap with the conduction-band manifold of Mo_3S_7 nanoclusters and thereby facilitating more efficient interfacial transfer. This combined effect leads to the observed increase in extraction efficiency from ~28.6% at 1.55 eV to ~38.2% at 3.88 eV. The efficiency enhancement therefore reflects an excitation-energy-dependent modulation of interfacial transfer enabled by Mo_3S_7, rather than a simple consequence of higher excitation energy. Accordingly, the Abstract has been revised as follows.

"Femtosecond transient absorption spectroscopy reveals an ultrafast, nonthermal electron/energy extraction in Mo_3S_7-anchored $Ti_3C_2T_x$, accompanied by probe-wavelength-dependent suppression of the coherent A_{1g} phonon mode."

"Complementary optical-pump terahertz-probe measurements confirm sub-100 fs energy extraction, with efficiency increasing from ~28.6% at 1.55 eV to ~38.2% at 3.88 eV, reflecting an excitation-energy-dependent enhancement of interfacial energy extraction enabled by improved energetic alignment between hot electrons in $Ti_3C_2T_x$ and the conduction-band manifold of Mo_3S_7 nanoclusters."

2. The authors state that the nanoclusters enhance interfacial thermal conductance and facilitate heat dissipation between MXene layers, and that hot photoexcited electrons rapidly transition to the nanoclusters, disrupting the rapid transfer of energy to the MXene crystal lattice that would otherwise result in heating. However, their observation (based on transient absorption spectroscopy) that the behavior of non-modified and nanocluster-modified MXene films differs only under 266 nm (4.66 eV) excitation and remains virtually unchanged under 400 nm or 800 nm excitation, raises questions. While this wavelength-dependent behavior is very interesting in its own right, it appears to limit the validity of the broader conclusion that surface-anchored nanoclusters "introduce a rapid energy harvesting pathway that even competes with intrinsic e-ph relaxation" to only the deep UV excitation regime. Could the authors please clarify or reconcile this apparent contradiction?

Response: We thank the reviewer for this insightful comment. While the most pronounced differences between pristine $Ti_3C_2T_x$ and Mo_3S_7-anchored $Ti_3C_2T_x$ in TA spectroscopy are observed under 266 nm excitation, the apparent wavelength dependence does not imply that the nanocluster-mediated energy harvesting pathway operates exclusively in the deep-UV. Instead, it reflects the limitations of TA spectroscopy in resolving interfacial charge and/or energy transfer when multiple photoinduced processes spectrally and temporally overlap. Under 800 nm excitation, a reduction in the intensities of the PIA-2 and PIA-3 bands is still observed in Mo_3S_7-$Ti_3C_2T_x$ (**Fig. R15a**), qualitatively consistent with

the behavior under 266 nm excitation. Under 400 nm excitation (**Fig. R15b**), the PIA-2 and PIA-3 bands increase, and the PIA-2 band exhibits a blue shift, likely due to overlapping photoinduced absorption contributions from Mo_3S_7 that mask, rather than eliminate, the interfacial extraction signature. Such spectral superposition effects, including MXene carrier dynamics, nanocluster photo-response, and coherent phonons, limit the direct observability of interfacial energy extraction in TA spectra at longer wavelengths. To overcome the limitations of TA spectroscopy, we employed OPTP spectroscopy, which directly probes the generation, transport, and relaxation of free carriers. OPTP measurements reveal ultrafast sub-100 fs electron/energy extraction across all excitation wavelengths investigated, providing direct and quantitative evidence that the nanocluster-mediated energy harvesting pathway is operative across the entire excitation range.

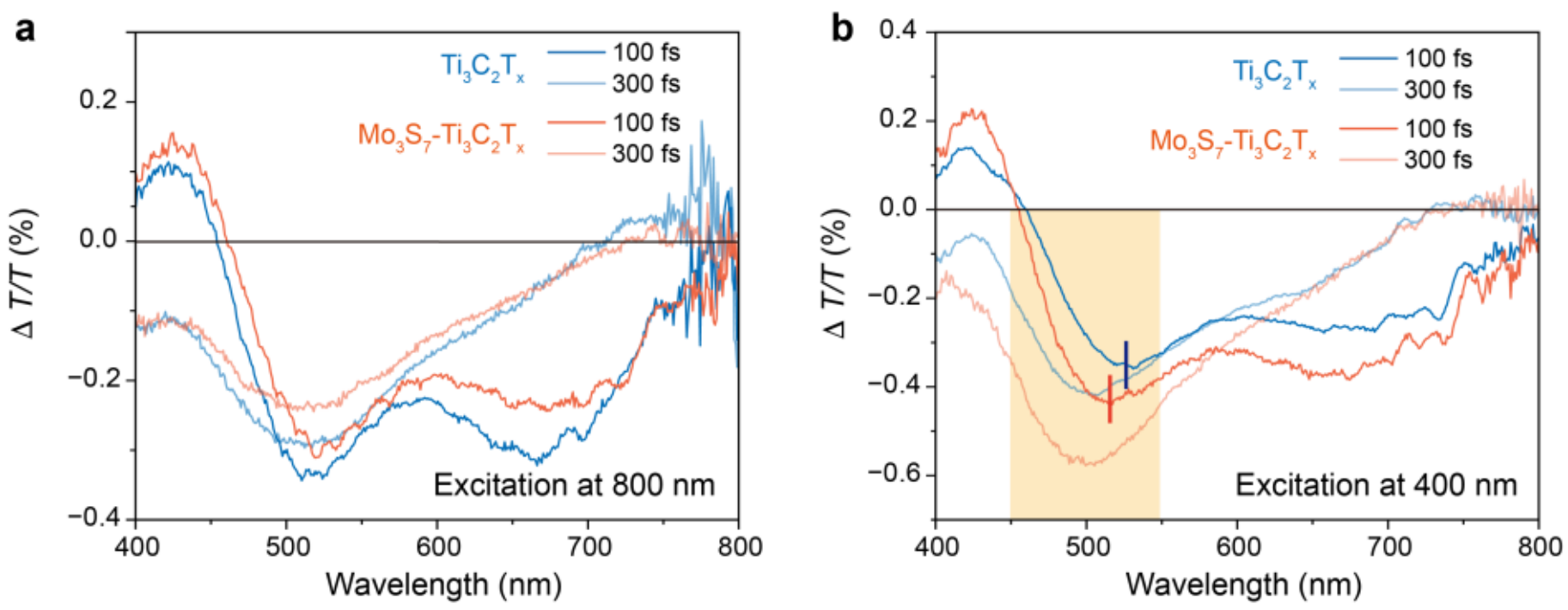

Fig. R15 $\Delta T/T$ spectra of $Ti_3C_2T_x$ and Mo_3S_7-$Ti_3C_2T_x$ at selected time delays of 100 fs and 300 fs under **a**, 800 nm and **b,** 400 nm excitation.

In addition, coherent A_{1g} phonon oscillations are clearly resolved only under 266 nm excitation due to instrumental and excitation-mechanism constraints. The A_{1g} mode at approximately 200 cm^{-1} corresponds to a vibrational period of ~167 fs, requiring an effective pump-probe temporal resolution substantially shorter than this timescale for efficient detection. Sub-50 fs UV pump pulses under 266 nm excitation provide impulsive excitation conditions, thereby preserving both the phase and amplitude of the ~6 THz coherent phonon oscillation and enabling clear temporal resolution. By contrast, under 400 nm and 800 nm excitation, the longer pump pulse durations (≈90–100 fs) combined with the intrinsic temporal chirp of the white-light continuum probe lead to temporal averaging that strongly suppresses high-frequency oscillatory components. Because interfacial energy and/or charge transfer is independently confirmed by OPTP across all excitation wavelengths, we conclude that the associated suppression of lattice heating and coherent phonon generation is a general effect and not restricted to deep-UV excitation. The corresponding discussion has been added to the revised manuscript (**Supplementary Fig. 11**).

3. The authors discuss how electrons excited by surface plasmon (SP) absorption at ~780 nm and those excited by interband transitions behave differently. Could they please elaborate on this point? Specifically, interband excitation would be expected to generate electrons and holes with less excess kinetic energy, as compared to excitation by free-carrier (intraband or plasmonic) absorption, which typically leads to hotter carriers. Is this what the authors observe, and how is this distinction reflected in their data?

Response: We appreciate the insightful question of the reviewer. While in many semiconductors, intraband or plasmonic excitation is often associated with hotter carriers, the situation in metallic MXenes with extremely high carrier densities is more nuanced and depends on how the absorbed energy is initially partitioned and redistributed. Under SP excitation in the 780–800 nm range, photon energy is deposited predominantly into the electronic system and rapidly redistributed among surrounding electrons via Landau damping and following e–e scattering. Owning to the extremely high carrier density of $Ti_3C_2T_x$ (~10^{21}–10^{22} cm^{-3}), this energy is spread across a large electronic heat capacity, resulting in a broad but relatively modest increase in the effective electronic temperature on sub-picosecond timescales. By contrast, IBT excitation at higher photon energies (<600 nm) promotes electrons across the band structure, generating electrons and holes in well-defined states. Although part of the photon energy is consumed by the optical transition, the excess energy initially remains within a smaller subset of optically excited carriers before being distributed across the full carrier population. This leads to more efficient electronic heating and stronger coupling to the lattice. Early ultrafast studies in $Ti_3C_2T_x$ have shown that the distinction between interband and plasmonic responses is governed primarily by their characteristic temporal dynamics rather than spectral position alone.[12] Interband-related transient absorption persists well beyond the e–ph thermalization time, whereas plasmonic responses in the 700–800 nm range are confined to ultrafast e–e timescales and decay rapidly before significant lattice heating occurs.

Consistent with this framework, our TA measurements reveal a short-lived (<200 fs) transient absorption feature near ~700 nm (PIA-3), followed by a bleaching-like signal near ~780 nm at later delays (**Fig. 3b**). This evolution reflects an ultrafast, e–e-driven perturbation of the plasmon resonance, where enhanced electronic scattering transiently broadens the plasmon line shape, producing apparent absorption enhancement on the resonance flank. As the electronic system thermalizes, this nonthermal perturbation diminishes and the plasmonic response evolves into a quasi-thermalized hot-electron state with reduced oscillator strength, leading to bleaching near the resonance center (~780 nm). By contrast, the interband-related transient absorption (PIA-1 and PIA-2) persists well beyond the e–e thermalization timescale and extends into the e–ph regime. The pronounced temporal persistence of this signal reflects

the fundamental distinction between interband excitations, which couple efficiently to the lattice, and plasmonic excitations, which are dominated by ultrafast electronic correlations. Complementary OPTP measurements further support this picture. The $\Delta E/E/N_{abs}$ signal directly reflects the evolution of the effective electronic temperature, revealing that IBT excitation generates a larger transient electronic temperature than SP excitation (**Fig. 5d**). This occurs because SP-injected energy is rapidly diluted across the exceptionally large electronic heat capacity of the MXene, whereas IBT excitation initially concentrates energy in a smaller carrier population prior to full thermalization. The corresponding discussion has been added to the revised manuscript (**Page 9**, **Paragraph 2**).

4. With 266 nm excitation, the main differences in the transient absorption spectra appear in the photoinduced absorption band (PIA-2) around 500 nm. Do the authors have insight into the origin and nature of this band?

Response: The PIA-2 band observed under 266 nm excitation can be attributed to photoinduced modulation of intrinsic interband transitions (IBTs) in metallic $Ti_3C_2T_x$. In the steady state, the visible optical response of $Ti_3C_2T_x$ is dominated by a dense manifold of strongly broadened and overlapping IBTs. Upon excitation, the electronic system is driven far from equilibrium, inducing pronounced changes in electronic self-energy, carrier distribution, and e–ph scattering rates. These nonequilibrium effects collectively lead to transient renormalization of the energies, line shapes, and spectral weight of pre-existing IBTs, resulting in an enhanced differential absorption signal around ~500 nm (PIA-2).

This interpretation is consistent with prior ultrafast studies of MXenes. In particular, Lioi *et al.*[12] employed simultaneous ultrafast transmission and reflection measurements on $Ti_3C_2T_x$, and demonstrated that probe wavelengths around 500–520 nm consistently exhibit positive transient absorption, explicitly attributed to pump-induced band (spectral-function) renormalization of interband transitions. This signal extends well beyond the e–ph thermalization time, clearly distinguishing it from ultrafast plasmonic or free-carrier responses and ruling out conventional excited-state absorption mechanisms. The assignment is further supported by first-principles calculations by Zhang *et al.*,[7] which identify dense manifolds of optically allowed interband transitions spanning the visible spectral range, including the 500–550 nm region. The spectral weight and line shape of the intrinsic abundance of IBTs can be transiently redistributed under strong optical excitation, consistent with the experimentally observed PIA-2 feature. The corresponding discussion has been added to the revised manuscript (**Page 9**, **Paragraph 2**).

5. The authors also note that the transient bleach at the plasmon resonance intensifies over the initial hundreds of femtoseconds, as clearly visible in the TA color maps. Could the authors comment on the

processes at play here? In your view, does this imply that the increase in lattice temperature, leading to surface plasmon (SP) bleaching, takes this long to develop? Or could this apparent delay be influenced by the overlapping contribution from the PIA-3 band, which also extends into this spectral range? Clarifying this would help disentangle the spectral dynamics and better contextualize the competing processes.

Response: We thank the reviewer for this insightful question regarding the apparent delayed intensification of the plasmon bleach observed in the TA color maps. This behavior does not indicate a slow buildup of lattice heating, but rather reflects the interplay of ultrafast electronic processes and overlapping spectral contributions. In our TA measurements, this early-time nonthermal electronic perturbation manifests as a transient photoinduced absorption feature (PIA-3) centered around ~700 nm and confined to sub-200 fs timescales. This feature arises from a transient broadening and reshaping of the plasmon resonance due to enhanced electronic scattering, producing an apparent absorption increase on the resonance flank. As the electronic system rapidly thermalizes, this nonthermal perturbation decays and the plasmonic response evolves into a quasi-thermalized hot-electron state with reduced overall oscillator strength, giving rise to the bleaching-like response near the resonance center (~780 nm). The apparent increase of the plasmon bleach during the first few hundred femtoseconds coincides with the decay of the overlapping PIA-3 feature. This interpretation is consistent with the observations by Lioi *et al.*,[12] as discussed in our response to your **Q3**. Therefore, the delayed emergence of the plasmon bleach primarily reflects the transition from an ultrafast, nonthermal electronic perturbation to a quasi-thermalized electronic state, together with spectral overlap effects, rather than the timescale of lattice heating. Genuine lattice heating mediated by e–ph coupling occurs on longer picosecond timescales, temporally distinct from the sub-200 fs dynamics discussed here. The corresponding discussion has been added to the revised manuscript (**Page 10**, **Paragraph 1**).

6. When discussing the differences in coherent phonon oscillations between nanocluster-modified and unmodified MXene, the authors observe a pronounced effect in the interband transition (IBT)-dominated spectral range (<580 nm), where coherent phonon generation is suppressed in the modified MXene. This effect is attributed to efficient interfacial hot carrier harvesting from MXene to the nanoclusters. Could the authors please elaborate on why IBT transitions are particularly affected following 266 nm excitation, while SP (plasmonic) transitions appear relatively unaffected?

Response: We thank the reviewer for this insightful question. Under 266 nm excitation, coherent phonons are generated through the same initial photoexcitation process, so the lattice displacement *Q(t)* is common across all probe wavelengths. The wavelength-dependent differences in coherent phonon signatures do not reflect different phonon-generation mechanisms, but instead arise from the probe-

dependent sensitivity of the transient optical response to lattice motion. Coherent phonons periodically modulate the lattice configuration, inducing a time-dependent modulation of the dielectric function and thus the TA signal. To first order, the oscillatory component can be expressed as **equation (R2)**, where $\Delta A(\omega,t)$ is the time-dependent change in the observable A at angular frequency ω, $A(\omega)$ is the equilibrium (unperturbed) value of the observable as a function of angular frequency, $\partial A(\omega)/\partial Q$ is the partial derivative describing how sensitive A is to the generalized coordinate Q, $Q(t)$ is the time-dependent generalized coordinate (small displacement from equilibrium), ω is the angular frequency, and t is time. The observed oscillation amplitude therefore depends not only on the phonon displacement itself, but critically on the sensitivity of the probed optical transition to lattice displacement, i.e., $\partial A/\partial Q$.

$$\Delta A\ (\omega,t) \approx \left(\frac{\partial A(\omega)}{\partial Q}\right) Q(t) \qquad \textbf{(R2)}$$

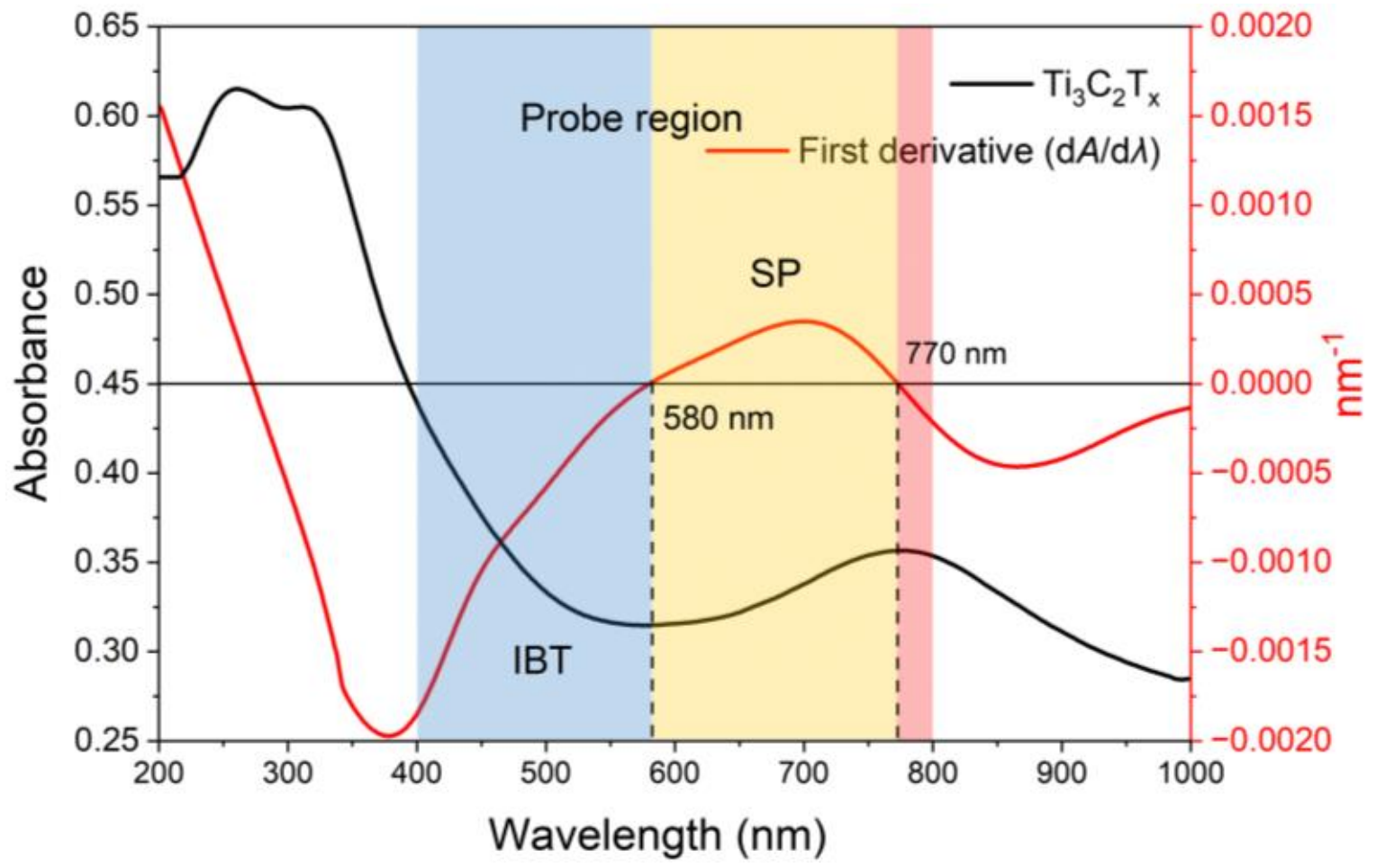

Fig. R16 Steady-state absorption spectrum of $Ti_3C_2T_x$ (A) and its first derivative with respect to wavelength ($dA/d\lambda$), which reflects the spectral sensitivity of the optical response to small perturbations of the resonance energy.

To quantify this sensitivity, we performed a first-derivative analysis of the steady-state absorption spectrum of $Ti_3C_2T_x$, $dA/d\lambda$ (**Fig. R16**). The derivative spectrum exhibits zero crossing near ~580 nm and ~770 nm, separating the IBT-dominated and SP-dominated regions. Specifically, $dA/d\lambda < 0$ in the 400–580 nm region (IBT dominated), while $dA/d\lambda > 0$ in the 580–770 nm region where the SP contribution becomes dominant. This sign reversal produces opposite oscillation phases on either side of the crossover wavelength, generating an amplitude node and π-phase inversion. Moreover, the larger magnitude of $|dA/d\lambda|$ in the IBT region indicates higher sensitivity of IBT-related transitions to lattice modulation. Absorption in the IBT-dominated region typically originates from discrete interband electronic transitions that are highly sensitive to lattice displacement through band-edge renormalization

and modulation of transition matrix elements. In contrast, the SP reflects a collective oscillation of a continuum of free carriers rather than discrete electronic transitions. Consequently, lattice displacements associated with coherent phonons do not efficiently modulate the SP in first order, and any coherent phonon-induced modulation of SP absorption proceeds only through weaker, higher-order effects such as small changes in carrier scattering. As a result, suppression of A_{1g} coherent phonon signatures upon nanocluster modification manifests most strongly in the IBT-dominated spectral region, where the optical response is more sensitive to lattice motion, while the SP-dominated region exhibits a comparatively muted response. The corresponding discussion has been added to the revised manuscript (**Supplementary Fig. 13**).

7. When analyzing the OPTP data under 800 nm excitation (Figs. 5a–c), the authors report a reduced overall amplitude of the photoinduced conductivity suppression in nanocluster-modified MXene, which they interpret as evidence of a rapid (~100 fs), non-thermal charge/energy extraction pathway. They also observe an accelerated decay of the photoexcited conductivity suppression in the modified sample. However, this interpretation appears to contradict the transient absorption (TA) data, where no significant changes are reported for 800 nm excitation (Figs. S8 and S9).

Furthermore, the conclusions from the OPTP measurements are based on changes in the magnitude of photoinduced conductivity, calculated as the change in amplitude per injected carrier density at different excitation photon energies. Could the authors clarify how this injected carrier density was determined? Since the steady-state absorption spectrum (Fig. 3a) is normalized to the SPR peak, it is difficult to assess the role of potential differences in absolute absorption between the modified and unmodified films at various excitation wavelengths.

Response: We appreciate the constructive comment of the reviewer. While the TA spectra under 800 nm excitation show much less pronounced differences between pristine and Mo_3S_7-anchored $Ti_3C_2T_x$, this does not contradict the OPTP results. Rather, it reflects the fundamentally different physical observables probed by the two techniques. TA spectroscopy probes spectral changes in optical transitions, which in metallic MXenes arise from a superposition of interband transitions, plasmonic responses, and coherent phonon modulations. Under 800 nm excitation, the probe spectral window is dominated by plasmonic and free-carrier contributions, which are intrinsically less sensitive to interfacial carrier or energy extraction. Nevertheless, a reduction in the intensities of the PIA-2 and PIA-3 bands is still observed in Mo_3S_7-$Ti_3C_2T_x$ under 800 nm excitation, qualitatively consistent with the behavior under 266 nm excitation (**Fig. R15a**). By contrast, OPTP directly probes the generation, transport, and relaxation of free carriers through the transient photoconductivity, making it inherently more sensitive to ultrafast nonthermal carrier and energy extraction processes, even when the

corresponding spectral signatures in TA are weak or obscured. Under 800 nm excitation, the pronounced reduction of photoconductivity amplitude and its accelerated recovery in the modified sample provide direct evidence for an ultrafast interfacial energy and/or charge extraction pathway, despite the subtle TA signatures. TA and OPTP should therefore be regarded as complementary rather than contradictory. TA highlights wavelength-dependent spectral modulations and coherent phonon dynamics, whereas OPTP provides a direct and quantitative probe of ultrafast carrier extraction and energy dissipation.

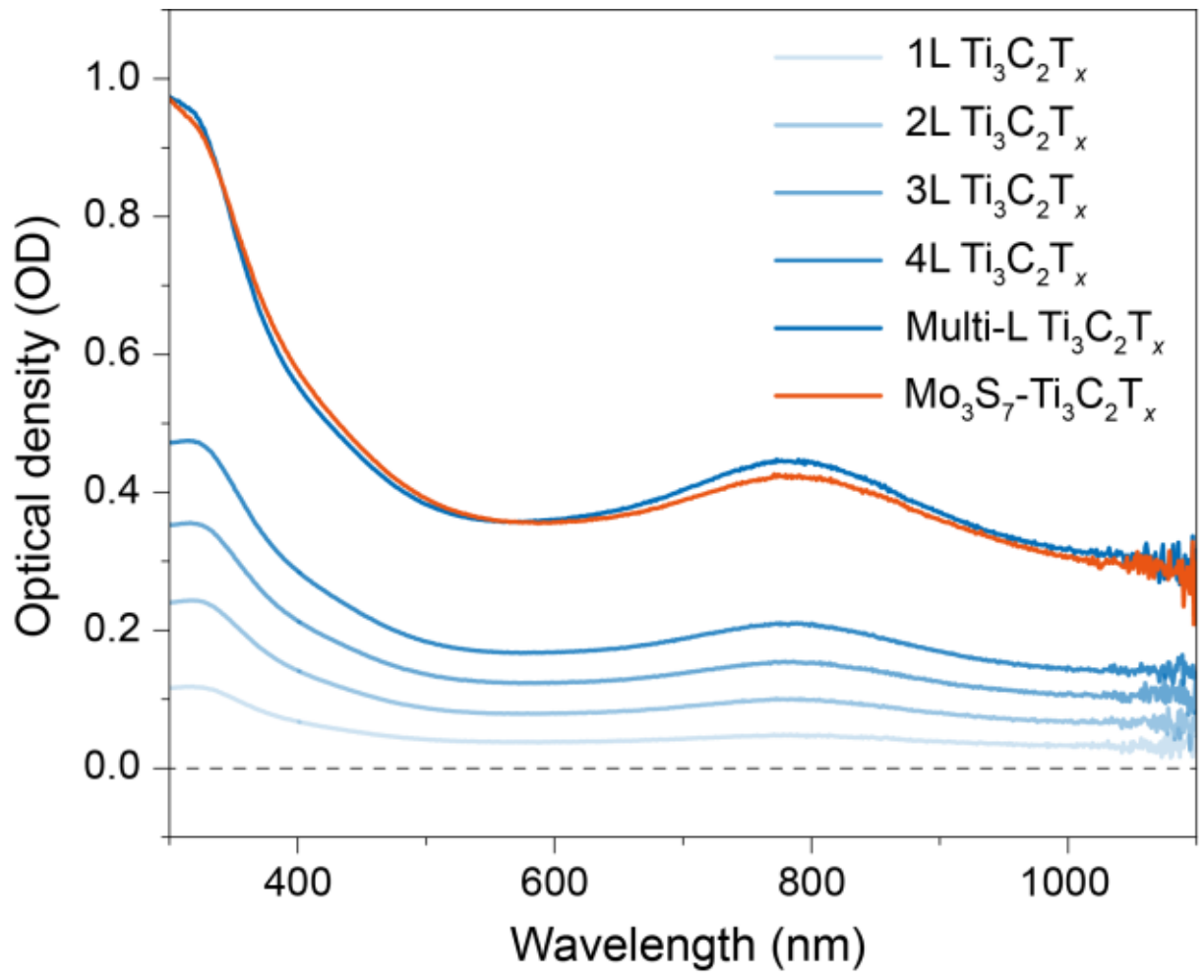

Fig. R17 Layer-dependent UV–Vis optical density (OD) spectra of assembled $Ti_3C_2T_x$ films (as references), along with those of $Ti_3C_2T_x$ and Mo_3S_7-$Ti_3C_2T_x$ samples used in the OPTP measurements, revealing comparable thicknesses of approximately 8.5 layers for both $Ti_3C_2T_x$ and Mo_3S_7-$Ti_3C_2T_x$ samples. These OD spectra were used to determine the absorbed photon density (N_{abs}) at the pump wavelengths.

Regarding the determination of the injected carrier density, we assume that the reviewer referred to the determination of the absorbed photon density (N_{abs}). In the OPTP analysis, photoinduced conductivity changes were normalized by N_{abs} (i.e., $\Delta E/E/N_{abs}$), rather than by the incident photon fluence. N_{abs} was estimated using the absolute absorption at the excitation wavelength for each sample, determined from the steady-state absorption spectra shown in **Fig. R17**. These spectra were measured under identical experimental conditions (same substrate, comparable thickness, and measurement geometry). After this normalization, the absorbed photon densities at each pump wavelength are comparable for $Ti_3C_2T_x$ and Mo_3S_7-$Ti_3C_2T_x$. Consequently, the observed differences in the OPTP dynamics reflect intrinsic differences in ultrafast carrier and energy dissipation processes induced by nanocluster anchoring. In **Fig. 3a**, the steady-state absorption spectra are presented in normalized (arbitrary) units to emphasize

spectral shape differences and the influence of Mo_3S_7 anchoring. The corresponding discussion has been added to the revised manuscript (**Supplementary Fig. 15)**.

References

1. Batool, S. et al. Surface anchoring and active sites of $[Mo_3S_{13}]^{2-}$ clusters as co-catalysts for photocatalytic hydrogen evolution. *ACS Catal.* **12**, 6641 (2022).

2. Zhao, J. et al. Evidence of surface-mediated carrier-phonon scattering in MXene. *ACS Nano 17,* 23714 (2023).

3. Zhang, Q. *et al*. Simultaneous capturing phonon and electron dynamics in MXenes. *Nat Commun* **13**, 7900 (2022).

4. Li, G. et al. Dynamical control over terahertz electromagnetic interference shielding with 2d $Ti_3C_2T_y$ mxene by ultrafast optical pulses. *Nano Lett.* **20**, 636 (2019).

5. Strait, J. H. et al. Very slow cooling dynamics of photoexcited carriers in graphene observed by optical-pump terahertz-probe spectroscopy. *Nano Lett.* **11**, 4902-4906 (2011).

6. Kar, S. et al. Tuning photoinduced terahertz conductivity in monolayer graphene: Optical-pump terahertz-probe spectroscopy. *Phys. Rev. B* **90**, 165420 (2014).

7. Zhang, Q. *et al.* Real-time observation of two distinctive non-thermalized hot electron dynamics at MXene/molecule interfaces. *Nat. Commun*. **15**, 4406 (2024).

8. Shen, S. et al. Interplay of Ultrafast Electron–Phonon and Electron–Electron Scattering in $Ti_3C_2T_x$ MXenes: Ab Initio Quantum Dynamics. *Nano Lett.* **25**, 7517 (2025).

9. Zhang, J. et al. Interface-Tailored Secondary Excitation and Ultrafast Charge/Energy Transfer in $Ti_3C_2T_x$-MoS_2 Heterostructure Films. *J. Am. Chem. Soc.* **147**, 10012 (2025).

10. Tagliabue, G. et al. Ultrafast hot-hole injection modifies hot-electron dynamics in Au/p-GaN heterostructures. *Nat. Mater.* **19**, 1312 (2020).

11. Zheng, W. et al. Band transport by large Fröhlich polarons in MXenes. *Nat. Phys.* **18**, 544 (2022).

12. Lioi, D. B. et al. Simultaneous Ultrafast Transmission and Reflection of Nanometer-Thick $Ti_3C_2T_x$ MXene Films in the Visible and Near-Infrared: Implications for Energy Storage, Electromagnetic Shielding, and Laser Systems. *ACS Appl. Nano Mater.* **3**, 9604 (2020).
